# Optimizing Water-Based Extraction of Bioactive Principles of Hawthorn: From Experimental Laboratory Research to Homemade Preparations

**DOI:** 10.3390/molecules24234420

**Published:** 2019-12-03

**Authors:** Phu Cao Ngoc, Laurent Leclercq, Jean-Christophe Rossi, Isabelle Desvignes, Jasmine Hertzog, Anne-Sylvie Fabiano-Tixier, Farid Chemat, Philippe Schmitt-Kopplin, Hervé Cottet

**Affiliations:** 1 IBMM, University of Montpellier, CNRS, ENSCM, 34059 Montpellier, France; tech.ngocphu@gmail.com (P.C.N.); isabelle.desvignes@umontpellier.fr (I.D.); 2 Analytical BioGeoChemistry, Helmholtz Zentrum Muenchen, 85764 Neuherberg, Germany; jasmine.hertzog@helmholtz-muenchen.de (J.H.); schmitt-kopplin@helmholtz-muenchen.de (P.S.-K.); 3 Analytical Food Chemistry, Technische Universität Muenchen, 85354 Freising, Germany; 4 University of Avignon, INRA, UMR408, GREEN Extraction Team, F-84000 Avignon, France; anne-sylvie.fabiano@univ-avignon.fr (A.-S.F.-T.); farid.chemat@univ-avignon.fr (F.C.)

**Keywords:** hawthorn, water-based extraction, procyanidin, polyphenol, flavonoid, standardization, extraction mode, infusion, granulometry

## Abstract

Hawthorn (Crataegus) is used for its cardiotonic, hypotensive, vasodilative, sedative, antiatherosclerotic, and antihyperlipidemic properties. One of the main goals of this work was to find a well-defined optimized extraction protocol usable by each of us that would lead to repeatable, controlled, and quantified daily uptake of active components from hawthorn at a drinkable temperature (below 60 °C). A thorough investigation of the extraction mode in water (infusion, maceration, percolation, ultrasounds, microwaves) on the yield of extraction and the amount of phenolic compounds, flavonoids, and proanthocyanidin oligomers as well as on the Ultra High Performance Liquid Chromatography (UHPLC) profiles of the extracted compounds was carried out. High-resolution Fourier transform ion cyclotron resonance mass spectrometry was also implemented to discriminate the different samples and conditions of extraction. The quantitative and qualitative aspects of the extraction as well as the kinetics of extraction were studied, not only according to the part (flowers or leaves), the state (fresh or dried), and the granulometry of the dry plant, but also the stirring speed, the temperature, the extraction time, the volume of the container (cup, mug or bowl) and the use of infusion bags.

## 1. Introduction

The extraction of bioactive principles from medicinal plants is often poorly controlled and depends on a large number of factors such as the extraction temperature, extraction time, particle size of the dry plant, and the amount and origin of the plant introduced into the extraction solvent [1]. This is particularly true when people are doing plant infusions at home, with poor control of the experimental conditions of extraction leading to low repeatability/reproducibility. Outside the laboratory, neither the plant weight, granulometry, temperature, nor the water volume are usually well controlled. A consequence of this is that the intake dose of biologically relevant components extracted from the medicinal plant may significantly vary and remain uncontrolled. This is one of the reasons why Western medicine is often reticent to promote the use of home-prepared medicinal plants and may prefer the prescription of standardized and commercialized plant-based extracts. However, standardized extracts of plants and/or plant-based medicines are generally expensive, restricting access to a limited part of the population [2,3]. Therefore, it seems crucial to investigate the impact of the experimental conditions of extraction (including conditions close to home-prepared extractions) on the daily intake dose and its reproducibility. It is also important to normalize it in comparison with standardized medicines. Moreover, some recent studies have demonstrated that tea drinkers in modern life have more esophagus cancer when compared to the rest of the population because they drink it too hot (>60 °C) [4,5]. It is consequently important to study and optimize simple and straightforward protocols of extraction using the minimum of equipment, leading to repeatable, quantifiable, and safe daily uptakes of active components from a given medicinal plant.

Hawthorn (Crataegus) is a bushy shrub, usually spiny, with light green leaves, white umbellate flowers, and edible red fruits [6]. It is readily available in the wild in temperate areas of Eurasia and North America, with over 280 species listed. In traditional Chinese medicine, the fruit is used for its stimulating properties of digestion and gastric function and for the improvement of blood circulation [6,7,8,9,10,11,12]. In Europe and North America, flowering tops (leaves and flowers) are used for their astringent, antispasmodic, cardiotonic, diuretic, hypotensive, vasodilative, sedative, antiatherosclerotic, and antihyperlipidemic properties [6,7,8,9,12,13,14,15,16,17,18,19,20,21,22,23]. Most of the experimental works published on Crataegus have focused on the extraction, quantification, and identification of phenolic compounds, flavonoids, and tannins to which the merit of these pharmacological effects is attributed [10,12,24,25,26,27,28,29,30,31,32,33,34,35,36,37,38,39,40,41,42,43,44,45,46,47,48,49,50,51,52,53,54,55,56,57,58]. These bioactive principles are usually extracted in ethanol, methanol, or alcohol/water mixtures at different temperatures and extraction times by using various extraction modes, essentially Soxhlet [30,45,46,52,56,58,59,60,61,62,63,64,65,66,67,68], maceration [24,25,26,27,28,31,33,38,43,47,49,50,51,57,60,63,69,70,71,72,73,74,75,76,77,78], or ultrasonic [29,32,35,36,39,40,41,42,56,60,63,77,79,80,81,82,83], but also decoction [33,84,85], infusion [33,44,61,66,84], percolation [10,28,53,86,87], microwave [60], or even supercritical carbon dioxide without any solvent [77,88,89]. Ultrasonic and microwave have been found to be the most efficient extraction modes [60,63,77]. The influence of Crataegus species [26,27,35,41,43,46,65,70,73,79,83,89,90], the harvest area [20,27,32,36,41,54,55,56,65,70,79,90], and the plant organ (flowering tops, flowers, leaves, fruits at various states of ripening) [8,25,27,32,35,36,37,40,41,43,47,48,51,56,61,65,66,70,72,73,74,77,82,84,89,90,91,92,93,94] have also been largely studied in the literature. Before extraction, the plant is generally dried, crushed, and powdered using a grinder (typically a mortar or a coffee grinder, when mentioned) [24,25,28,29,35,36,38,39,40,41,42,43,45,46,47,48,49,50,51,56,57,59,60,61,63,64,65,66,68,70,71,72,75,76,78,79,80,81,82,85,86,90,93,95,96], but, to our knowledge, no article has dealt with the influence of plant granulometry on the extraction yield. The use of an organic solvent (typically ethyl acetate) or the presence of an alcoholic co-solvent with water was found to improve the extraction yield of bioactive principles when compared to extraction in pure water [28,29,38,53,60,61,78,81,87,90]. In fact, few articles have focused on the extraction modes in water [28,29,30,33,37,41,44,53,62,66,76,79,84,85,87,96] and none have addressed all of the parameters in a single study for the investigation of hawthorn extraction mode and hawthorn extract analysis. Crataegus extracts have been studied and are still currently studied in clinical trials, showing their effectiveness in treating mild heart failure without side effects [6,7,8,9,14,15,17,18,19,20,22]. Other biological tests have been performed on animals to investigate the impact of hawthorn extracts on various illnesses including cancers [33,55], atherosclerosis [48,58,75], thrombosis [52,59], cataract [97], anxiety [45], heart diseases [13,68,72,75,95], stomach diseases [10], neurological diseases [29], liver diseases [48,67,72], or microbial diseases [10,43,50,73].

In this work, a thorough investigation of the extraction mode in water (infusion, maceration, percolation, ultrasounds, microwaves) on the yield of extraction and the amount of phenolic compounds, flavonoids, and proanthocyanidin oligomers as well as the UHPLC profiles of the extracted compounds was carried out on hawthorn dry plants. The quantitative and qualitative aspects of the extraction as well as the kinetic of extraction were studied according to the part (flowering tops or flowers), the state (fresh or dried), and the granulometry of the dry plant (from ~200 µm to 5 mm). The impact of the extraction parameters such as the stirring speed (250 to 1000 rpm), the temperature (20 to 100 °C), the extraction time (5 to 30 min) as well as practical parameters such as the volume of the container (cup, mug, or bowl) and the use (or not) of infusion bags have also been investigated. High-resolution Fourier transform ion cyclotron resonance mass spectrometry (FT-ICR-MS) was also implemented to obtain more qualitative information on the chemical compositions, thus allowing the different samples and conditions of extraction to be discriminated.

One of the main goals of this work was to find a well-defined optimized extraction protocol, usable by each of us, that leads to repeatable, controlled and quantified daily uptake of active components from hawthorn at a drinkable temperature (60 °C).

## 2. Results and Discussion

Five different modes (infusion, maceration, ultrasonic (US), microwave (MW), and percolation) were compared to extract the water-soluble bioactive components contained in hawthorn. This study was voluntarily restricted to water as the extracting solvent since the final optimized protocol should be useable by anyone and kept as simple as possible (absence of non-drinkable solvents). The protocols of extraction according to each extraction mode are described in the experimental section (see Section 3.3, Section 3.4, Section 3.5, Section 3.6 and Section 3.7) and a picture of each experimental setup is also provided in the Supplementary Materials, see Figure S2). The temperature of extraction, stirring speed, extraction time, plant part (flowering tops or flowers), nature (dry, fresh), and granulometry were studied. Table 1 gathers the different modalities that were studied in this work for each experimental parameter according to the extraction mode. For each experimental condition of extraction, the kinetic of extraction was first studied by monitoring the UV absorbance upon the extraction time (Section 3.1). Then, the extraction yields were determined and the specific contents in the polyphenols, flavonoids, and procyanidin oligomers were measured by complexation and spectrophotometry at 10 min and 30 min extraction times. All extractions performed in triplicate (three independent extractions). Analyses using UHPLC coupled to electrospray ionization mass spectrometry (ESI-MS) and (−)ESI-FT-ICR-MS were also performed to gain better insights into the differences between extraction modes/natures of the plant.

### 2.1. Influence of the Extraction Mode on the Kinetics of Extraction and on the Global Extraction Yields Using Raw Dry Plants

**Figure 1:**
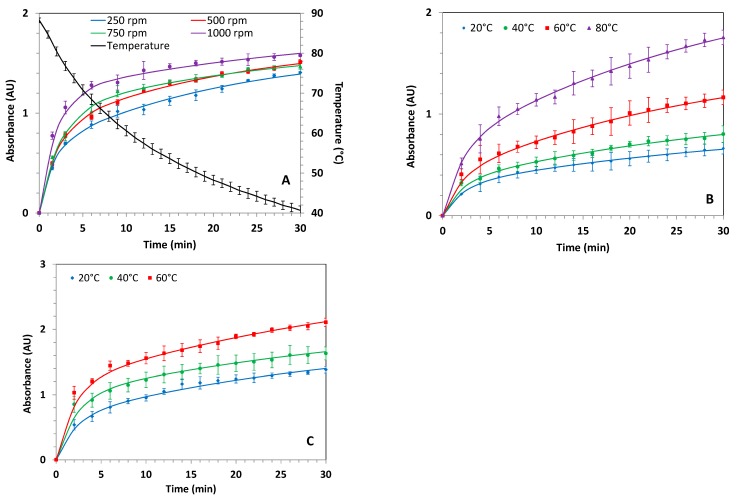
Kinetics of extraction of raw flowering tops hawthorn (lot no. 20335) monitored by UV absorbance at 198 nm for various extraction modes. (**A**) Infusion mode at 250 rpm, 500 rpm, 750 rpm, and 1000 rpm stirring speed, with the corresponding temperature profile obtained at 500 rpm. (**B**) Maceration mode at 20 °C, 40 °C, 60 °C, and 80 °C and at 500 rpm stirring speed. (**C**) Ultrasonic (US) mode at 20 °C, 40 °C, and 60 °C and at 250 rpm stirring speed. In all cases, 2.5 g of raw hawthorn in 250 mL water was used. A 100 μL sample of the solution was taken and added to 4 mL ultrapure water before each UV measurement. Error bars are ±1 SD on *n* = 3 repetitions of independent extractions. If the absorbance values were above 1.7, dilution in 8 mL (instead of 4 mL) was used, but the experimental values were then multiplied by two to allow for a comparison with dilutions in 4 mL.

The kinetics of extraction were compared on raw (non-ground) hawthorn (lot no. 20335) for infusion, maceration, and US-assisted extraction modes by monitoring the UV absorbance at 198 nm (Figure 1). A 100 µL aliquot was taken from the reactor at different extraction times and diluted in 4 mL (or 8 mL to avoid saturation of the UV detector) water (see Section 3.9). The monitoring wavelength was chosen to maximize the number of components that could be detected. Such simple experiments are very useful in practice to optimize the extraction time and the yield of extraction. The effect of stirring speed (magnetic stirring) was studied for the infusion mode, while the effect of temperature was investigated for maceration and ultrasonic-assisted modes. The kinetics of extraction could not be studied for MW-assisted and percolation modes because the equipment did not allow simple and repeated sampling during the extraction. An extraction time of 30 min was considered as the maximum reasonable extraction time, therefore the UV monitoring was stopped after 30 min. Extraction yields (in mass % of the introduced plant) were determined on independent experiments from the UV monitoring by evaporation and freeze-drying of the whole extract at 10 min (or 30 min) extraction times.

In the case of infusion mode (see Figure 1A), the temperature decreased in the reactor from about 90 °C to 40 °C after 30 min of extraction at 500 rpm. The drinkable temperature (60 °C) to avoid any side effects in health such as an increasing risk of esophageal carcinoma [4,5] was reached after 10 min of extraction (250 mL water). The increase in stirring speed from 250 rpm to 1000 rpm increased the kinetics of extraction, as seen in the absorbance monitoring (Figure 1A), while the maximum absorbance (and the extraction yield) were less affected by the stirring speed. All of the kinetic curves (absorbance *A(t)* vs. extraction time *t*) were fitted according to the following equation (see plain lines in Figure 1) using Excel solver:

(1) $$A(t)=A_{\infty }-A_{1}\exp(-\frac{t}{\tau_{1}})-\left(A_{\infty }-A_{1}\right)\exp(-\frac{t}{\tau_{2}})$$

where $A_{\infty }$ is the maximum absorbance at infinite extraction time; $A_{1}$ is a fitting parameter corresponding to an intermediate extraction plateau; and *τ*_1_ and *τ*_2_ are two characteristic extraction times. These two characteristics times were required to obtain a better fit of the experimental curves, as observed by the first plateau of absorbance located between 8 and 10 min, depending on the stirring speed. All fitting parameters are given in the Supplementary Materials (see Table S2). To allow for a fast comparison of the kinetics of extraction between the extraction conditions, it was convenient to simply calculate from the fitting curve the time *t_70%_* to obtain 70% of the absorbance value at the highest extraction time (30 min). For infusions, *t_70%_* was about two times lower at 1000 rpm stirring speed (4 min) vs. 9 min at 250 rpm (see Table 2). The extraction yield was found to be similar at 10 and 30 min extraction times and about 16% (expressed in mass % of the solid extract compared to the initial mass of dry plant), showing that 10 min of infusion is sufficient to extract the maximum bioactive principles from dry raw hawthorn plant.

For the maceration mode, the same experimental set-up as the one used for infusion was used except that the extraction temperature was kept constant by using a temperature sensor and regulator (see Section 2.4). The stirring speed was set to 500 rpm (magnetic stirring) and different temperatures were investigated (20, 40, 60, and 80 °C). The corresponding UV kinetic monitoring is displayed in Figure 1B. The kinetics of extraction were slower than for the infusions, but similar for all maceration temperatures, with a typical *t_70%_* of about 12–13 min, regardless of the temperature (Table 2). Even at 80 °C maceration, the kinetics were significantly slower than for infusion and the extraction yield at 30 min (16%) was similar to that of infusion at the same stirring speed, despite a ~17% higher absorbance at 30 min when compared to infusion mode. The extraction yields and maximum absorbance at 30 min rapidly dropped with decreasing extraction temperature (only 9.7% yield at 20 °C for 30 min extraction). By selecting the maceration temperature at 60 °C (drinkable temperature), the extraction yield at 10 min was found to be lower (12%) than for infusion mode (16%) after 10 min, corresponding to the same final temperature (60 °C).

In the case of US-assisted mode, the stirring speed was set to 250 rpm (mechanical stirring). The higher the temperature (i.e., from 20 °C to 60 °C), the faster the extraction kinetics, as expected. Similar to infusion mode, *t_70%_* was less than 10 min, regardless of the temperature (Table 2). The absorbance values at 30 min were about two times higher than maceration mode at the same temperature. At 40 °C and 60 °C, the absorbance at 30 min was also higher than for infusion mode. By selecting the temperature at 60 °C (drinkable temperature), the extraction yield was found to be lower at 10 min (17%) than at 30 min (21%)of extraction time, but still higher than for the maceration mode at the same temperature (12% after 10 min), which was similar to infusion after 10 min (16%), but significantly higher after 30 min.

As for the MW-assisted mode, the power was set to 300 W. The temperature increased rapidly to reach 78 °C at 5 min, 95 °C at 10 min, and 97 °C at 30 min. The extraction yields at 10 and 30 min (17% and 22%) were found to be similar to those for US-assisted mode at 60 °C, regardless of the extraction time (Table 2), but with much higher final temperatures in the case of MW-assisted mode.

For the percolation mode, the extraction yield at 10 min was found to be even slightly higher (18%) than that for the US-assisted and MW-assisted modes (17%) (Table 2). This can be explained by the high temperature (100 °C), which was set from the beginning of the extraction. No yield value was obtained at 30 min extraction time because of the clogging at the end of the extraction.

In conclusion, at 10 min extraction time on raw materials, the infusion, US, MW, and percolation modes led to relatively similar extraction yields comprised between 16% and 18%. However, at 30 min extraction time, the US and MW modes were significantly more efficient (21–22%) than infusion (16%) or maceration.

### 2.2. Influence of the Plant Grinding on the Extraction Kinetics and on the Global Extraction Yield

In a second set of experiments, similar extractions were performed on ground (using 1 mm mesh size grinder) hawthorn flowering tops of the same lot (lot no. 20335). Clearly, the ground plant led to much faster kinetics of extraction with the *t_70%_* lower than 1.5 min (Table 2) for all extraction modes, regardless of the stirring speed and temperature, except for the two lowest maceration temperatures (20 °C and 40 °C). The kinetics of extraction were so fast that it was not possible to accurately determine *t_70%_* (see Figure S3 for the UV monitoring). Extraction yields were similar at 10 and 30 min of extraction times due to fast extractions (see Table 2), but slightly higher values were obtained for MW and US-assisted modes (23–25%) than for infusion mode (22–23%) and maceration mode (20–21%). The extraction yields obtained from ground material were much higher for all extraction modes compared to raw materials (+42% for infusion and ultrasonic extractions; +34% for microwaves and up to +71% for maceration at 60 °C), except for percolation.

**Figure 2:**
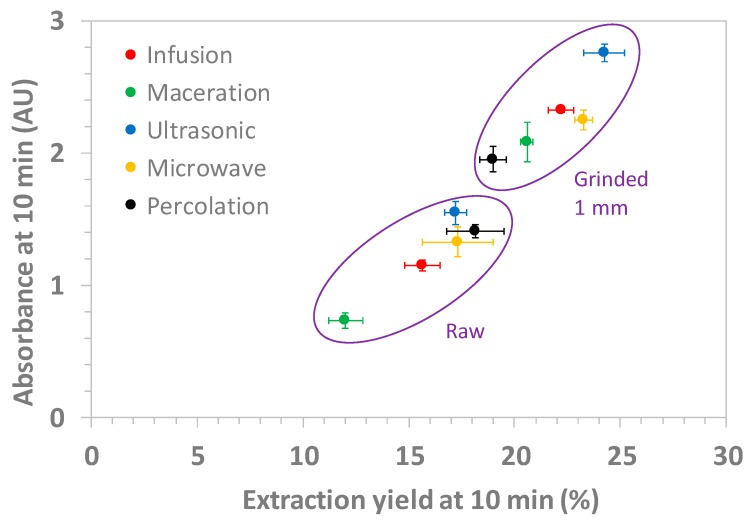
UV absorbance values at 198 nm versus the extraction yield at 10 min extraction time for various extraction modes. Maceration and ultrasonic assisted modes at 60 °C. In all cases, 2.5 g of hawthorn material in 250 mL water was used. Dry hawthorn lot number: 20335. Ground material with 1 mm grid mesh size. Error bars are ±1 SD on *n* = 3 repetitions of independent extractions.

Figure 2 displays the UV absorbance obtained at 198 nm for 10 min of extraction time as a function of the extraction yields at the same extraction time for the five extraction modes for raw/ground plants. There was a linear correlation between the UV absorbance and the extraction yield. However, surprisingly, this correlation did not extrapolate to zero extraction yield at zero absorbance, suggesting that 7–8% of the initial mass of the dry plant corresponded to some easily extracted UV-transparent compounds. Similar trends/results were obtained at 5 min and 30 min extraction times (see Figure S4). From Figure 2, the most efficient extraction mode appears to be the US-assisted mode at 60 °C, closely followed by the MW and infusion modes for the ground materials.

Compared to the other experimental parameters (extraction mode, stirring speed, and to a lower extent, extraction temperature), the plant granulometry was by far the most important factor to speed-up the kinetics and to maximize the yield of extraction. Between the lowest value (maceration on raw material) and the highest value (ultrasonic on ground plants) obtained at the same drinkable temperature (60 °C), a factor of two was found on the extraction yield, demonstrating the importance of the protocol of extraction, even for a similar temperature of extraction.

A good news from this study, from a practical point of view, is that infusion mode appears to be the best compromise among all the extraction modes because (i) it is probably the most simple mode to implement, (ii) the kinetics of extraction was very fast and the extraction yield at 10 min was not very different from the best values obtained in this study (22% for infusion vs. 23–24% for MW and US-assisted modes; non-significant differences between extraction modes at the 0.97 confidence level by one-way ANOVA), and (iii) the temperature profile of infusion was much more gentle than for MW, which is preferable to avoid the temperature degradation of biologically active components.

### 2.3. Quantification of Total Polyphenol, Flavonoid, and Proanthocyanidin Contents. Comparison with Commercialized Standardized Extracts and Antioxidant Activity

After extraction, the plant extracts were evaporated, lyophilized, and kept in the dark at −18 °C for better conservation prior to analysis. The values for the total amounts of polyphenol (TPC), flavonoid (TFC), and proanthocyanidin oligomers (OPC) contents in the dry plant extracts were determined by colorimetric methods (see Section 3.10, Section 3.11 and Section 3.12) and expressed as equivalent content in gallic acid (GA) for TPC, quercetin (Q) for TFC, and cyanidin (CY) for OPC. The numerical values are reported in Table 2 for 10 min and 30 min extraction times (*n* = 3 repetitions on three independent extractions).

As expected, the extraction yields of polyphenols, flavonoids, and proanthocyanidin oligomers were much higher for ground material than for raw material. TPC (resp. TFC) contents at 10 min extraction varied between 12.6 and 34.7 mg eq. GA/g dry plant (resp. 1.47 and 3.93 mg eq. Q/g dry plant), when comparing the worst (maceration, 60 °C, raw) and the best (microwaves, ground) figures of merits. This represents an enhanced extraction factor (EF) of about 2.7, which even reached up to 3.8 for OPC. This improvement in the extraction of the components of interest (TPC, TFC, and OPC) was even more pronounced than the increase in the global extraction yield, which was only affected by a factor of ~2. Clearly, grinding the dry plant is the most important parameter to increase the extraction yields for all of the quantified components. At 10 min extraction time, for ground dry hawthorn, 27–35 mg equivalent GA (TPC)/g dry plant, 2.9–4.0 mg equivalent Q (TFC)/g dry plant, and 3.6–4.3 mg equivalent CY (OPC)/g dry plant were extracted, which is in good agreement with the values usually reported in the literature for the same plant [20,24,33,34,53,61]. MW and US modes gave the best extraction yields, either for raw and ground plants, which is in good agreement with the literature [60], but the differences with infusion mode were only limited (only 5–10% differences in the TPC, TFC, and OPC extraction yields).

As a matter of comparison with commercialized standardized extracts (see Table 3), the dry extract content and the TPC, TFC, and OPC contents obtained from a single infusion (at 500 rpm) of 2.5 g of 1 mm ground hawthorn in 250 mL water were determined. One infusion produced about 555 mg of dry extract containing 82 mg equivalent GA (TPC), 8.6 mg equivalent Q (TFC), and 9.8 mg equivalent CY (OPC). These values are, for instance, similar to the dry extract content of a Cardio Max WS1442^®^ tablet (450 mg per tablet) and similar to the TFC intake given by 5 mL of standardized hawthorn plant extract (EPS Phytoprevent^®^, 7.5–12.5 mg eq. Q). The contents in TPC, TFC, and OPC given by the suppliers are gathered in Table 3 (when known) for four different standardized commercial products (tablets of dry hawthorn extracts Faros 300 LI132^®^ and Cardio Max WS1442^®^, EPS Phytoprevent^®^ or Crataegisan^®^ Bioforce extracts), even if the standardization is not always performed using the same compounds of reference. On the whole, it can be concluded that one to two infusions per day of 2.5 g of ground dry hawthorn flowering tops provide similar quantities of hawthorn extracts and TPC/TFC/OPC contents when compared to the advised posology of the standardized formulations.

**Figure 3:**
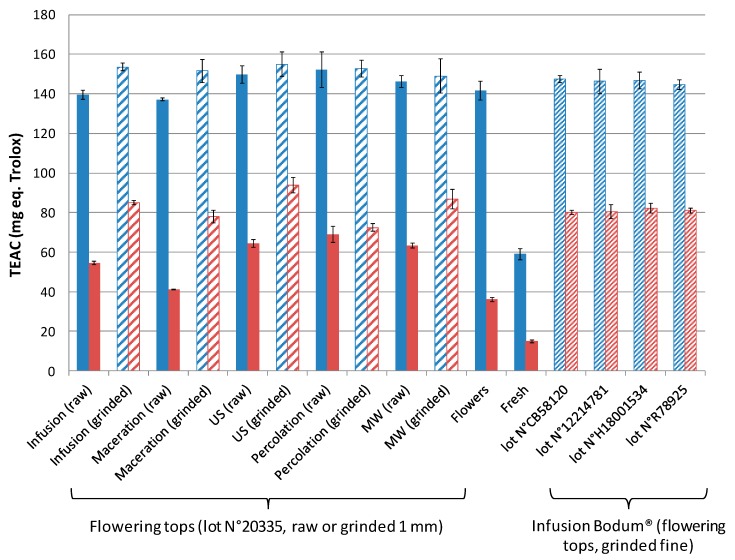
Antioxidant activities of hawthorn extracts obtained by Trolox equivalent antioxidant capacity (TEAC) assays. Dry plant: Lot Nos. 20335 (flowering tops) and 20334 (flowers only), except for the Bodum^®^ infusion (as indicated on the graph). In blue: TEAC in mg eq. Trolox per g of extract. In red: TEAC in mg eq. Trolox per 2.5 g plant. US and maceration modes performed at 60 °C. Hatched lines = ground materials.

The antioxidant activity quantified by the Trolox equivalent antioxidant capacity (TEAC) assay is displayed in Figure 3, expressed either per g of dry extract (in blue) or per 2.5 g of plant corresponding to one extraction experiment (in red). Interestingly, except for the extracts issued from fresh hawthorn (see next section for more explanations), the TEAC values of all extracts were found to be between 140 and 160 mg eq. Trolox/g dry extract. These results showed that the antioxidant activities were directly related to the overall extracted quantity, whatever the extraction mode and/or plant granulometry (raw, ground). The ranking of the antioxidant activity expressed for 2.5 g of plant (corresponding to one intake) was in good agreement with the ranking of the extraction yields previously discussed, with the best figures of merits obtained for ground material combined with ultrasonic, microwave, or infusion.

### 2.4. Quantification Influence of the Extraction Mode and the Nature/State of the Hawthorn on the Extracted UHPLC Profiles

**Figure 4:**
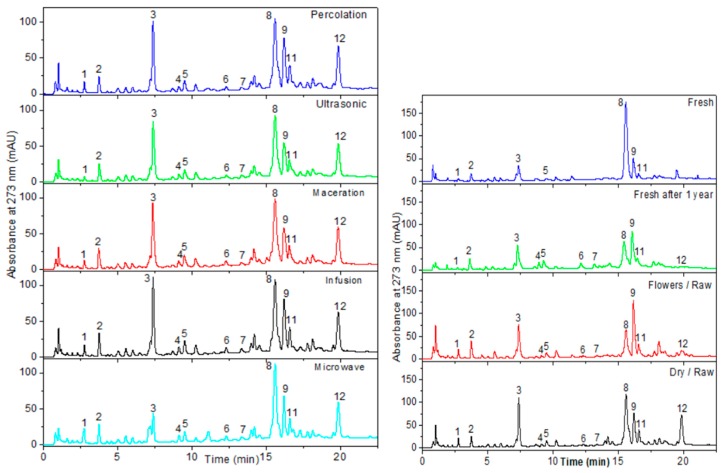
UHPLC profiles of different hawthorn extracts obtained from different extraction modes for 1 mm ground hawthorn flowering tops (**left**) and from the infusion of different parts (flowering tops vs. flowers) or different states (fresh vs. dry) of hawthorn (**right**), as indicated in the figure (see experimental part for the different extraction protocols). Experimental conditions: Luna^®^ Omega polar C18 column (1.6 μm, 100 × 2.1 mm), binary solvent system: water/formic acid (1‰, *v*/*v*) as solvent A and acetonitrile/formic acid (1‰, *v*/*v*) as solvent B. Gradient program: 5% B, then increase of B to 100% in 30 min with a convex increase, flow rate: 0.4 mL.min^−1^, injection volume: 4 μL. Column temperature: 35 °C. UV monitoring at 273 nm. UV–Vis spectra recorded between 200 and 550 nm. Lot number for raw and ground flowering tops materials: 20335. Lot number for flowers: 20334. Fresh flowering tops: harvested in 2017 and extracted one week after (fresh) or one year after (fresh after one year). Peak identification: 1 = cyanidin, 2 = 5-*O*-caffeoylquinic acid, 3 = chlorogenic acid, 4 = procyanidin B2, 5 = epicatechin, 6 = procyanidin C1, 7 = cinnamtannin A2, 8 = vitexin-2-*O*-rhamnoside, 9 = hyperoside, 11 = isoquercetin, 12 = apigenin C-hexoside.

**Figure 5:**
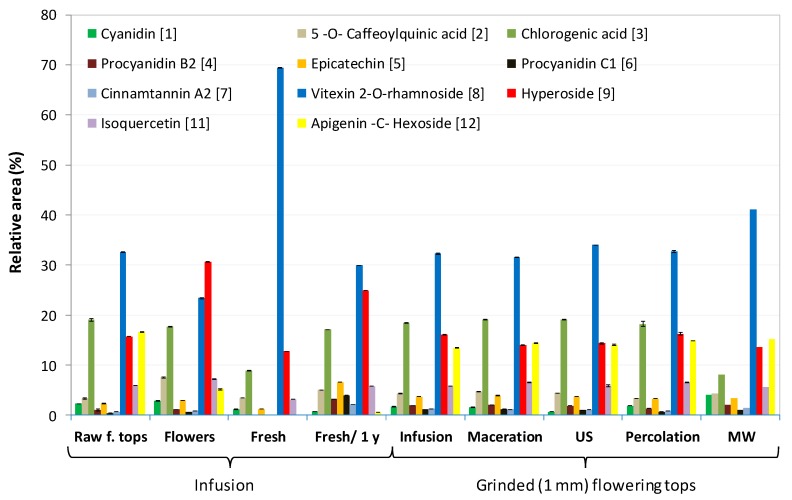
Relative peak area distributions for the main identified chromatographic peaks, according to the extraction mode for ground (1 mm, lot no. 20335) hawthorn (infusion, maceration, US, percolation, MW) or according to the nature of hawthorn by infusion (raw dry flowering tops (lot no. 20335), fresh flowering tops, flowers only (lot no. 20334)). The same experimental conditions as in Figure 4. The relative area was calculated by dividing the peak area of each component by the sum of the peak area of the 12 identified components. Error bars: ±1 standard deviation calculated on *n* = 3 repetitions.

To gain better insight into the differences between the samples obtained from various extraction modes and from diverse natures of hawthorn (flowering tops vs. flowers, dry vs. fresh, raw vs. ground), reversed phase UHPLC and positive mode UHPLC-MS analysis were performed (see Section 3.13 for more details), according to a previously published method used for hawthorn extracts [39]. Figure 4 displays the chromatographic profiles obtained for some of the samples presented in Table S1 (see Figure S5 for the other UHPLC profiles). The corresponding relative peak area distributions are provided in Figure 5 for the 11 main components detected at 273 nm in UHPLC and identified by UHPLC-ESI-MS coupling (positive mode) in the same conditions of elution (see Figure S6 for the peak area distributions issued from all the UHPLC profiles analyzed). Each relative peak area displayed in Figure 5 was calculated by dividing the peak area of each component by the sum of the peak area of the 12 identified components. These values were the average values calculated on three independent extractions. Table 4 contains the list with the names and molar masses of these identified components. Figure S7 gives the chemical structure of all the compounds identified.

Regarding the influence of the extraction mode on the UHPLC profiles, Figure 4 and Figure 5 show that the profiles were very similar for infusion, maceration, US, and percolation modes with a majority (by decreasing order of peak area) of vitexin-2-*O*-rhamnoside (peak 8), chlorogenic acid (peak 3), hyperoside (peak 9), and isoquercetin (peak 11). The two remarkable differences concern the relative content in cyanidin (peak 1), which was significantly lower for the US mode; and a lower relative content in chlorogenic acid for the MW mode. The quantification of vitexin-2-*O*-rhamnoside (peak 8) by external calibration, using a commercially available standard, confirmed that the extraction mode did not significantly change the extracted quantity of this major component (~32–34 mg/g of dry plant for all extraction modes, see Table 2), except for a slightly higher content in MW mode (38.5 mg/g).

The influence of the nature (flowering tops vs. flowers), the state (dry vs. fresh), and the granulometry (raw vs. ground) of the plant on the UHPLC profiles was also investigated. Raw and ground extracts were compared on the same lot (no. 20335) by infusion extraction. Dry flowers (without leaves) were also compared to dry flowering tops using infusion extraction. Extracts obtained by the infusion of fresh flowering tops harvested in April 2017 on Oléron Island (located on the French oceanic west coast, see Figure S1 for more details) were analyzed and compared to the infusion of the same plant after one year drying.

Figure 4 revealed (i) similar profiles between the raw and ground flowering tops, but with a higher content in procyanidins B2 and C1 (peaks 4 and 6) for the ground plant, which is in good agreement with the higher OPC content (3.93 vs. 1.24 mg eq. CY/g of plant); (ii) higher content in hyperoside in dry flowers, but lower contents in apigenin-C-hexoside and vitexin-2-*O*-rhamnoside (the latter being confirmed by external calibration, 22 mg/g of plant) compared to dry flowering tops; and (iii) much higher content (confirmed by external calibration, 51 mg/g of plant) of vitexin-2-*O*-rhamnoside and very low contents in apigenin-C-hexoside and procyanidins (in agreement with low OPC values, 0.41 mg eq. CY/g of plant) in freshly harvested flowering tops. Interestingly, the differences observed in the UHPLC profiles between the fresh and the dry flowering tops (different lots) tended to vanish after one year of drying the ‘fresh’ flowering tops, with increasing contents in epicatechin, hyperoside, apigenin-C-hexoside, and procyanidins for the dry plant.

### 2.5. Influence of Extraction Mode and the Nature/State of the Hawthorn Studied by ESI FT-ICR-MS in Negative Mode

**Figure 6:**
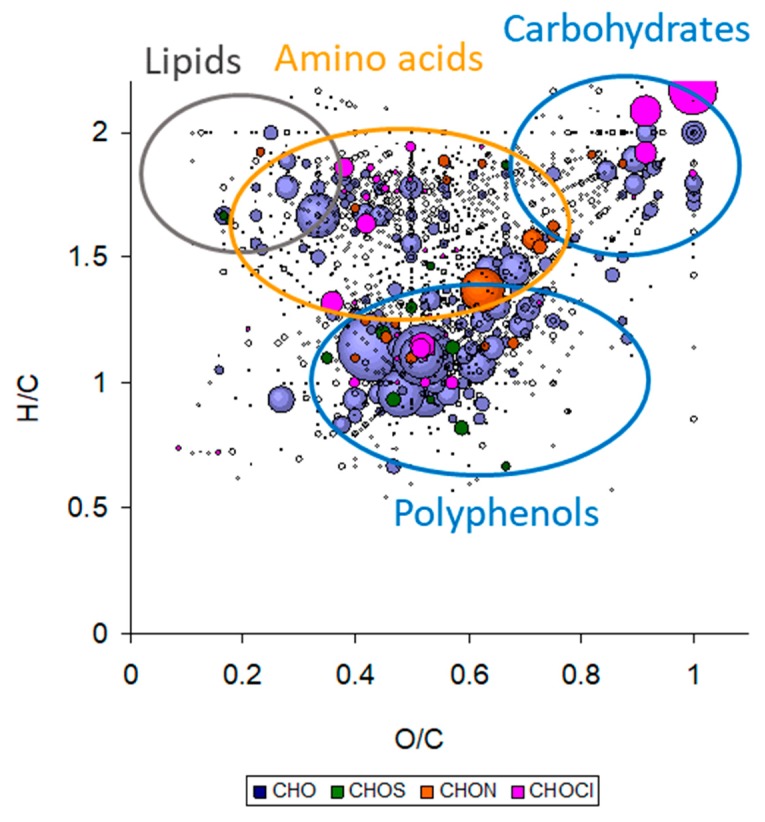
Typical van Krevelen diagram achieved by (−) ESI FT-ICR MS analysis of the hawthorn sample with area of distinguished biochemical compounds. The size of the bubble is relative to the peak intensity.

In this part, the discussion is essentially based on the ground/raw, fresh/dry, and flower/flowering top samples obtained with the previous extraction modes (all samples analyzed by ESI FT-ICR-MS are reported in Table S1). Mass spectra achieved for these samples are given in Figure S8 in duplicate (two independent extractions), with the corresponding global composition description in heteroatom classes and van Krevelen diagram. This latter graph was obtained by plotting the achieved raw formulae according to their H/C and O/C. Depending on the plot location and as illustrated in Figure 6, it is possible to distinguish some areas corresponding to biochemical families such as lipids, polyphenols, amino acids, and carbohydrates [98,99].

**Figure 7:**
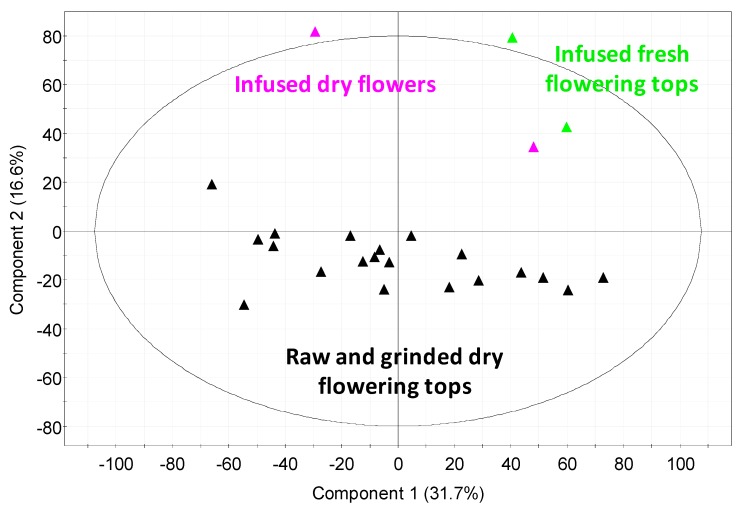
Principal component analysis (PCA) score plot of all mass features from hawthorn extracts measured by (−) ESI FT-ICR MS, in duplicate, obtained by various extraction modes and from different states/nature of the plant: infused dry flower (pink), infused fresh flowering tops (green), and raw and ground dry flowering tops extracted by infusion, maceration, ultrasonic, percolation, and microwave (black).

In this study, most of the samples led to the same global chemical description as illustrated in Figure S8. The achieved van Krevelen diagrams evidenced some biochemical families (Figure 6 and Figure S8). Among the CHO class, some carbohydrates and polyphenols were detected whereas in the CHON one, some amino acids, with possibly some amino sugars, were observed. Regarding the global composition description (Figure S8, pie charts), only samples obtained from the infusion of fresh flowering tops and dry flowers presented a slightly different composition. The achieved global composition description of samples obtained from dry flowering tops was very similar with the CHO, CHOS, CHON, and CHOCl families representing, on average, 63.2 ± 1.4%, 4.7 ± 0.5%, 25.7 ± 1.3%, and 6.4 ± 1.4% of the total assigned features, respectively. For the infusion of fresh flowering tops, the distribution changed to 58.2%, 4.2%, 26.9%, and 10.7% whereas for the samples from the infusion of dry flowers was 54.1%, 2.6%, 37.8%, and 5.5%. The highest number of chlorinated species in the samples from fresh flowering tops can be explained by the harvesting location near the Atlantic Ocean which can favor the presence of NaCl salt and chloride adduct during MS analysis. Regarding the flower samples, a higher number of CHON species, which are likely amino acids and amino sugars, characterizes them. These variations in global composition was confirmed by the PCA of the data obtained by (−) ESI FT-ICR MS analysis (Figure 7). PCA demonstrated higher variability in the composition of the fresh flowering tops and dry flower samples against the dry flowering top samples.

Due to significant composition similarity between the samples, hierarchical clustering analysis (HCA) with heatmap (Figure S9) was carried out to determine the features specific to a sample according to its characteristics (dry/fresh, raw/ground, flower/flowering tops).

**Figure 8:**
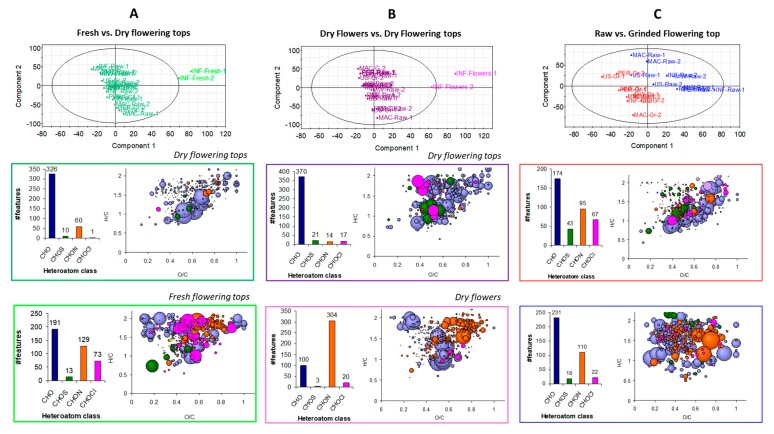
Score plot of partial least squares discriminant analysis (PLS-DA) of hawthorn samples analyzed by (−) ESI FT-ICR MS with (**A**) Fresh vs. dry Flowering tops; (**B**) Dry flowering tops vs. dry flowers, and (**C**) Raw vs. ground samples. Close to 400 assignments specific to each class were extracted and represented on the bar chart according to their heteroatom class and van Krevelen diagram. The bubble size was relative to peak intensity.

First, samples from fresh flowering tops were compared to the dry flowering top samples. Features specifically observed in each class were extracted, represented according to the heteroatom class, and plotted on a van Krevelen diagram (Figure 8A). Moreover, the putative compounds obtained for these extracted features are also reported in Table S4. Features specific to the dry flowering top samples were mainly CHO species and the van Krevelen diagram indicated that they were carbohydrates, and more importantly, polyphenols. Amongst these CHO assignments, one feature at *m*/*z* 353.087809 was intensely detected and can be associated with chlorogenic acid or 5-*O*-caffeoylquinic acid. This observation was confirmed by a more intense peak 3 (chlorogenic acid) in UHPLC-UV analysis (Figure 4) in dry flowering tops compared to fresh flowering tops. The other putative compounds were flavonoids associated to one or more sugar. Regarding the fresh samples, the heteroatom class distribution was more heterogeneous than the dry samples, with more CHON and CHOCl species. This is in agreement with the previous global elemental composition description achieved for this sample. Concerning the former class of CHON compounds, their location on the van Krevelen diagram corresponded to amino acids and amino sugars. The extracted CHO species were lipids, carbohydrates, and polyphenols. Sucrose was found to be a possible component as well as some flavonoids bonded or not to a carbohydrate. Procyanidin A2 was also one matching component of the extracted features.

The same procedure was done with the dry samples, but coming from, on one hand, the flowers (without leaves) and, on the other, the flowering tops (Figure 8B). The features specific to the flowering top samples were mainly CHO compounds. The van Krevelen diagram demonstrated that they were carbohydrates, and more importantly, polyphenols. Some putative features were found, which concern flavonoids linked to a sugar. Concerning the samples from flower infusion, the heteroatom class distribution showed that a significant part of the specific features belonged to the CHON class. These nitrogen-containing species can be regarded as amino acids or amino sugars. In addition, two features are likely to be tryptophan and glutamate. CHO components were also evidenced and are related to lipids and polyphenols. For this latter class of compounds, some matches have been found with flavonoids linked to sugars such as shaftoside (apigenin 6-C-beta-d-glucopyranosyl-8-C-alpha-L-arabinopyranoside) and quercetin pentoside.

A PLS-DA was done between raw and ground flowering top samples. Extracted features relative to the ground samples were plotted on a van Krevelen diagram (Figure 8C). Regarding the CHO class, most of the compounds were polyphenol species. Some putative species were found such as procyanidin A2 and B2. Flavonoid compounds, with catechin and pinnatifida, were also putatively assigned. Some CHON compounds were also present, which corresponded to amino acids, with glutamate putatively assigned. The CHOCl compounds extracted for this class were in the sugar and polyphenol areas of the van Krevelen diagram. The extracted features of the raw sample class were mainly CHO and CHON compounds. The CHO species were not related to particular biochemical compound families. A few components were sugars and polyphenols, but low-intensity detected. A broader range of amino acids was achieved from this PLS-DA class. Such observations are in agreement with those obtained in UHPLC-UV with MS coupling (Figure 4), which demonstrated higher OPC in the ground samples than in the raw ones.

The (−) ESI FT-ICR MS analysis enabled us to achieve a more extensive description of the samples. Despite strong similarities between the samples, it has been possible to extract features specific to some characteristics of sample preparation such as the granulometry or the plant organ. Thus, this approach ensured the confirmation of previous observations obtained in UHPLC-UV with MS coupling and to extend it to other putatively assigned species. The additional studies highlighted composition differences between the dry flowering top samples vs. the fresh ones and the dry flowers. The achieved results were consistent with the global composition, with a significant amount of CHON species for the flower samples and, to a lesser extent, the fresh ones. In addition to all the CHO species detected by (−) ESI FT-ICR MS, the information obtained on the nitrogen-containing compounds was complementary to that obtained with the previous analytical method. This demonstrates the complementarity of FT-ICR MS for the exhaustive characterization of hawthorn samples.

### 2.6. Optimization of the Homemade Infusion Protocol and Characterization of the Plant Granulometry

One of the main objective of this work was to develop a straightforward extraction protocol that could be accurately reproduced at home, maximize the extraction of TPC/TFC/OPC within a minimum of time, and kept as simple as possible (either in terms of manipulations or in the material required for the extraction). As we wanted to stick to real-life conditions/applications, the following experimental parameters were investigated to optimize the home-made protocol: (i) the size of the container (cup, mug, or bowl, see Figure S10 for the dimensions and shapes) and therefore, the volume of introduced water (125, 250, and 405 mL, respectively); (ii) the presence or absence of magnetic stirring; (iii) the plant granulometry was varied by using different affordable coffee grinders; and (iv) the use (or not) of a tea bag. Since infusion mode has been found to be one of the best compromises as far as ground materials are used, the optimized protocol was based on infusion. Temperature decrease profiles (see Figure S11 in Supplementary Materials) can be correctly fitted (for natural convection experiments only) using a relatively simple model that considers the nature and geometry of the recipient. This model was based on the numerical resolution of the system of differential equations corresponding to the instantaneous material and thermal balances using a Mathcad script courteously provided by Condoret [100]. For example, 58% of the heat is evacuated via the vertical wall, and 32% by evaporation for the mug. On the other hand, when the horizontal surface is greatly increased, the evaporation contributes to more than 65% of the energy loss.

**Figure 9:**
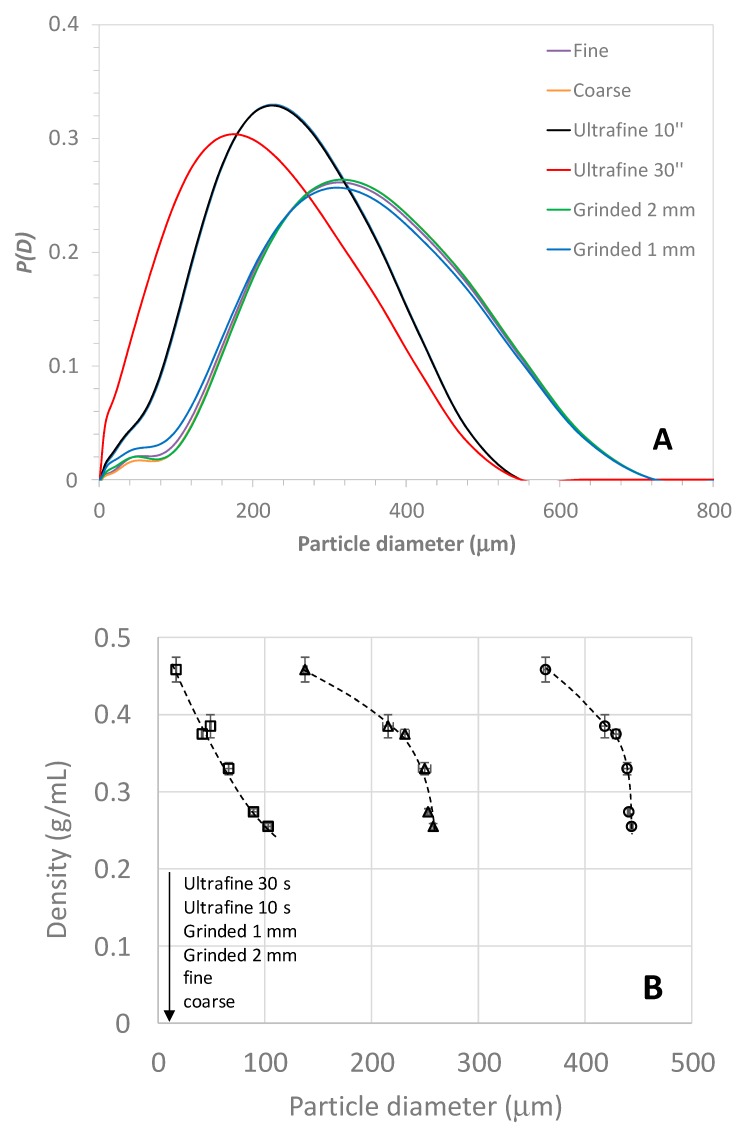
Relative size distributions of the raw and ground hawthorn materials obtained by laser granulometry in dry mode (**A**) and variation of the density of hawthorn materials as a function of the particle diameter (**B**). *D_10_* (☐), *D_50_* (Δ), and *D_90_* (Ο) with *D_x_* being the 10^th^ decile of the distribution. See Section 3.2 for more details on the experimental conditions. Pictures of the hawthorn materials taken on millimeter paper are provided in the Supplementary Materials (see Figure S12).

As plant granulometry is the main factor influencing the kinetics and yield of extraction, it was crucial to find a simple way to grind the plants in a reproducible and affordable manner. Two electric coffee grinders were used and compared. A Delonghi (model KG79) grinder, equipped with a burr-grinding wheel, was used at the two extreme positions, namely coarse and fine positions, to obtain different granulometry. For finer grinding, a Bosch electric grinder (Model MK6003) equipped with a fast rotating stainless steel chopping blade was used with two different grinding times (10 s and 30 s by shaking the grinder simultaneously). For comparison, a grinding laboratory equipment (Ika, Model MF10 basic) was also used with two different grid sizes (1 mm and 2 mm). Figure S12 shows the pictures of the hawthorn material on graph paper, before (raw) and after grinding. The density of the ground material was measured using a graduated test tube (see Section 3.2 for more details), which is a very simple way to estimate the ground plant density. This experimental parameter could be useful to optimize the grinding protocol, since it is related to the size distribution for polydisperse and non-spherical samples. Clearly, as seen in Figure 8A, the density tended to increase with lower granulometry, which was in the order of coarse < fine < ground 2 mm < ground 1 mm < ultrafine 10′’ < ultrafine 30′’. To obtain better quantitative data, the size distribution of the plant particle was determined by laser granulometry in the dry phase (see Section 3.2) and the corresponding distributions are presented in Figure 9A (see also Figure S12 for the repetitions). Volume size distributions (given in diameter) were very broad with typical sizes ranging between 10–20 µm for the smaller particles, up to 500–700 µm for the largest. Most of the distributions displayed a bimodal curve with a main mode between 180–300 µm, and a much less intense mode (or shoulder) at smaller sizes between 10 and 80 µm. The ‘fine’ distribution was close to the 2 mm ground material while the ultra-fine 10′’ and 30” presented much lower granulometry. Figure 9 represents the correlation between the ground dry plant density and the particle diameters taken at different deciles of the distribution (*D_10_* is the first decile, *D_50_* is the median value, and *D_90_* is the ninth decile). Interestingly, the most regular correlation between the plant density and the particle diameter was obtained with *D_10_*. Such correlation could be useful to obtain a rough estimation of the hawthorn granulometry from a simple determination of the dry plant density before or after grinding.

Regarding the choice of the recipient, the global extraction yield was found to be higher (about 10–15% more) for the bowl and mug than the cup, suggesting that a higher volume of water can extract a higher amount of compounds (Table 5). It is worth noting that this effect has nothing to do with the differences in the temperature profiles according to the recipient (see Figure S11), which is in the order of cup < bowl < mug (from lower to higher temperature at 10 min extraction time). TPC, TFC, and OPC values were in the order of cup < mug < bowl, with more than a 50% increase for the bowl when compared to the cup. TPC, TFC, and OPC values obtained for the bowl using 250 mL of water were found in the same range as those for the previously used three-necked flask. Using a 1 mm or 2 mm grinder did not significantly change the extraction results.

The use of a Celia^®^ bag to avoid plant particles dispersing into the extracted solution should be avoided for practical reasons since it decreased both the extraction yield and the TPC, TFC, and OPC contents (Table 5). This effect can be explained either by a lower diffusion of the extracted components from the inside to the outside part of the bag, or by a retention of a significant part of the extracted soluble compounds onto the surface of the paper bag. As a matter of comparison, an infusion in a mug with a Celia bag is similar to an infusion in a cup without a Celia bag. When the granulometry of the plant is too fine (ultrafine 10′’), the extraction yields of all components drop to values even lower than for raw materials, suggesting that there is a critical particle size value under which the pores of the bag are clogged.

Finally, the simplest and optimized way to perform an infusion at home, without using a tea bag, is to use a ‘French-press’ coffee maker that is able to receive at least 250 mL of water (see Figure S2). This allows avoiding the use of a filter bag, the plant being freely floating in the recipient during the infusion, while the piston permits pushing the residual solid parts of the plant to the bottom of the recipient at the end of the extraction, before serving. It is worth noting that the granulometry of the ground plant between fine (Delonghi grinder), coarse (Delonghi grinder), and ultrafine 10′’ (Bosch grinder) did not significantly impact the extraction yield and the quantities of TPC, TFC, and OPC that were extracted (see Table 5).

The effect of stirring was also investigated by comparing the same extraction with and without magnetic stirring. As far as ground materials are concerned (fine position or smaller sizes), the effect of this parameter was negligible: a short manual stirring of the Bodum^®^ pot at the beginning and at the end of the extraction was enough to obtain a quantitative extraction of the water-soluble components.

### 2.7. Variability between Hawthorn Lots

The global extraction yield was compared on four different lots of dry flowering tops. Table 5 shows that the extraction yield was almost the same (about 22%, non-significant effect of the lot number by one-way ANOVA at 0.95 confidence level) for all lots. In contrast, significant variations of the TPC (20–34 mg eq. GA), TFC (2.9–3.7 mg eq. Q), and OPC (1.2–1.8 mg eq. CY), expressed per g of dry plant, were observed and confirmed by one-way ANOVA at the 0.95 confidence level. Therefore, the total amount of dry extract was almost constant, but the repartition between the different classes of components could differ from one lot to the other. The change in composition was already observed on a given lot between fresh and dry flowering tops (see Section 3.4). We can conclude that, not only the lot, but also the maturation of the plant can affect the repartition between the different classes of components. Interestingly, the maximum quantities of TFC, TPC, OPC (44, 4.1, and 4.2 mg, respectively) were obtained from the flowering tops harvested on Oléron Island after one year of drying. Figures S14 and S15 display the chromatograms and the peak area repartition, respectively, for the different lots. Similar compounds were detected on the UHPLC profiles, but some differences were mainly observed on the relative proportion of the different components. We noticed that hyperoside was more abundant in flowers than in the flowering tops.

## 3. Materials and Methods

### 3.1. Chemicals

Different lots of dry hawthorn flowering tops (20335, 55849, CB58120, H18001534, 1221478, R78925) or dry flowers (20334) raw materials (*Crataegus oxyacantha*, origin France) were purchased from France Herboristerie (Noidans-Lès-Vesoul, France). Fresh *Crataegus oxyacantha* flowering tops were harvested on April 24, 2017 (see Figure S1 for the exact localization and picture of the fresh flowering tops) on a wild isolated tree on Oléron Island (France). Folin-Ciocalteu reagent, sodium carbonate (Na_2_CO_3_), aluminum chloride hexahydrate (AlCl_3_.6H_2_O), (±)-6-hydroxy-2,5,7,8-tetramethylchromane-2-carboxylic acid (Trolox), 1,1-diphenyl-2-picrylhydrazyl (DPPH), methanol (CH_3_OH), hydrochloric acid (HCl), *n*-butanol (CH_3_-(CH_2_)_3_-OH), ammonium iron(III) sulfate dodecahydrate (NH_4_Fe(SO_4_)_2_.12 H_2_O), gallic acid (GA), quercetin (Q), and cyanidin chloride (CY) were purchased from Merck (Saint-Quentin Fallavier, France). Crataegus spp. extract standard (R1) was purchased from HWI Group (Rülzheim, Germany) standardized at 29 mg of vitexin 2-*O*-rhamnoside per g of extract. Procyanidin A2, B2, and C1, epicatechin, cinnamtannin A2, and isoquercetin were purchased from Phytolab (Vestenbergsgreuth, Germany). Ultrapure water was obtained using a MilliQ system from Millipore (Molsheim, France). Celia^®^ bags (size L) were purchased from a Casino local supermarket (Montpellier, France). Standardized plant extract (EPS) Phytoprevent^®^ (standardized fresh hawthorn fluid extract, standardized at 7.5–12.5 mg flavonoids as equivalent quercetin in 5 mL) containing 900 mg dry flowering tops extract/5 mL glycerol was purchased from Pilege (Orée d’Anjou, France). Crataegisan^®^ Bioforce ethanolic plant extract (46–54% EtOH) containing 690 mg tincture of fresh hawthorn fruits in 2.25 mL, standardized at 12.7 mg polyphenol, and 6.4 mg oligomeric procyanidins was purchased from Vogel (Colmar, France). WS1442^®^ crataegutt novo 450 tablets (also named Cardiplant^®^ 450 or Cardio Max WS 1442^®^) containing 450 mg dry plant extract each and standardized at 78–90.6 mg oligomeric procyanidins as equivalent epicatechin (17.3–20.1% in dry extract) were purchased from Schwabe Pharmaceuticals (Karlsruhe, Germany). Faros ^®^ 300 LI 132 tablets containing 300 mg dry plant extract each and standardized at 6.6 mg flavonoids as equivalent hyperoside (2.25% in dry extract) were purchased from LichtwerPharma (Berlin, Germany).

### 3.2. Ground Hawthorn, Density, and Granulometry

Dry hawthorn plants were ground using three different grinders. ‘Coarse’ and ‘fine’ hawthorn materials were obtained by grinding 2 g of raw material using Delonghi (Model KG79, Trevise, Italy) grinder at the positions ‘coarse’ and ‘fine’, respectively. ‘Ultrafine 10 s or 30s’ hawthorn materials were obtained by grinding 2 g of raw material using the Bosch grinder (Model MKM6003, Munich, Germany) at different manual shaking times (10 s and 30 s) as indicated in the text. The ‘1 mm’ and ‘2 mm’ hawthorn materials were obtained by grinding the required amount of raw material on a laboratory Ika grinder (Ika-Werke GmbH, Model MF10 basic, Staufen, Germany). The density of each hawthorn material was simply determined by measuring the volume occupied by 2 g hawthorn material in a 10 mL (or 25 mL) graduated test tube (*n* = 3 determinations). Distribution in size of each hawthorn material was determined by a dry laser Malvern granulometer (Malvern Panalytical, Royston, United Kingdom).

### 3.3. Infusion Extraction

Infusion extraction was performed using a 500 mL three-necked flask equipped with an olive magnetic stirrer (Figure S2A). A total 2.5 g of dry plant was placed in it and 250 mL of boiled ultrapure water was added. Four different mixing speeds were tested, namely 250 rpm, 500 rpm, 750 rpm, and 1000 rpm. The decrease in temperature was measured upon time using a temperature sensor (Ebro EBI20-IF, Ingolstadt, Germany). Three extraction times were investigated, namely 5 min, 10 min, and 30 min. After filtration of the plant residues using Whatman filter paper placed on a Büchner funnel and a vacuum pump (KNF Model N820FT.18, Freiburg, Germany), the extract was concentrated using a rotary evaporator (until 10 mL volume) and finally freeze-dried (Cryotec Model CRIOS-80, Saint-Gély-du-Fesc, France). Lyophilized dry extracts were stored at 4 °C. Each extraction experiment was carried out in triplicate.

### 3.4. Maceration Extraction

Maceration extraction was performed using a 500 mL three-necked flask equipped with an olive magnetic stirrer, an oil bath, and a heating magnetic stirrer with a digital thermo-regulator (Fisher Scientific Model FB15002, Illkirch, France) (Figure S2B). A total 2.5 g of dry plant was placed in a container and 250 mL of water was added. Four different temperatures were tested, namely 20 °C, 40 °C, 60 °C, and 80 °C. The mixture was stirred at 500 rpm. Three extraction times were investigated, namely 5 min, 10 min, and 30 min. After filtration, the extract was concentrated and finally freeze-dried (as described in Section 2.3). Each extraction experiment was carried out in triplicate.

### 3.5. Ultrasound-Assisted Extraction

Ultrasound-assisted (US) extraction was conducted with an ultrasonic homogenizer (UIP 1000 hdT, 1kW, Hielscher Ultrasonics GmbH, Germany). Experiments have been performed in a double jacket reactor of 1 L volume (Figure S2D) connected with a mechanical stirrer (IKA RSC classic, Germany) and a temperature sensor. Temperature was maintained at constant with a cooling system connected to the double-jacket reactor. A total 2.5 g of dry plant was placed in the double jacket reactor and 250 mL of water was added. Three different temperatures were tested, namely 20 °C, 40 °C, and 60 °C. The mixture was mechanically stirred at 250 rpm. Three extraction times were investigated, namely 5 min, 10 min, and 30 min. After filtration, the extract was concentrated and finally freeze-dried (see Section 2.3). Each extraction experiment was carried out in triplicate.

### 3.6. Microwave-Assisted Extraction

Microwave-assisted (MW) extraction was performed on a monomode microwave apparatus using a closed-vessel system (NEOS-GR, Milestone Srl, Italy) (Figure S2E). A total of 2.5 g of dry plant was placed in a 500 mL flask containing 250 mL of water. The flask was then placed in the MW oven with a 300 W power. Under these conditions, the temperature reached 95 °C in 10 min. No stirring was applied. Three extraction times were investigated, namely 5 min (78 °C), 10 min (95 °C), and 30 min (97 °C). After filtration, the extracts were concentrated and finally freeze-dried (see Section 2.3). Each extraction experiment was carried out in triplicate.

### 3.7. Percolation Extraction

Percolation extraction was performed using a coffee percolator KRUPS equipment (Model, city, Germany) (Figure S2C). A total of 2.5 g of dry plant was placed in a filter and 250 mL of water was used. The temperature reached 100 °C after a few seconds. No stirring was applied. Two extraction times were investigated, namely 5 min and 10 min. A total of 250 mL of water was percolated in 5 min. For the 10 min percolation extraction, the extracted solution obtained after 5 min percolation was passed again in the percolator for another 5 min. After filtration, the extract was concentrated and finally freeze-dried (see Section 2.3). Each extraction experiment was carried out in triplicate.

### 3.8. Optimized Infusion Extraction

A sample of 2.5 g ground material (see Section 3.2) was infused in 250 mL boiling water using a ‘French press’ Bodum^®^ (Bistro model, Triengen, Switzerland). After addition of the boiling water, a good initial mixing of the plant in water was ensured by manually rotating the recipient (with ground plant, magnetic stirring—which is generally not available at home—was however not required to obtain optimal extraction). After 10 min, the herbal tea solution was filtered first with the Bodum^®^ cover to remove the largest particles, then with Whatman filter paper to remove any residual solid plant. Finally, the herbal tea solution was concentrated and freeze-dried to obtain the dry extract.

### 3.9. Kinetic Monitoring

The kinetic of extraction was monitored by UV absorbance at 198 nm (Perkin-Elmer Model Lambda 20, Wellesley, MA, USA) using UV quartz cells of 1 mL (Hellma GmbH, Müllheim, Germany). A sample of 100 μL solution was taken and added to 4 mL (or 8 mL if the absorbance values were above 1.7) water. The resulting solution was shortly vortexed before UV measurement. The same volume of fresh water (100 µL) was added in the reactor (three-necked flask) to keep the total volume constant. A zero absorbance value was set using 100 μL of water instead of herbal tea solution.

### 3.10. Total Polyphenols Content (TPC)

The total polyphenols content (TPC) in hawthorn extracts was estimated using the Folin-Ciocalteu’s reagent as described by Singleton and Rossi [101]. A 100 μL solution prepared by mixing hawthorn extract (100 μL of 20 mg/mL in water) with 1 mL water was added to 200 μL Folin-Ciocalteu reagent and 2 mL water. After 3 min, 1 mL of 20% sodium carbonate (20 g/100 mL water) was added. After vortex-mixing for 2 min, followed by incubation at room temperature and in darkness for 90 min, the resulting solution was centrifuged at 8000 rpm for 3 min (Sigma Model 302K, Osterode am Harz, Germany) and the absorbance at 760 nm was measured using the same equipment as in Section 3.9. Gallic acid (0–250 mg/L) was used for the standard calibration curve. The results were expressed as mg GA equivalent per gram of dry plant, and calculated as the mean value ±1 SD (*n* = 3). A zero absorbance value was set using 100 μL of water instead of the herbal tea solution.

### 3.11. Total Flavonoids Content (TFC)

The total flavonoids content (TFC) in hawthorn extracts was estimated by the aluminum chloride method according to Lamaison and Carnet [102] where 200 μL of hawthorn extract solution (20 mg/mL in water) was first added to 200 μL water and 600 μL methanol. Then, 200 μL of the resulting solution were added to 800 μL methanol and 1 mL of 2% AlCl_3_, 6H_2_O methanolic solution (2 g/100 mL in methanol). After vortex-mixing for 2 min, followed by incubation at room temperature and in darkness for 15 min, the absorbance at 430 nm was measured using the same equipment as in Section 3.9. Quercetin (0–35 mg/L) was used for the standard calibration curve. The results were expressed as mg of equivalent Quercetin per gram of dry plant, and calculated as the mean value ±1 SD (*n* = 3). A zero absorbance value was set using 200 μL water instead of the herbal tea solution.

### 3.12. Total Proanthocyanidin Oligomers Content (OPC)

The total proanthocyanidin oligomers content (OPC) in hawthorn extracts was estimated using the HCl/*n*-butanol assay of Porter et al. [103]. A 200 μL hawthorn extract solution (20 mg/mL in water) was first added to 200 μL water and 600 μL methanol. Then, 250 μL of the resulting solution was added to 3 mL of a 95% solution of *n*-butanol/HCl (95:5 *v*/*v*) and 100 μL of a 2% solution of NH_4_Fe(SO_4_)_2_, 12H_2_O (2 g/100 mL in HCl 2 M). After vortex-mixing for 2 min, followed by incubation at 95 °C in an oil bath for 40 min and cooling at room temperature, the absorbance at 550 nm was measured using the same equipment as in Section 3.9. Cyanidin chloride (0–30 mg/L) was used for the standard calibration curve. The results were expressed as mg CY equivalent per gram of dry plant, and calculated as mean value ± SD (*n* = 3). Zero absorbance value was set using 200 μL water instead of herbal tea solution.

### 3.13. UHPLC and UHPLC-ESI-MS Analysis

All samples analyzed are presented in Table S1. A 20 mg hawthorn dry extract was dissolved in 1 mL MilliQ water, and finally strongly vortexed for 2 min. The resulting solution was diluted five times with MilliQ water, vortexed again for 2 min, and analyzed by UHPLC-DAD and UHPLC-ESI-MS.

The UHPLC-DAD system consisted of a Thermo Scientific™ Dionex™ UltiMate™ 3000 BioRS equipped with a WPS-3000TBRS auto sampler, and a TCC-3000RS column compartment set at 35 °C (Thermofisher Scientific, Waltham MA, USA). The system was operated using Chromeleon 7 software. A Luna^®^ Omega polar C18 column (1.6 μm, 100 × 2.1 mm) combined with a security guard ultra-cartridge was used (Phenomenex Inc., Torrance CA, USA). A binary solvent system was used, consisting of water/formic acid (1‰, *v*/*v*) as solvent A and acetonitrile/formic acid (1‰, *v*/*v*) as solvent B. The gradient program started with 5% B, then B was increased to 100% in 30 min with a convex increase (curve 5 in Chromeleon 7). The flow rate of the mobile phase was 0.4 mL.min^−1^, and the injection volume was 4 μL. The peaks were monitored at 273 nm. The UV–Vis spectra of the different compounds were recorded between 200 and 550 nm using the diode array detector (DAD).

UHPLC-ESI-MS analysis was performed using a Synapt G2-S (Waters Corp., Milford MA, USA) equipped with ESI. The UHPLC column, injection volume, flow rate and gradient program were the same as for UHPLC-DAD. Positive mode was used according to [39]. The capillary voltage was set to 3 kV, the cone voltage was set to 30 V, and the extractor voltage was set to 3 V. The source temperature was 100 °C and the desolvation temperature was 450 °C. MS spectra were obtained by scanning ions between *m*/*z* = 100 and *m*/*z* = 1500. The system was operated using MassLynx 4.1 software.

### 3.14. (−) ESI FT-ICR-MS Analysis

All of the analyzed samples are given in Table S1 and were studied in duplicate. Extraction of the achieved material, depending on the diverse extraction processes, was carried out by 2 mL methanol addition in vial and 5 min ultrasonic bath at room temperature. The methanolic extracts (from light to dark yellow), constituting the stock solutions, were recovered and put in 2.5 mL vials for 2 min centrifugation at 14,000 rpm for 2 min. These solutions were then diluted 100 times in methanol. Standardized EPS Phytoprevent^®^ and Crataegisan^®^ Bioforce extracts were diluted to 0.5% in methanol.

Sample analysis was performed with a 12 T FT-ICR mass spectrometer Solarix (Bruker Daltonics) and the parameters were optimized via software FTMS-Control V2.2.0 (Bruker Daltonics). Prior to acquisition, the mass spectrometer was externally calibrated with arginine clusters (10 mg/L in methanol). Hawthorn methanolic solutions were infused with a flow rate of 2 µL/min in the ESI source (Apollo II, Bruker Daltonics) used in negative-ion mode with a capillary voltage set at 3.6 kV. The temperature and the flow rate of the drying gas were kept at 180 °C and 4 L/min, respectively, and the pressure of the nebulizer gas was 2.2 bar. Mass spectra resulted from the accumulation of 300 scans over a *m*/*z* 122–100 range, and with a 4 megaword time-domain.

The achieved mass spectra were processed in Data Analysis 5.0 (Bruker Daltonics). An internal calibration with a list of well-known C_x_H_y_O_z_ (fatty acids and sugars) anions was performed with mass accuracy values lower than 200 ppb. Peak lists were generated at signal-to-noise ratio ≥4 and exported. The algorithm developed by Kanawati et al. was applied to remove signals related to satellite and magnetron peaks [104]. Apart from the standard extracts, the samples were analyzed in duplicate, therefore, only features observed in both replicates were kept. The filtered mass lists of the different samples were finally aligned into a matrix based on their *m*/*z* values with a 0.5 ppm tolerance. The achieved matrix was processed for assignment in an in-house software, Netcalc [105], with an annotation tolerance of 0.2 ppm. Eventually, the CHO, CHOS, CHON, and CHOCl compound families were assigned.

Perseus software was used to perform hierarchical cluster analysis (HCA) and to generate heatmap from the data achieved by (−) ESI FT-ICR MS analysis of the samples. Close to 400 specific features were retrieved depending on the extraction process, plant status, or physical shape (raw or ground). These were then represented in a histogram according to their heteroatom class and the van Krevelen diagram. Principal component analysis (PCA) and partial least squares-discriminant analysis (PLS-DA) were carried out by SIMCA-P 9.0 software.

Some of the achieved raw formulae were putatively assigned with compounds previously identified in the fruit, leaves, and flowers of *Crataegus* [39,40,106]. Thus, 56 compounds were assigned and referenced, with their exact mass in the [M − H]^−^ form in Table S3.

### 3.15. Anti-Oxidant Activities

The DPPH (common abbreviation for 2,2-diphenyl-1-picrylhydrazyl) scavenging capacity of the hawthorn extracts was measured using Trolox as a standard [107,108]. A 50 µL of 0.5 mM methanolic DPPH solution was added to 50 µL of extracts (or Trolox) in a microplate and the adsorbance was read at 520 nm every 5 min over a period of 60 min. All experiments were carried out in triplicate and for three independent extractions. Final results were expressed in milligrams of Trolox equivalent (TE) per gram of hawthorn extract.

## 4. Conclusions

From this thorough study regarding hawthorn extraction in water, we can conclude that home-made preparations using infusion with simple commercially available equipment and protocol can afford a daily intake of TPC, TFC, and OPC similar to the recommended dose from standardized plant extracts. The optimal home-made conditions are: (i) the grinding of 2.5 g of hawthorn flowering tops using a basic commercially available grinder just before the infusion (granulometry < 1 mm); (ii) pouring 250–400 mL of boiling water onto the ground plant in a French-press coffee maker (no infusion bag, no stirring required!); (iii) waiting for at least 3 min of infusion; and (iv) pressing the French-press filter before serving. The overall cost for one month of daily hawthorn intake (one infusion per day) is about 2.2 euros to cover the cost of the hawthorn material and if the cost of the equipment, which can be reused, is considered. This is about 10 times lower than the cost of the standardized plant extract and this cost can be even further reduced if people harvest hawthorn by themselves.

Grinding the plant was found to be the best way to increase the kinetics of extraction and the overall yield of extraction; but it is advisory to grind (granulometry < 1 mm) just before use to avoid any undesirable oxidation of the plant. If the plant is ground, infusion remains the simplest way to extract bioactive components from hawthorn plants, and the other extraction modes (ultrasonic, maceration, microwaves, and percolation) did not significantly improve the extraction yield. The UHPLC profiles were also very similar from one extraction mode to the other. As far as the plant is ground, the automatic stirring of the infusion is not required and simple manual stirring at the beginning and the end of the extraction is enough to obtain optimal extraction. Similarly, it is not necessary to wait more than 3 min for the infusion of ground hawthorn; however, a 10 min infusion can be a good option to reach drinkable temperature (i.e., 60 °C or lower temperatures), without adding fresh water to the infusion to decrease the temperature. The use of a tea bag is not recommended since it tends to slow down the extraction process/diffusion and decrease the yields of extraction, either due to pore clogging of the filter constituting the tea bag (this is especially critical for fine/ultrafine granulometry) and/or due to solutes adsorption on the filter. Overall extraction yield for the optimized protocol was about 22% (in wt of the initial dry plant) among which 8% (in wt of the initial dry plant) were non-UV absorbing components. Among the different volumes of water tested (cup = 125 mL, mug = 250 L, and bowl = 405 mL), the higher volume (bowl) was the best to optimize the yields of extraction (10%–15% increase in global yield compared to the cup and up to 50% for TPC, TFC, and OPC contents). The global extraction yield remained unchanged for all five hawthorn lots tested; but the repartition between TPC, TFC, and OPC may vary from one lot to the other.

Regarding the quantitative and qualitative differences according to the nature of the hawthorn plant, we can conclude that: (i) similar UHPLC profiles were obtained between raw and ground flowering tops, but with a higher content in procyanidins B2 and C1 for ground plant, which is in good agreement with the higher OPC content (3.93 vs. 1.24 mg eq. CY/g of plant); (ii) dry flowers (without leaves) had a higher content in hyperoside, but lower contents in apigenin-C-hexoside and vitexin-2-*O*-rhamnoside when compared to dry flowering tops; and (iii) a much higher content of vitexin-2-*O*-rhamnoside and lower contents in apigenin-C-hexoside, procyanidins and chlorogenic acid were obtained in freshly harvested flowering tops compared to dry raw flowering tops. Interestingly, the differences observed in the UHPLC profiles between the fresh and the dry flowering tops (different lots) tended to vanish after one year of drying the ‘fresh’ flowering tops, with increasing contents in epicatechin, hyperoside, apigenin-C-hexoside, procyanidins, and OPC in general once the plant is dried. From (−) ESI FT-ICR MS analysis, additional conclusions are that: (i) there is a higher variability in chemical composition in fresh flowering top samples compared to dry flowering tops, and in dry flowers (without leaves) compared to dry flowering tops; (ii) fresh flowering tops contain higher contents in amino acids, amino sugars, lipids, carbohydrates, and some flavonoids bonded or not to a carbohydrate (such as procyanidin A2); (iii) dry flowers contain more amino acids (e.g., tryptophan and glutamate) or amino sugars and flavonoids linked to sugars such as shaftoside (apigenin 6-C-beta-d-glucopyranosyl-8-C-alpha-L-arabinopyranoside) and quercetin pentoside as compared to the dry flowering tops.

We believe that the home-made optimized protocol described in this work, which is based on a simple water-based infusion, is of very general use for those who are interested in medicinal plants. It presents the advantages of being very simple, fast, affordable, repeatable, and optimized. These features are some of the key points to address if we want to promote herbal medicine to favor its acceptance in modern Western integrative medicine [109] and to meet the increasing societal demand in that field [110].

## Figures and Tables

**Table 1:** List of the experimental parameters and their modalities investigated in this work for each extraction mode.

	Extraction Mode				
Parameter of Extraction	Infusion	Maceration	Ultrasonic	Microwave	Percolation
Temperature (°C)	Decreasing from 90 upon time	20, 40, 60, 80	20, 40, 60	96 (300 W)	100
Stirring speed (rpm)	250, 500, 750, 1000 (Magnetic stirring)	500 (magnetic stirring)	250 (mechanical stirring)	No	No
Extraction time (min)	5, 10, 30	5, 10, 30	5, 10, 30	5, 10, 30	5, 10
Plant state	Fresh, fresh after 1 year, dry	fresh, dry	dry	dry	dry
Plant granulometry	Raw, ground (1 mm, 2 mm, coarse, fine, ultrafine 10″ and 30″)	raw, ground 1 mm	raw, ground 1 mm	raw, ground 1 mm	raw, ground 1 mm

**Table 2:** Physicochemical characteristics of the flowering top hawthorn extracts depending on the extraction mode, the extraction time, and the plant granulometry. In all cases, 2.5 g of hawthorn material in 250 mL water was used. For kinetic UV monitoring, 100 µL of solution was taken and added to 4 mL water before UV measurement, except for ^a^, where 100 µL was added to 8 mL of water to avoid spectrometer saturation (values reported in the table are multiplied by a factor of 2 for better comparison). ^b^: ±1 standard deviation calculated on *n* = 3 repetitions. ^c^: in mg eq. GA/g dry plant, ±1 standard deviation calculated on *n* = 3 repetitions. ^d^: in mg eq. Q/g dry plant, ±1 standard deviation calculated on *n* = 3 repetitions. ^e^: in mg eq. CY/g dry plant, ± 1 standard deviation calculated on *n* = 3 repetitions. ^f^: in mg/g dry plant, ±1 standard deviation calculated on *n* = 3 repetitions. Lot number for raw and ground flowering top materials: 20335. Lot number for flowers only: 20334. Fresh flowering tops: harvested in 2017.

Plant Nature & Granulometry	Extraction Mode	Experimental Conditions	*t*_70%_ (min)	A(*t*) at 30 min	10 min Extraction Time					30 min Extraction Time			
					Extraction Yield (%) ^b^	TPC ^c^	TFC ^d^	OPC ^e^	Vitexin *O*-rhamnoside ^f^	Extraction Yield (%) ^b^	TPC ^c^	TFC ^d^	OPC ^e^
Flowering tops (Raw)	Infusion	250 rpm	9	1.41	-	-	-	-	-	14.29 ± 0.56	-	-	-
		500 rpm	8	1.51	15.64 ± 0.83	18.90 ± 1.72	2.33 ± 0.19	1.24 ± 0.10	33.56 ± 1.30	16.14 ± 0.45	18.78 ± 0.68	2.47 ± 0.07	1.70 ± 0.16
		750 rpm	6	1.47	-	-	-	-	-	15.03 ± 0.71	-	-	-
		1000 rpm	4	1.58	-	-	-	-	-	16.02 ± 0.89	-	-	-
	Maceration (at 500 rpm)	20 °C	12	0.66	-	-	-	-	-	9.75 ± 0.56	-	-	-
		40 °C	12	0.80	-	-	-	-	-	11.87 ± 0.75	-	-	-
		60 °C	13	1.17	12.02 ± 0.81	12.57 ± 0.74	1.47 ± 0.15	1.06 ± 0.02	30.91 ± 2.13	14.18 ± 0.67	14.60 ± 1.67	1.86 ±0.18	1.10 ± 0.07
		80 °C	13	1.76	-	-	-	-	-	16.19 ± 1.07	-	-	-
	US (at 250 rpm)	20 °C	10	1.38	-	-	-	-	-	13.65 ± 1.22	-	-	-
		40 °C	8	1.63	-	-	-	-	-	14.48 ± 1.19	-	-	-
		60 °C	8	2.14 ^a^	17.21 ± 0.53	21.17 ± 2.57	2.46 ± 0.03	2.37 ± 0.20	34.86 ± 0.78	20.78 ± 1.09	24.17 ± 0.57	2.75 ± 0.05	2.46 ± 0.06
	MW	300 W	-	-	17.30 ± 1.67	22.69 ± 1.30	2.80 ± 0.06	2.35 ± 0.20	38.50 ± 0.89	21.57 ± 0.20	30.14 ± 0.44	3.21 ± 0.17	3.82 ± 0.08
	Percolation	-	-	-	18.16 ± 1.34	23.87 ± 2.14	2.89 ± 0.12	3.01 ± 0.22	34.07 ± 2.70	-	-	-	-
Flowering tops (Ground 1 mm)	Infusion	250 rpm	<1.5	2.27 ^a^	-	-	-	-	-	-	-	-	-
		500 rpm	<1.5	2.41 ^a^	22.20 ± 0.59	32.79 ± 0.67	3.45 ± 0.20	3.93 ± 0.09	34.35 ± 0.48	23.19 ± 0.66	34.67 ± 0.93	3.56 ± 0.06	4.63 ± 0.39
		750 rpm	<1.5	2.43 ^a^	-	-	-	-	-	-	-	-	-
		1000 rpm	<1.5	2.52 ^a^	-	-	-	-	-	-	-	-	-
	Maceration (at 500 rpm)	20 °C	12	1.76	-	-	-	-	-	19.11 ± 0.35	-	-	-
		40 °C	4.5	2.07 ^a^	-	-	-	-	-	19.20 ± 0.48	-	-	-
		60 °C	<1.5	2.30 ^a^	20.59 ± 0.29	28.45 ± 0.26	3.12 ± 0.07	3.52 ± 0.14	33.85 ± 0.80	21.18 ± 1.29	30.28 ± 0.37	3.20 ± 0.07	3.78 ± 0.08
		80 °C	<1.5	2.58 ^a^	-	-	-	-	-	23.30 ± 0.78	-	-	-
	US (at 250 rpm)	20 °C	-	-	-	-	-	-	-	14.28 ± 1.67	-	-	-
		40 °C	-	-	-	-	-	-	-	17.50 ± 1.72	-	-	-
		60 °C	<1.5	3.05 ^a^	24.24 ± 0.98	33.58 ± 1.23	3.64 ± 0.47	4.29 ± 0.10	32.67 ± 1.08	25.10 ± 1.86	33.26 ± 1.72	3.74 ± 0.17	4.32 ± 0.19
	MW	300 W	-	-	23.28 ± 0.42	34.73 ± 1.57	3.93 ± 0.17	4.04 ± 0.08	34.40 ± 0.49	23.81 ± 1.37	37.31 ± 0.87	3.85 ± 0.05	4.73 ± 0.21
	Percolation	-	-	-	18.98 ± 0.64	27.15 ± 1.78	2.95 ± 0.20	3.62 ± 0.13	34.59 ± 1.90	-	-	-	-
Flowering tops (Fresh)	Infusion	500 rpm	16.5	0.72	10.07 ± 1.02	8.28 ± 0.66	1.19 ± 0.05	0.41 ± 0.04	51.07 ± 4.09	12.27 ± 1.22	10.41 ± 0.66	1.60 ± 0.19	0.86 ± 0.08
	Maceration	60 °C, 500 rpm	13	0.58	6.69 ± 1.84	2.94 ± 1.38	0.63 ± 0.06	0.14 ± 0.04	-	11.09 ± 0.78	9.80 ± 0.89	1.35 ± 0.09	0.65 ± 0.05
Flowers only (Raw)	Infusion	500 rpm	-	-	10.18 ± 0.87	11.50 ± 0.32	1.66 ± 0.01	0.66 ± 0.05	21.55 ± 0.34	-	-	-	-

**Table 3:** Comparison of the total phenol content (TPC), total flavonoid content (TFC) and total proanthocyanidin oligomer content (OPC) contents between commercially available standardized extracts (as given by the supplier) and infusions at 10 min extraction time (as determined in this work). Q = quercetin, CY = cyanidin, GA = gallic acid, HY = hyperoside, epiCAT = epicatechin. ^a^ as for 2017.

Standardized Extracts	Uptake Quantity	Dry Extract Content	Dry Plant: Dry Extract Ratio	Plant Organ	Excipients	Extraction Solvent	TPC	TFC	OPC	Price (€)	Treatment Cost/Day (cents)	Cost/30 Days (€) ^a^
EPS Phyto-prevent^®^	5–10 mL	900 mg/5 mL	-	Leaves & flowers	Glycerol	-	-	7.5–12.5 mg eq. Q/5 mL (0.8–1.4 %)	-	19.89/150 mL	66.3/5 mL	19.9
WS1442^®^ crataegutt novo 450 (*or* Cardiplant^®^ 450 *or* Cardio Max WS 1442^®^)	1–2 tablets	450 mg/1 tablet	4.0–6.6:1	Leaves & flowers	Glucose, SiO_2_, Fe_2_O_3_, TiO_2_, sucrose, gelatine, macrogol, citric acid, …	EtOH (45 %)	-	-	78–90.6 mg eq. epiCAT/1 tablet (17.3–20.1 %)	16.13/50 tablets	64.5/2 tablets	19.4
Crataegisan ^®^ Bioforce	30–90 drops (0.75−2.25 mL)	690 mg/90 drops	3.2:1	Fruits	EtOH (46−54%)	EtOH (50 %)	12.7 mg eq. GA/90 drops (1.84 %)	-	6.4 mg/90 drops (0.93%)	10.50/50 mL	52.5/90 drops	15.8
Faros 300^®^ LI 132	3 Tablets	300 mg/1 tablet	4.0–7.0:1	Leaves, flowers & fruits	Glucose, SiO_2_, lactose, TiO_2_, sucrose, gelatine, macrogol, …	MeOH (70 %)	-	6.6 mg eq. HY/1 tablet (2.2 %)	-	23.25/100 tablets	69.7/3 tablets	20.9
Infusion (lot no. 20335)	1–2 infusions	555 mg/10 min infusion (from 2.5 g ground dry plant)	4.3–4.6:1	Leaves & flowers	Water	Water	82 mg eq. GA/1 infusion	8.6 mg eq. Q/1 infusion	9.8 mg eq. CY/1 infusion	29.25/1 kg	7.3/1 infusion	2.2

**Table 4:** Peak identification of the main compounds detected by UHPLC in the various hawthorn extracts. *λ*_max_ is the local maximum of absorbance on the UV spectrum. The [M + H]^+^ column provides the *m*/*z* value of the precursor ion. The other ions column gives the *m*/*z* value of fragments detected in the MS spectra. Identification method using UV spectrum and ESI (+) spectrum of the pure standards (R) or a secondary standard mixture (R1, Crataegus spp. extract).

Peak	Retention Time (min)	*λ*_max_ (nm)	[M + H]^+^	Other Ions in the Spectrum	Identified Compound	Standard Used for Identification	Ref.
1	2.71	204, 218, 260	288		Cyanidin	R	[40]
2	3.73	218, 236, 324	355	377, 711	5-*O*-Caffeoylquinic acid	R1	[40]
3	7.49	219, 238, 325	355	377, 711	Chlorogenic acid (3-*O*-caffeoyquinic acid)	R1	[40]
4	9.1	227, 280	579	427, 289	Procyanidin B2	R	[40]
5	9.45	224 279	291	147, 139, 123	Epicatechin	R	[40]
6	12.24	280	867	579	Procyanidin C1	R	[40]
7	13.37	219, 280	1155	287, 413, 575	Cinnamtannin A2	R	[40]
8	15.59	216, 269, 338	579	433, 313	Vitexin 2-O-rhamnoside	R1	[40]
9	16.13	220, 256, 353	465	303	Hyperoside	R1	[40]
10	16.35	219, 280	577	289	Procyanidin A2	R	[40]
11	16.52	202, 257, 353	303	621	Isoquercetin	R	[40]
12	19.85	268, 337	433	621	Apigenin-C-hexoside	R1	[40]

**Table 5:** Extraction yield, TPC, TFC, and OPC values in hawthorn extracts issued from infusion mode at 10 min extraction time. Influence of the particle size (plant state), the stirring, the use of a Celia^®^ bag, the nature of the container, and the nature of the plant (different lot of dry flowering tops, dry flowers, or fresh flowering tops). Experimentally, 2.5 g of hawthorn material in 125 mL (250 mL and 405 mL, respectively) water was used for the cup (resp. mug or Bodum^®^ and bowl recipients). 🟀: see Table 2 for more information. * Manual stirring was done by manually rotating the recipient at the beginning of the extraction and 10′ later before filtration.

Celia^®^ Bag	Stirring	Lot Number	Plant Organs	Plant Material	Container	TPC mg eq. GA ^🟀^	TFC mg eq. Q ^🟀^	OPC mg eq. CY ^🟀^	Extraction Yield (%)	Vitexin 2-*O*-rhamnoside (mg)
No	Yes	55849	Flowering tops	Ground 1 mm	Cup	26.6 ± 1.6	2.88 ± 0.17	2.24 ± 0.18	19.8 ± 0.9	-
					Mug	33.5 ± 1.5	3.71 ± 0.19	3.40 ± 0.16	21.7 ± 2.0	-
					Bowl	35.9 ± 0.9	4.27 ± 0.19	3.77 ± 0.23	23.1 ± 0.2	-
				Ground 2 mm	Cup	26.9 ± 0.7	2.57 ± 0.10	2.23 ± 0.09	21.5 ± 0.1	-
					Mug	32.2 ± 0.4	3.42 ± 0.23	3.07 ± 0.02	24.1 ± 0.2	-
					Bowl	35.3 ± 1.2	3.92 ± 0.21	3.56 ± 0.22	24.2 ± 0.3	-
Yes	Yes	55849	Flowering tops	Ground 1 mm	Mug	24.9 ± 0.2	3.01 ± 0.22	2.29 ± 0.05	16.9 ± 1.0	-
Yes	Yes	CB58120	Flowering tops	Raw	Mug	13.6 ± 0.3	1.71 ± 0.02	0.70 ± 0.06	16.8 ± 0.1	-
				Fine		20.6 ± 1.6	3.02 ± 0.11	1.72 ± 0.08	22.3 ± 0.5	-
				Ultrafine 10″		10.2 ± 0.4	1.45 ± 0.11	0.73 ± 0.05	11.7 ± 0.3	-
No	Yes	CB58120	Flowering tops	Fine	Bodum^®^	21.6 ± 0.4	3.13 ± 0.10	1.81 ± 0.09	22.5 ± 0.3	-
No	No *	CB58120	Flowering tops	Fine	Bodum^®^	20.1 ± 0.4	2.86 ± 0.02	1.81 ± 0.05	21.7 ± 0.1	25.9 ± 0.3
				Coarse		21.8 ±0.1	2.46 ±0.15	1.64 ±0.07	21.1 ±0.4	-
				Ultrafine 10″		21.3 ± 0.4	2.98 ± 0.16	1.85 ± 0.06	21.7 ± 0.3	-
		H18001534		Fine		34.2 ± 1.8	3.67 ± 0.21	1.76 ± 0.05	22.4 ± 0.5	14.7 ± 0.3
		1221478		Fine		28.0 ± 1.3	3.66 ± 0.19	1.64 ± 0.06	22.0 ± 0.3	10.7 ± 0.9
		R78925		Fine		23.8 ± 0.9	2.98 ± 0.15	1.21 ± 0.06	22.4 ± 0.1	25.0 ± 1.5
No	No *	20334	Flowers	Fine	Bodum^®^	37.2 ± 0.7	3.46 ± 0.09	1.96 ± 0.05	21.7 ± 0.6	15.5 ± 0.5
No	No *	-	Fresh (after 1 year)	Fine	Bodum^®^	44.6 ± 1.3	4.06 ± 0.14	4.24 ± 0.17	27.8 ± 0.5	25.6 ± 1.2

## Supplementary Materials

The following are available online: Figure S1: Picture and localization of fresh hawthorn; Figure S2: Picture of the experimental set-up used for each extraction mode; Figure S3: Extraction kinetics of ground (1 mm) hawthorn followed by UV absorbance at 198 nm for various extraction modes; Figure S4: UV absorbance values at 198 nm and different extraction times as a function of the extraction yields at the corresponding time for all extraction modes; Figure S5: UHPLC profiles of hawthorn extracts obtained from different extraction modes for raw hawthorn; Figure S6: Relative proportions of the main compounds detected by UHPLC in the various hawthorn extracts as a function of the extraction mode, the granulometry, the nature, and the state of the plant; Figure S7: Chemical structures of all compounds identified by UHPLC-ESI-MS; Figure S8: Mass spectra achieved by (−) ESI FT-ICR MS analysis of the hawthorn samples according to the extraction method, plant parts, and state (fresh or dry); Figure S9: Hierarchical cluster analysis (HCA), and heatmap achieved from samples analyzed by (−) ESI FT-ICR MS; Figure S10: Pictures of cup, mug, and bowl with dimensions and weights; Figure S11: Decrease profile of temperature vs. the nature of the container (cup, mug, bowl, Bodum^®^, three-neck flask); Figure S12: Pictures of raw and ground hawthorn materials of various granulometries; Figure S13: Size distributions of ground hawthorn materials; Figure S14: Influence of the lot number of ground (fine granulometry) hawthorn dry flowering tops and one lot of raw dry flowers on the UHPLC profiles of corresponding hawthorn extracts; Figure S15: Relative proportions of the main compounds (relative peak area) detected by UHPLC-UV in the various ground (fine granulometry) hawthorn extracts as a function of the lot number of dry flowering tops and one lot of raw dry flowers; Table S1: List of the samples analyzed by UHPLC-ESI-MS and (−) ESI FT-ICR-MS; Table S2: Fitting parameters for the absorbance *A(t)* trace vs. extraction time t for the infusion mode; Table S3: Compounds identified in hawthorn putatively assigned to raw formulae achieved by (−) ESI FT-ICR MS; Table S4: Putative compounds obtained from features specifically extracted depending on the plant states (fresh vs. dry and ground vs. raw) or parts (flowers vs. flowering tops).
