# Supplementary material for: Optimizing Water-Based Extraction of Bioactive Principles of Hawthorn: From Experimental Laboratory Research to Homemade Preparations
Source: Molecules. 2019 Dec 3;24(23):4420. doi: 10.3390/molecules24234420 (PMC6930565; doi:10.3390/molecules24234420)
Supplement: Supplementary file 1 [file molecules-24-04420-s001.pdf]

SUPPORTING INFORMATION

Optimizing Water-based Extraction of Bioactive Principles of Hawthorn: from Experimental Laboratory Research to Homemade Preparations

Phu Cao Ngoc [1], Laurent Leclercq [1,*], Jean-Christophe Rossi [1], Isabelle Desvignes [1], Jasmine Hertzog [2,3], Anne-Sylvie Fabiano-Tixier [4], Farid Chemat [4], Philippe Schmitt-Kopplin [2,3] and Hervé Cottet [1,*]

[1] IBMM, University of Montpellier, CNRS, ENSCM, Montpellier, France
[2] Analytical BioGeoChemistry, Helmholtz Zentrum Muenchen, Neuherberg, Germany
[3] Analytical Food Chemistry, Technische Universität Muenchen, Freising, Germany
[4] University of Avignon, INRA, UMR408, GREEN Extraction Team, Avignon, France

* Correspondence: herve.cottet@umontpellier.fr (H.C.) and laurent.leclercq@umontpellier.fr (L.L.)

Table of Content :

Figure S1. Picture (A) and localization (B) of fresh hawthorn (marked in red). 2

Figure S2. Picture of the experimental set-up used for each extraction mode 3

Figure S3. Extraction kinetics of grinded (1 mm) hawthorn followed by UV absorbance at 198 nm for various extraction modes. 4

Figure S4. UV absorbance values at 198 nm and at different extraction times (A: 5 min and B: 30 min) as a function of the extraction yields at the corresponding time for all extraction modes. 5

Figure S5. UPLC profiles of hawthorn extracts obtained from different extraction modes for raw hawthorn... 6

Figure S6. Relative peak area distributions for the main compounds detected by UHPLC in the various hawthorn extracts as a function of the extraction mode, the granulometry, the nature and the state of the plant. 7

Figure S7. Chemical structures of all compounds identified by UHPLC-ESI-MS. 8

Figure S8. Mass spectra achieved by (-) ESI FT-ICR MS analysis of the hawthorn samples, in duplicate (green and red mass spectra), according to the extraction method, plant parts and state (fresh or dry). 11

Figure S9. Hierarchical Cluster Analysis (HCA) and heatmap achieved from samples analyzed by (-) ESI FT-ICR MS. 24

Figure S10. Pictures of cup (A), mug (B) and bowl (C) with dimensions and weights. 25

Figure S11. Decrease profile of temperature (without stirring) *vs* the nature of the container (cup, mug, bowl, Bodum®, three-neck flask) 26

Figure S12. Pictures of raw and grinded hawthorn materials of various granulometries. 27

Figure S13. Size distributions of grinded hawthorn materials. 28

Figure S14. Influence of the lot number of grinded (fine granulometry) hawthorn dry flowering tops (R78927, 1221478, H18001534, CB58120) and one lot of raw dry flowers (20334) on the UHPLC profiles of the corresponding hawthorn extracts. 31

Figure S15. Relative peak area distributions for the main compounds detected by UHPLC-UV in the various grinded (fine granulometry) hawthorn extracts as a function of the lot number of dry flowering tops (R78927, 1221478, H18001534, CB58120) and one lot of raw dry flowers (20334). 32

Table S1. List of the samples analyzed by UHPLC-ESI-MS and (-)ESI FT-ICR-MS. 33

Table S2. Fitting parameters for the absorbance A(*t*) trace *vs* extraction time *t* for the infusion mode.. 34

Table S3. Compounds identified in hawthorn putatively assigned to raw formulae achieved by (-) ESI FT-ICR MS. 35

Table S4. Putative compounds obtained from features specifically extracted depending on the plant states (fresh vs dry and grinded vs raw) or parts (flowers vs flowering tops) 36

Figure S1. Picture (A) and localization (B) of fresh hawthorn (marked in red).

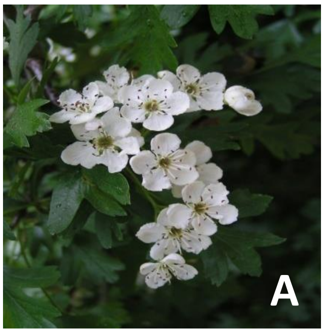

Hawthorn collected on a wild tree located at Le Grand-Village-Plage, F-17370, Oléron Island, France.

GPS coordinates: 45°51'57.5"N 1°13'45.3"W

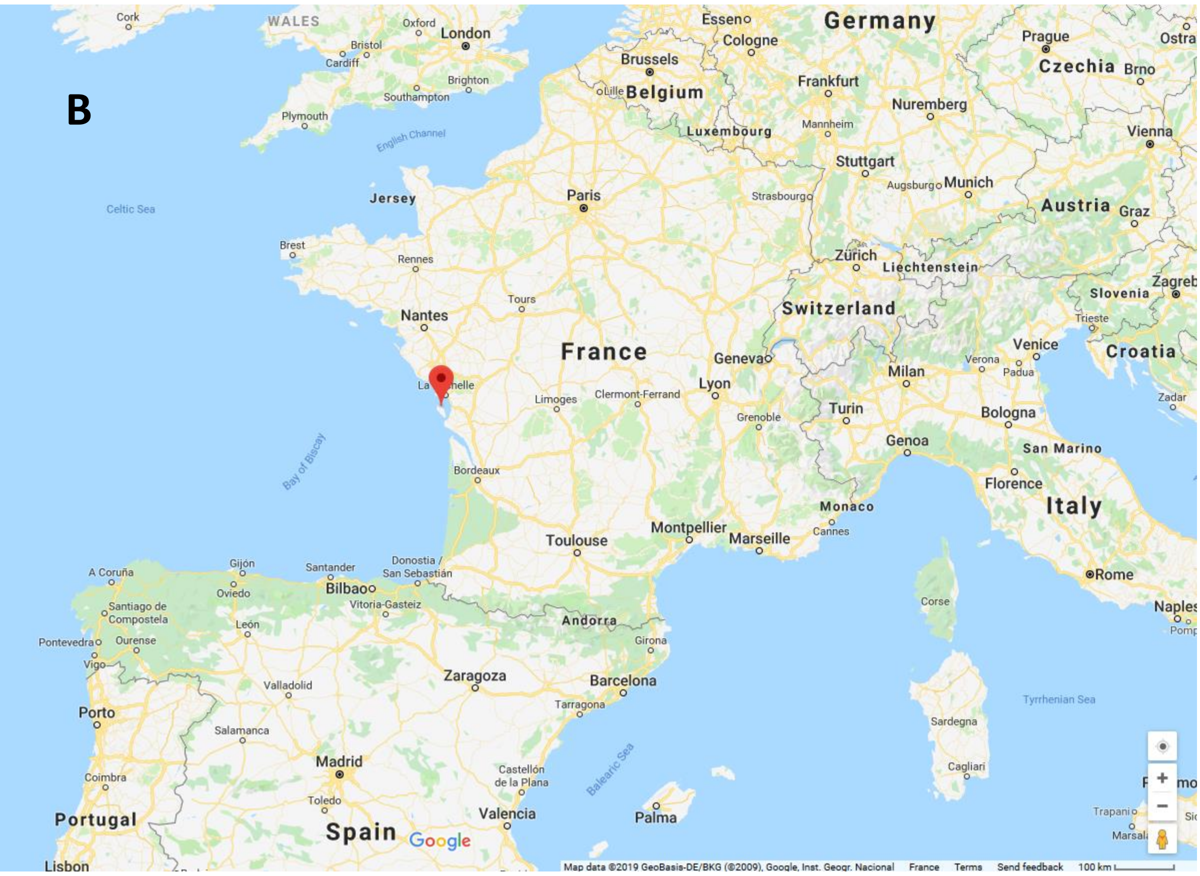

Figure S2. Picture of the experimental set-up used for each extraction mode (A: infusion; B: maceration; C: percolation; D: ultrasonic; E: Microwave; F: infusion using a French-Press Bodum® (with or without stirring)).

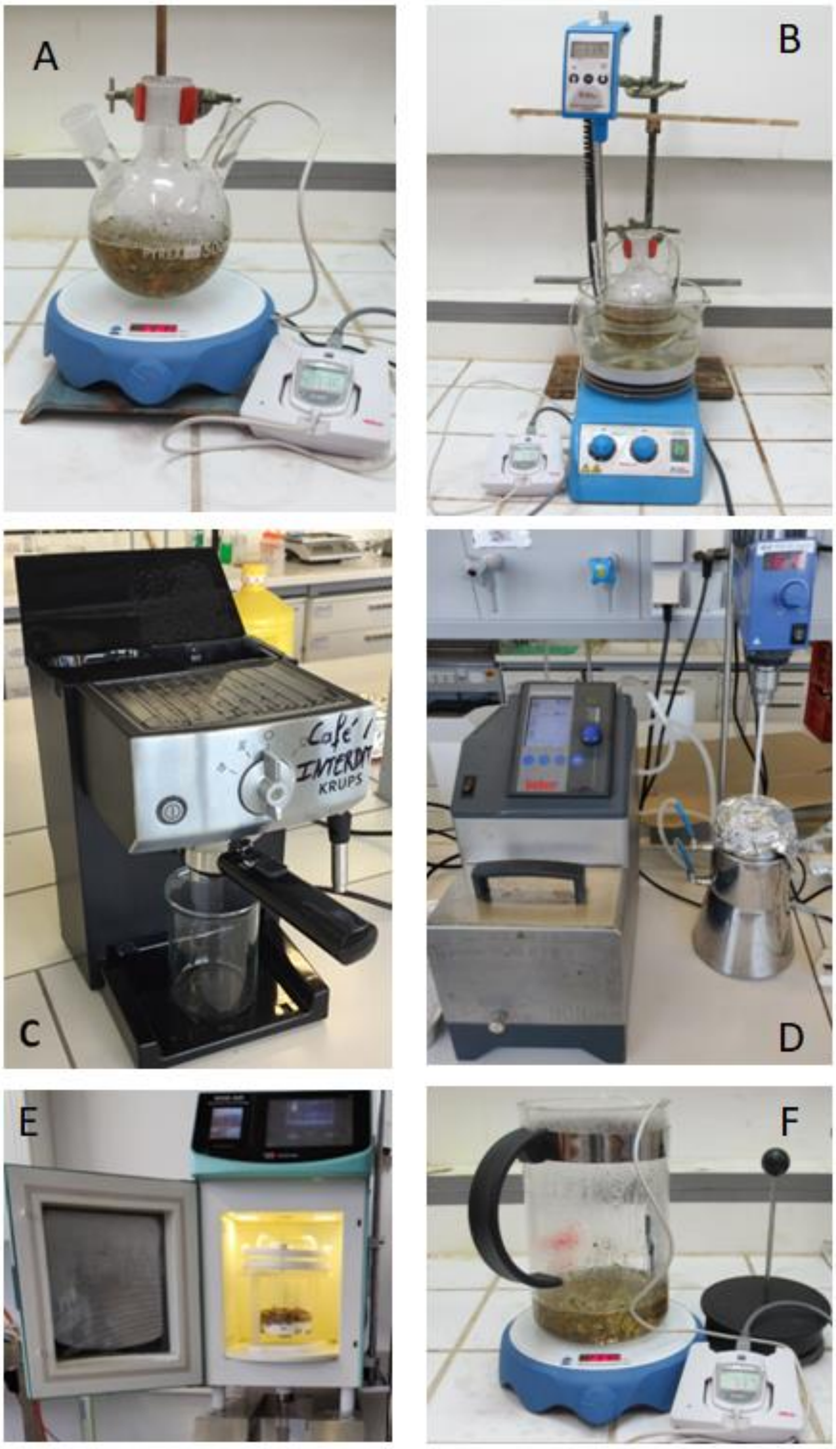

Figure S3. Extraction kinetics of grinded (1 mm) hawthorn followed by UV absorbance at 198 nm for various extraction modes. A: infusion mode at 250 rpm, 500 rpm, 750 rpm and 1000 rpm stirring speed, including the temperature profile at 500 rpm. B: maceration mode at 20°C, 40°C, 60°C and 80°C and at 500 rpm stirring speed. C: ultrasonic mode at 60°C and at 250 rpm stirring speed. In all cases, 2.5 g of raw hawthorn in 250 mL water was used. 100 μL of solution were taken and added to 4 mL (or 8 mL if the absorbance values were above 1.7) water before each UV measurement. Error bars are ± one SD on n = 3 repetitions of independent extractions.

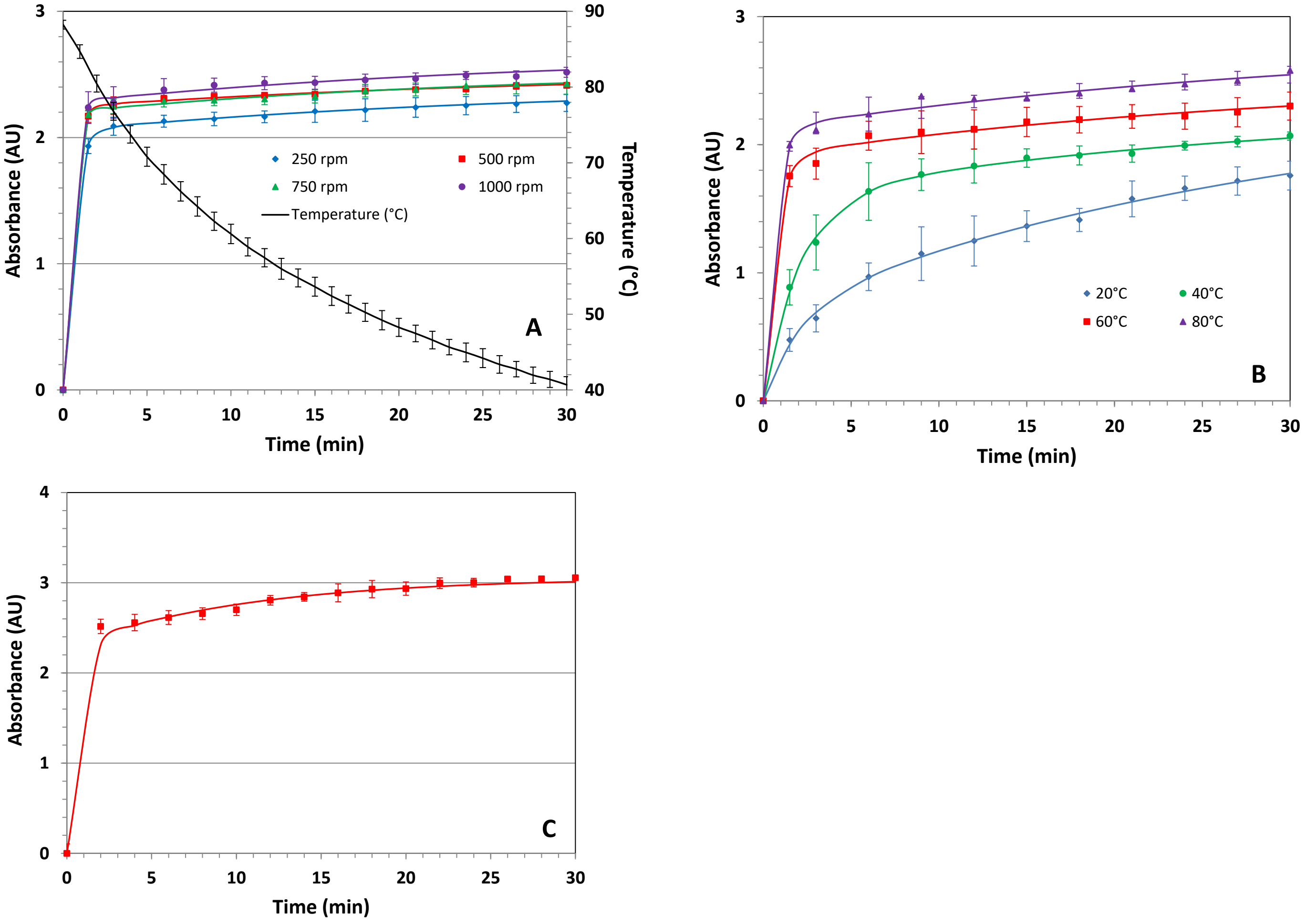

Figure S4. UV absorbance values at 198 nm and at different extraction times (A: 5 min and B: 30 min) as a function of the extraction yields at the corresponding time for all extraction modes. In all cases, 2.5 g of hawthorn material in 250 mL water was used. Maceration and ultrasonic extractions at 60°C, 100 µL of solution were taken and added to 4 mL water before UV measurement. For all the absorbance values above 1.6, and to avoid the saturation of the detector, the solutions were diluted twice (the absorbance values were multiplied by 2 for better comparison). Error bars are ± one SD on *n* = 3 repetitions of independent extractions.

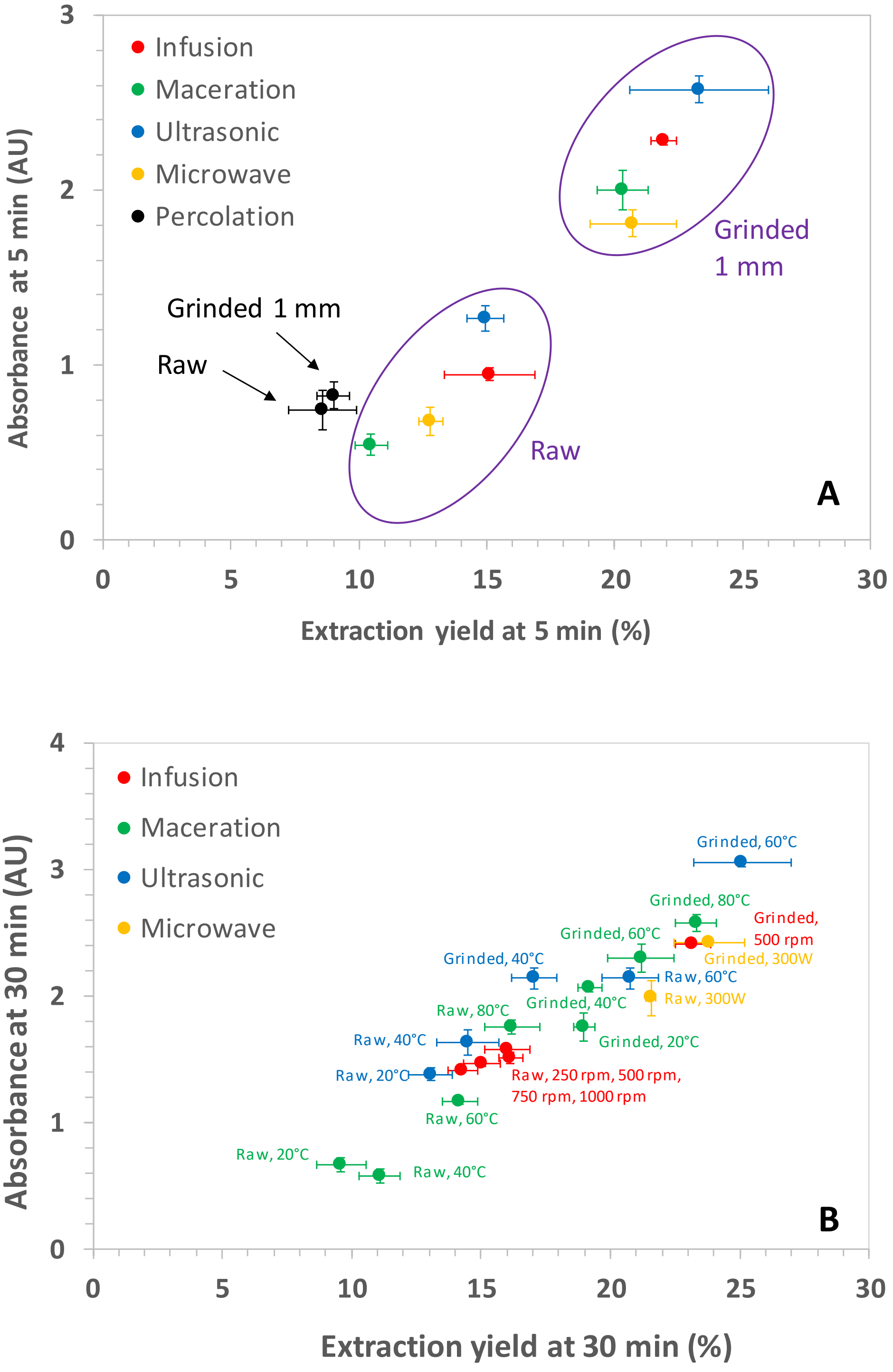

Figure S5. UPLC profiles of hawthorn extracts obtained from different extraction modes for raw hawthorn. All dry plant extracts are issued from lot n°20335. Experimental conditions: Luna® Omega polar C18 column (1.6 μm, 100 × 2.1 mm), binary solvent system: water/formic acid (1‰, v/v) as solvent A and acetonitrile/formic acid (1‰, v/v) as solvent B. Gradient program: 5 % B, then increase of B to 100 % in 30 min with a convex increase. Flow rate: 0.4 $mL.min^{-1}$. Injection volume: 4 μL. Column temperature: 35°C. UV monitoring at 273 nm.

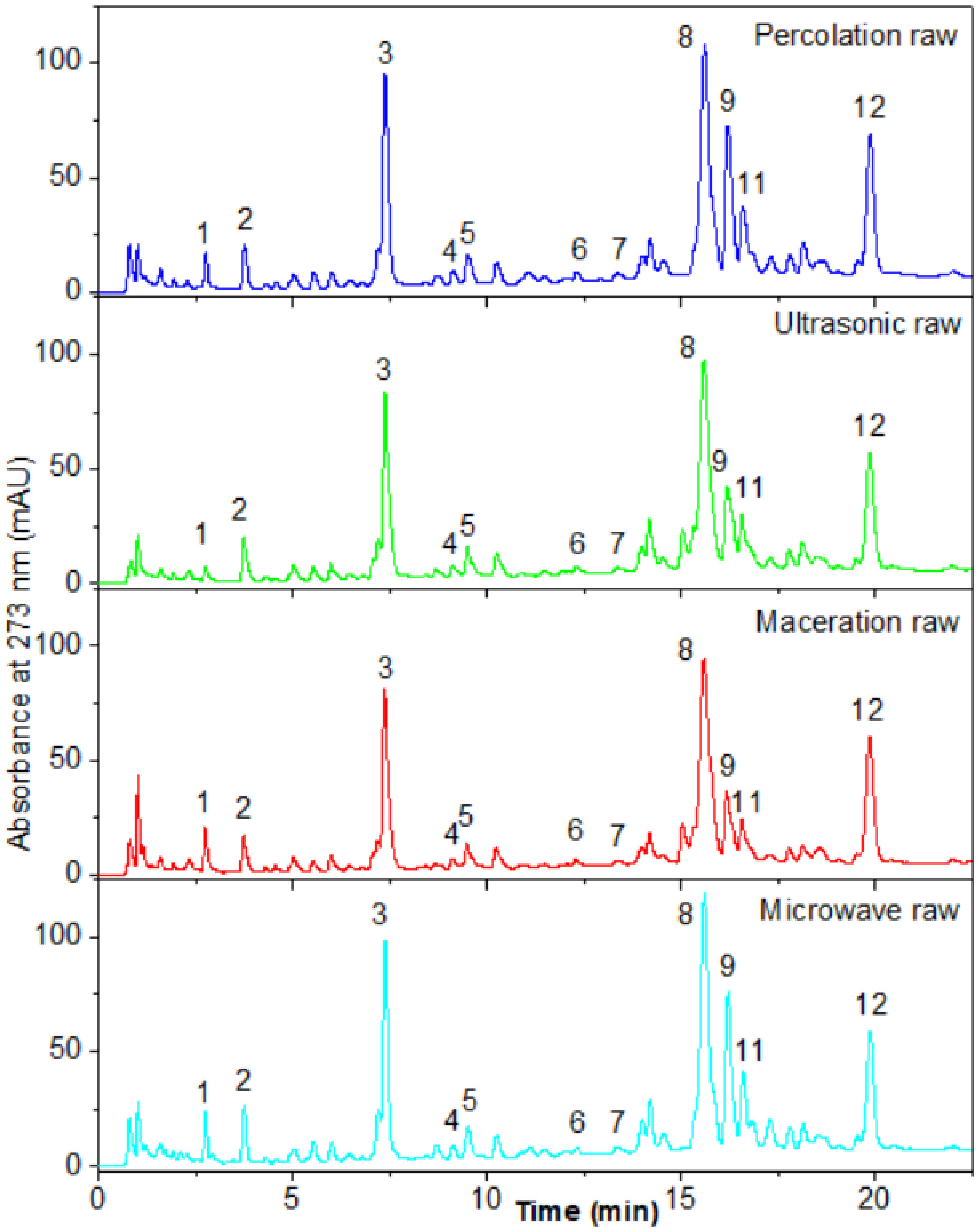

Figure S6. Relative peak area distributions for the main compounds detected by UHPLC in the various hawthorn extracts as a function of the extraction mode, the granulometry, the nature and the state of the plant. The relative area was calculated by dividing the peak area of each component by the sum of the peak area of the 12 identified components. Experimental conditions as in Figure S5.

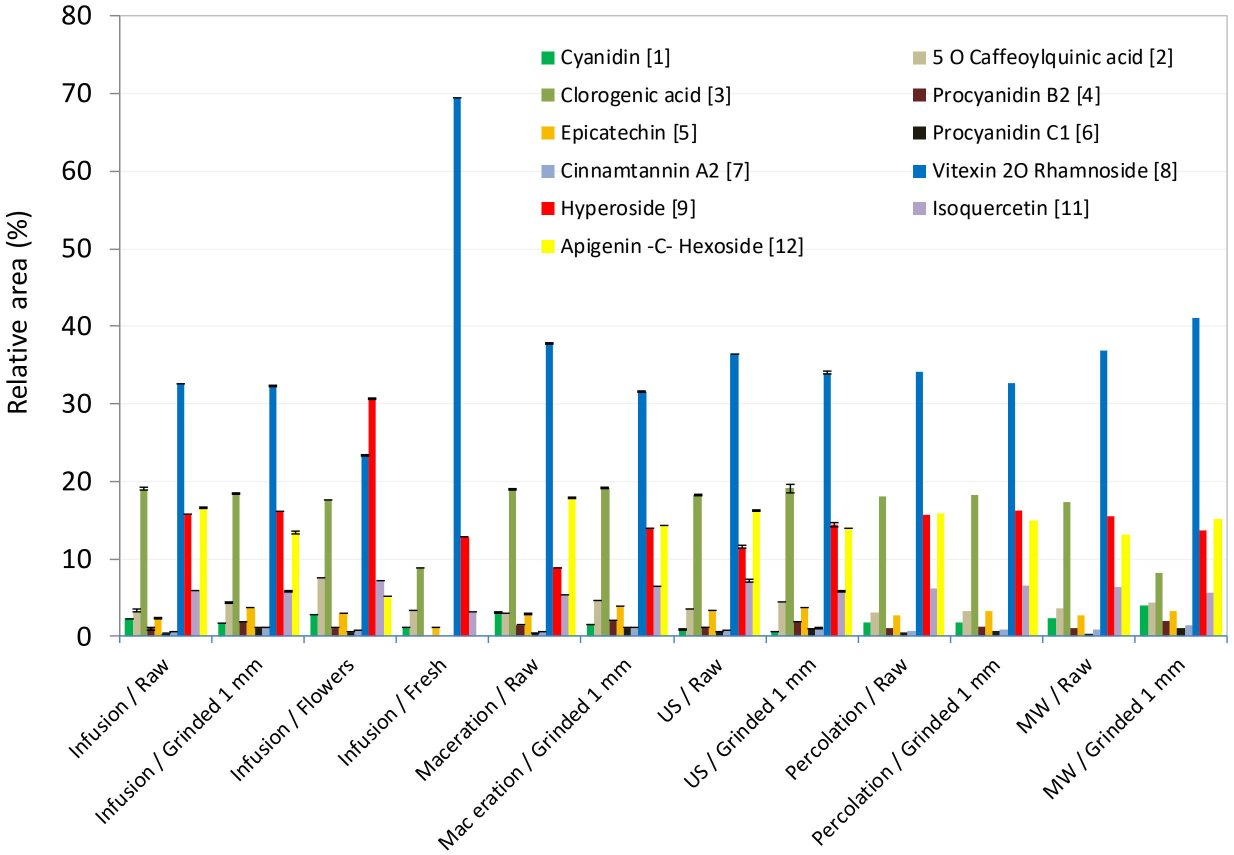

Figure S7. Chemical structures of all compounds identified by UHPLC-ESI-MS.

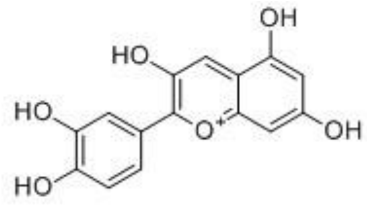

Chemical Formula: $C_{15}H_{11}O_6^+$
Exact Mass: 287,0550

Peak 1: Cyanidin

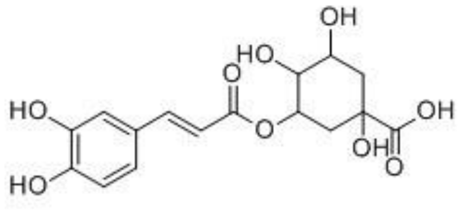

Chemical Formula: $C_{16}H_{18}O_9$
Exact Mass: 354,0951

Peak 2: 5-O-Caffeoylquinic acid or Neochlorogenic acid

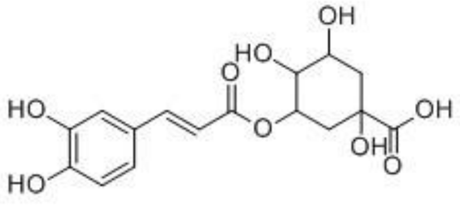

Chemical Formula: $C_{16}H_{18}O_9$
Exact Mass: 354,0951

Peak 3: Chlorogenic acid

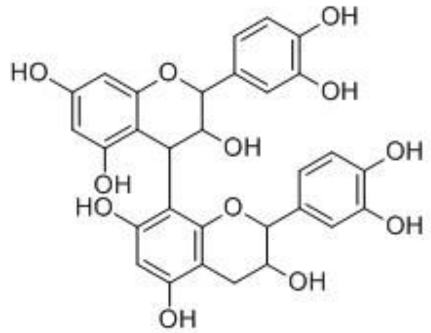

Chemical Formula: $C_{30}H_{26}O_{12}$
Exact Mass: 578,1424

Peak 4: Procyanidin B2

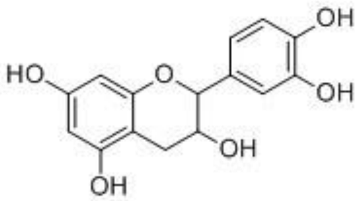

Chemical Formula: $C_{15}H_{14}O_6$
Exact Mass: 290,0790

Peak 5: Epicatechin

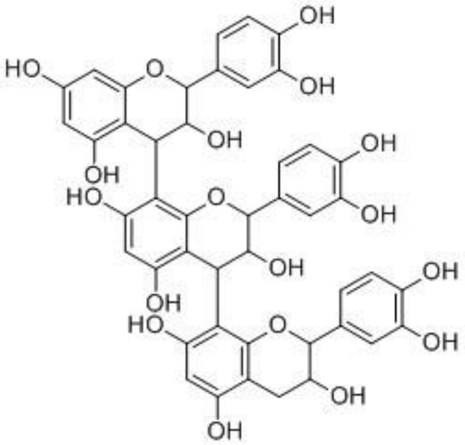

Chemical Formula: $C_{45}H_{38}O_{18}$
Exact Mass: 866,2058

Peak 6: Procyanidin C1

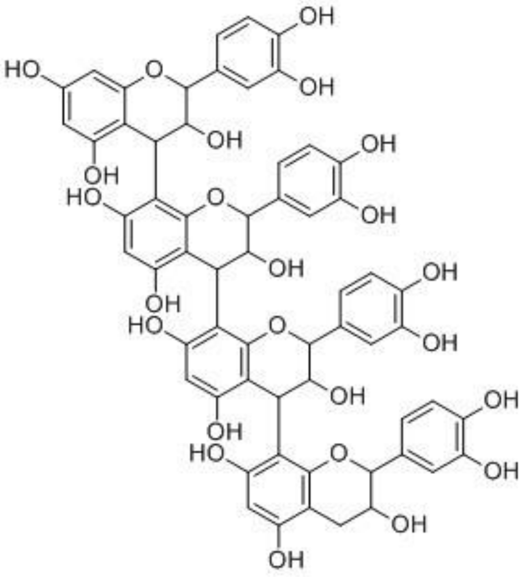

Chemical Formula: $C_{60}H_{50}O_{24}$
Exact Mass: 1154,2692

Peak 7: Cinnamtannin A2

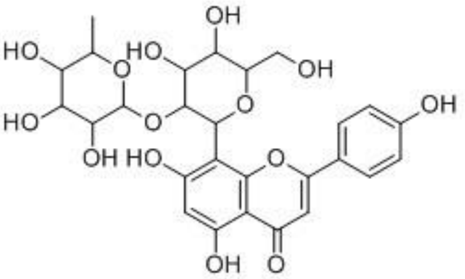

Chemical Formula: $C_{27}H_{30}O_{14}$
Exact Mass: 578,1636

Peak 8: Vitexin 2-O-rhamnoside

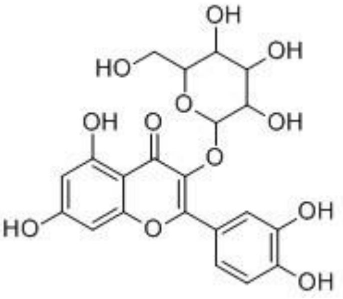

Chemical Formula: $C_{21}H_{20}O_{12}$
Exact Mass: 464,0955

Peak 9: Hyperoside

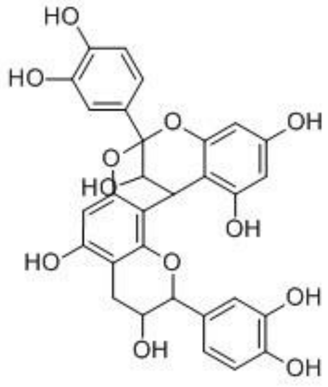

Chemical Formula: $C_{30}H_{24}O_{12}$
Exact Mass: 576,1268

Peak 10: Procyanidin A2

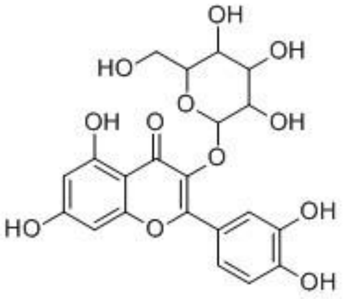

Chemical Formula: $C_{21}H_{20}O_{12}$
Exact Mass: 464,0955

Peak 11: Isoquercetin

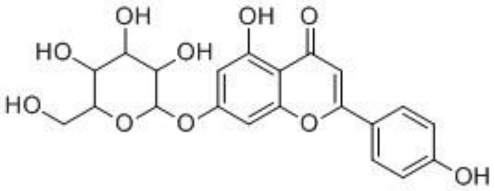

Chemical Formula: $C_{21}H_{20}O_{10}$
Exact Mass: 432,1056

Peak 12: Apigenin-C-hexoside
or Apigenin 7-glucoside

Figure S8. Mass spectra achieved by (-) ESI FT-ICR MS analysis of the hawthorn samples, in duplicate (green and red mass spectra), according to the extraction method, plant parts and state (fresh or dry). The pie charts show the heteroatom class distribution and the achieved corresponding feature numbers. Van Krevelen diagram represents all the assigned features coloured by chemical class. The size of the bubble is relative to the peak intensity.

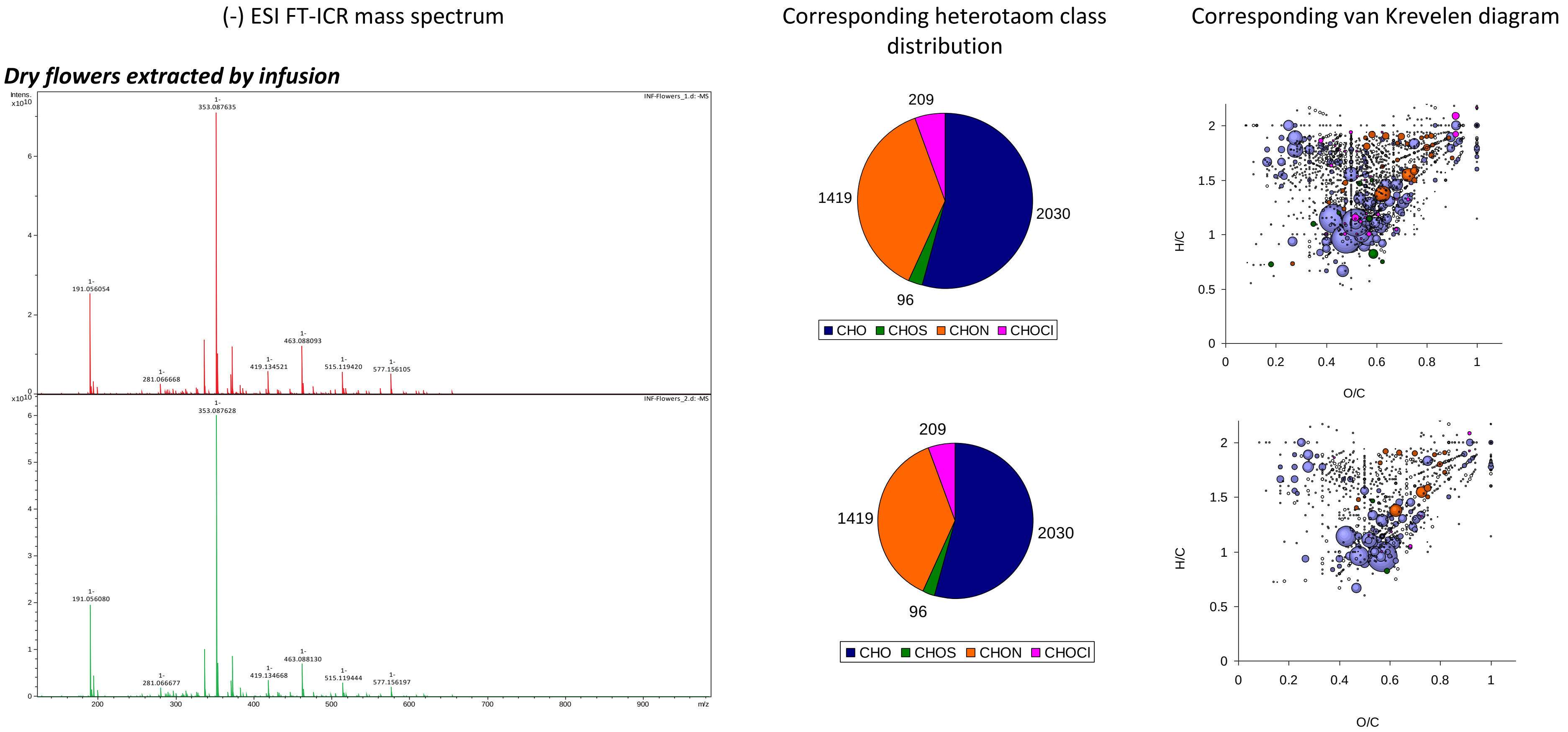

Fresh flowering tops extracted by infusion

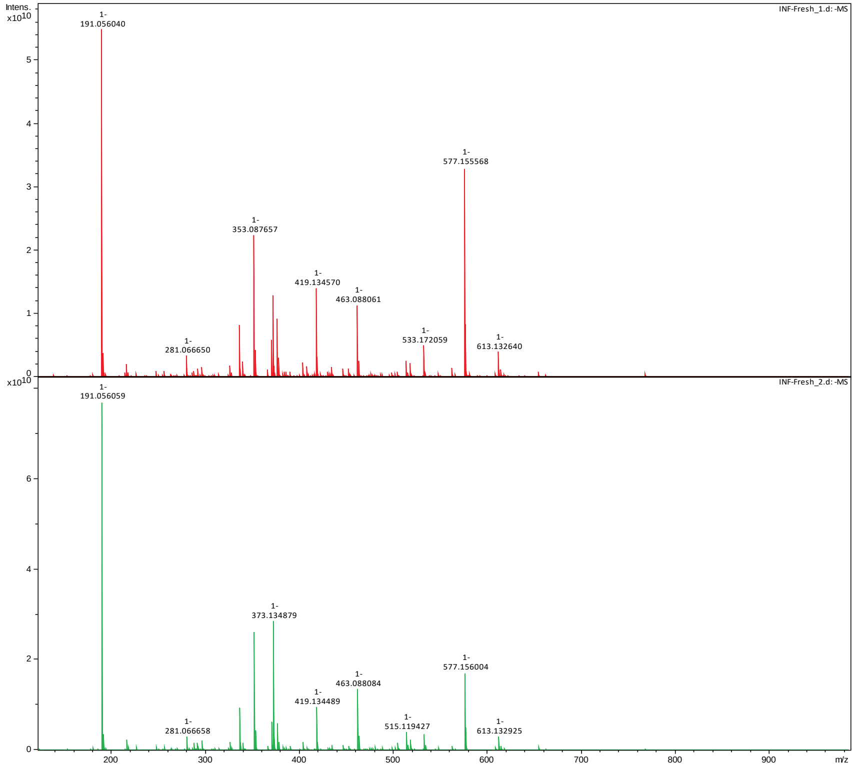

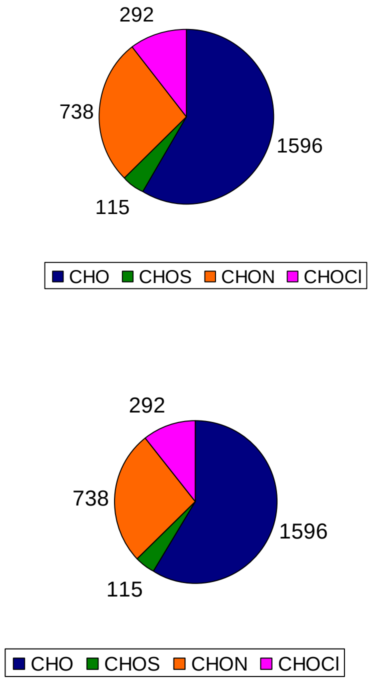

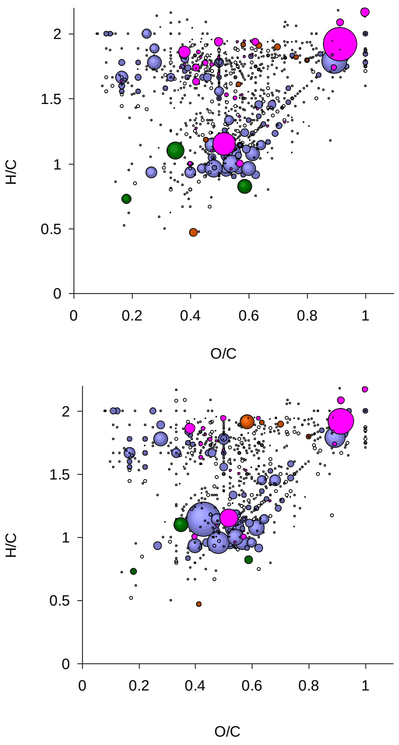

Grinded dry flowering tops extracted by infusion

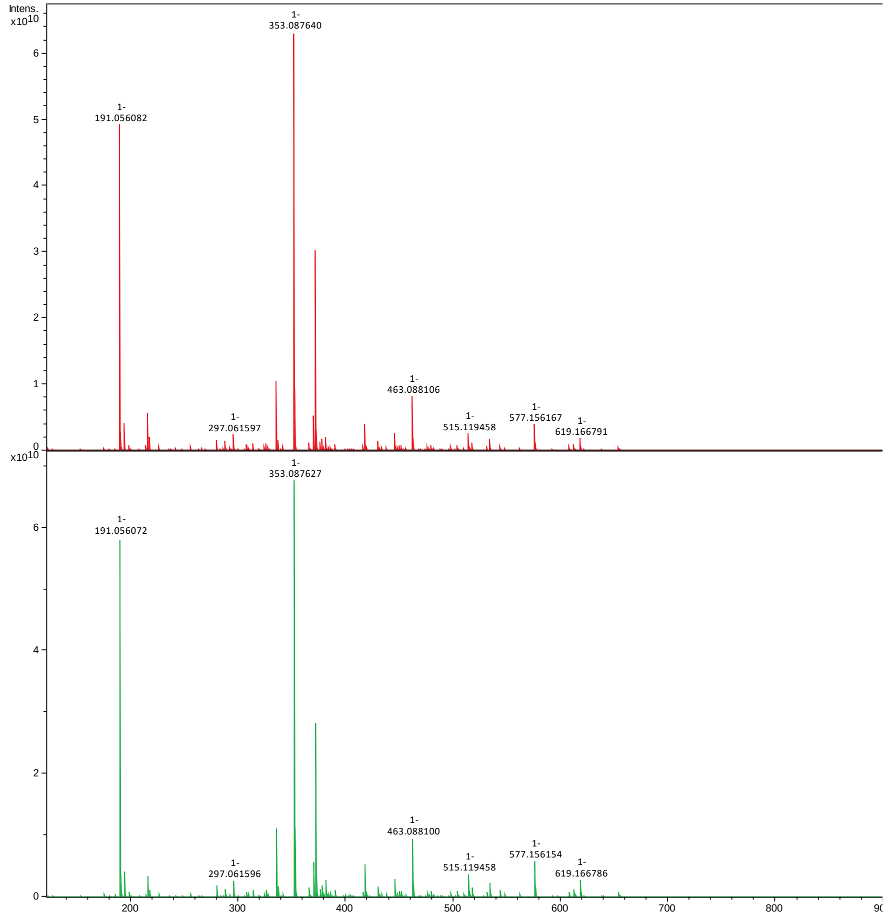

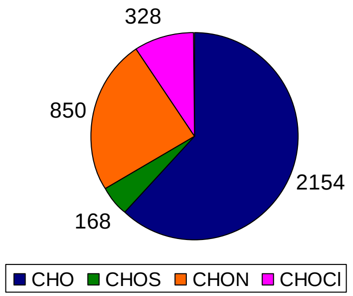

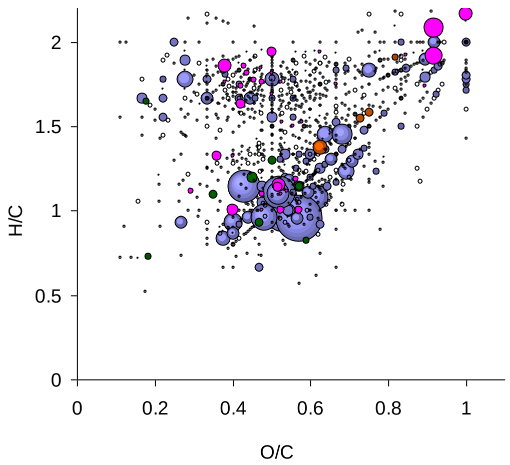

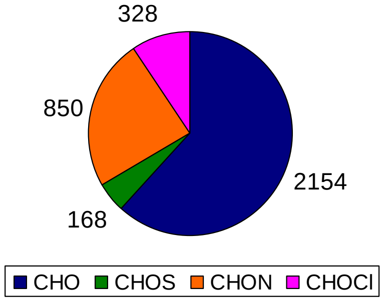

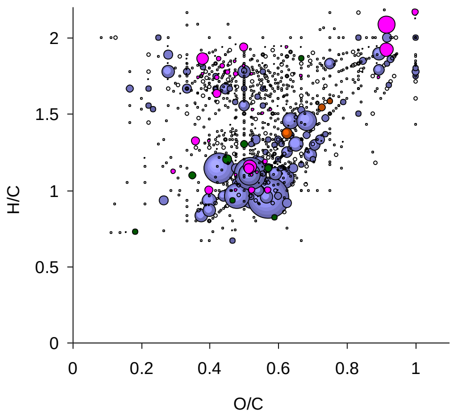

Raw dry flowering tops extracted by infusion

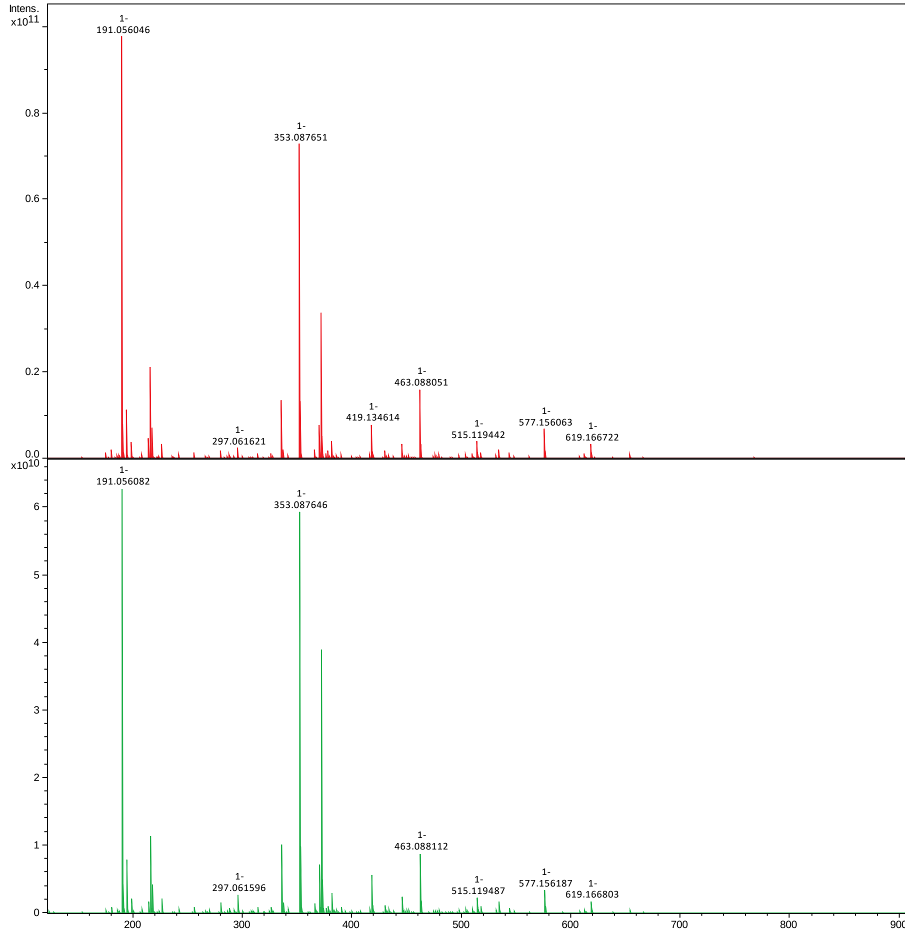

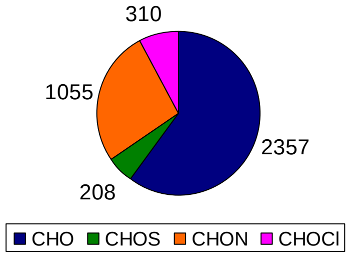

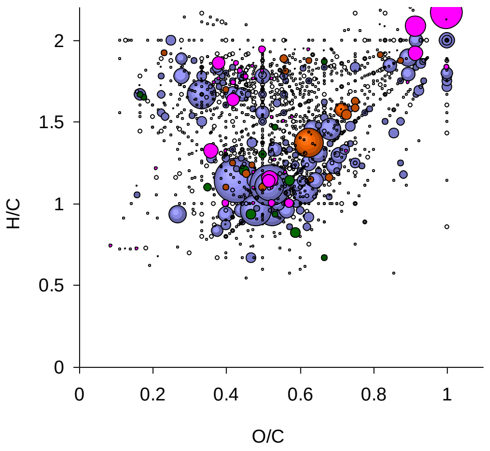

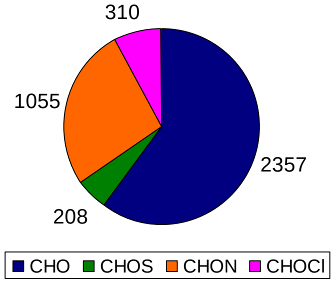

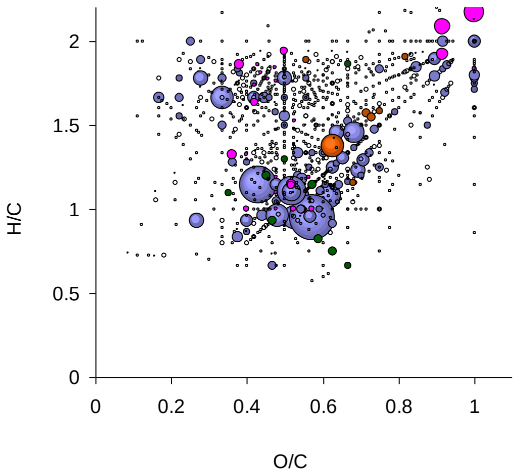

Grinded dry flowering tops extracted by maceration

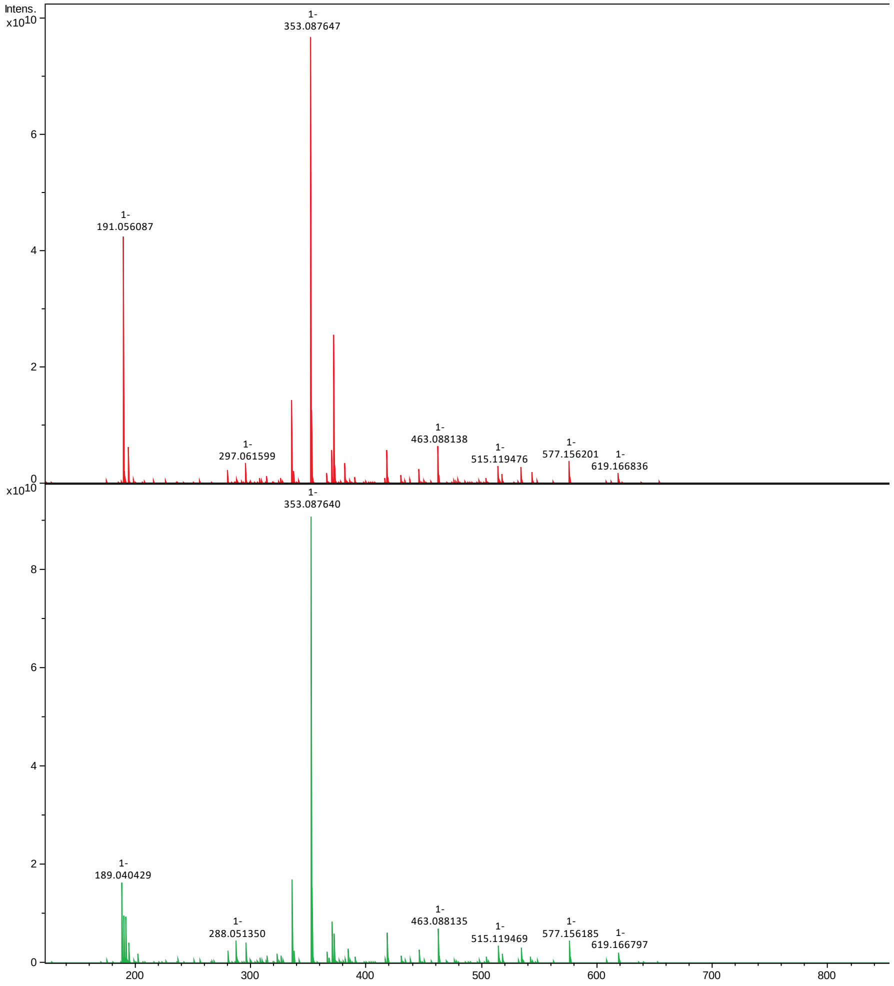

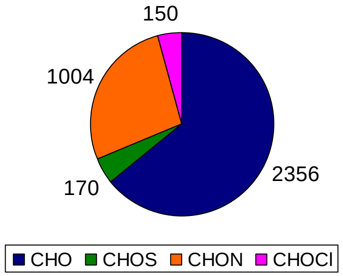

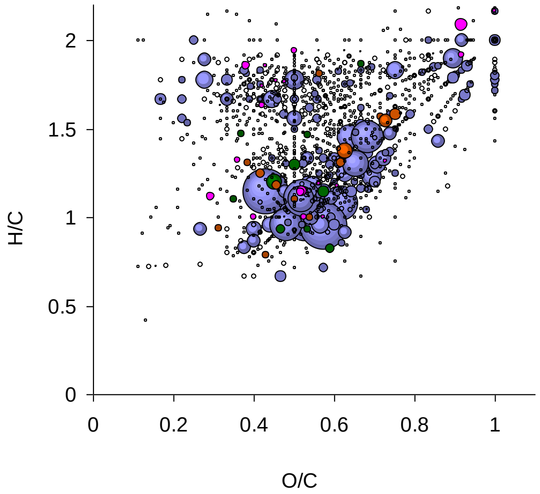

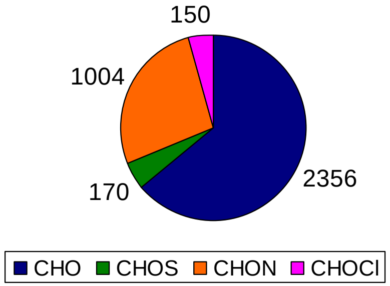

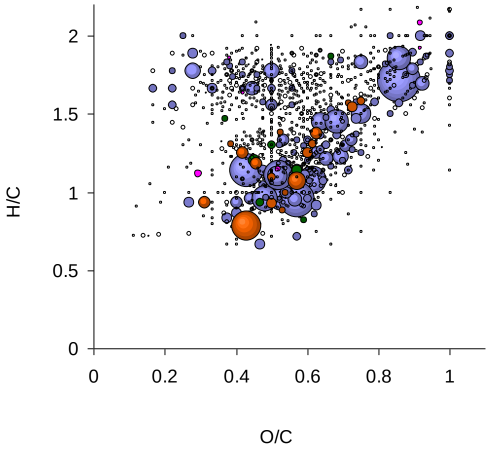

Raw dry flowering tops extracted by maceration

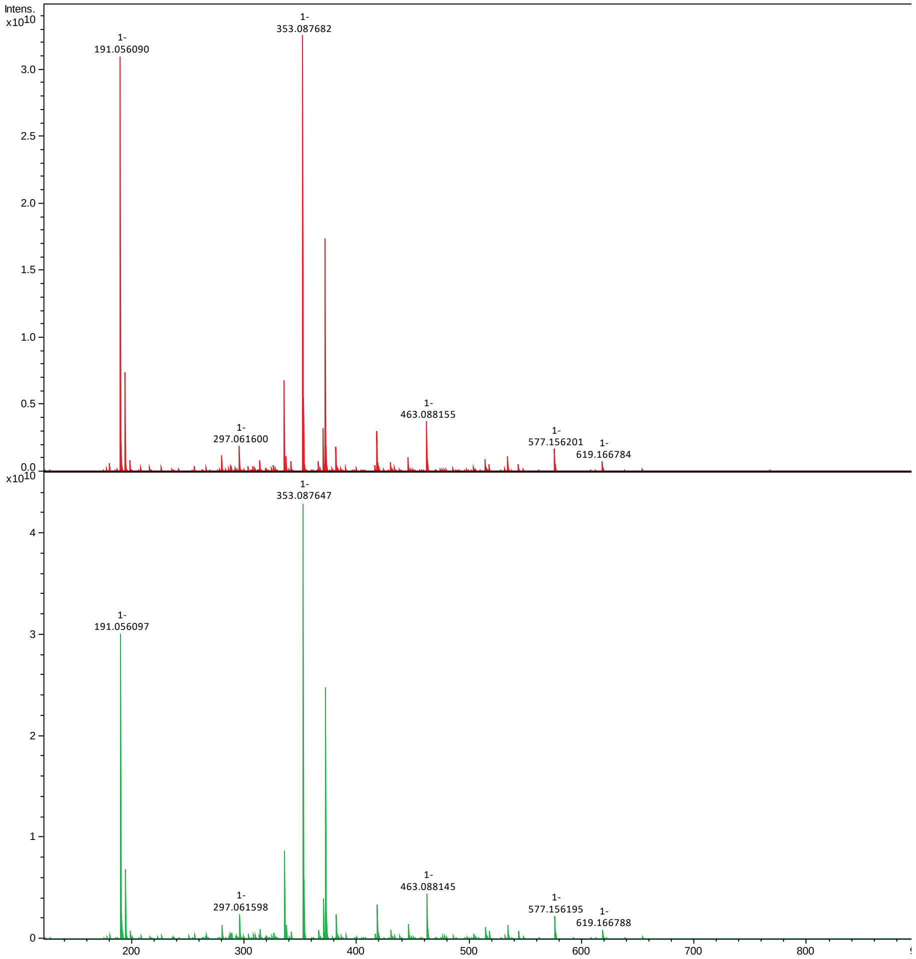

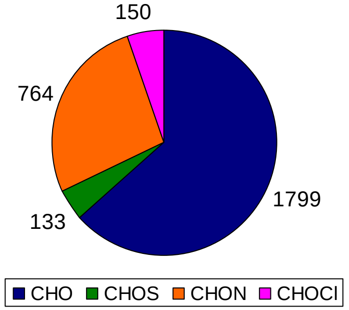

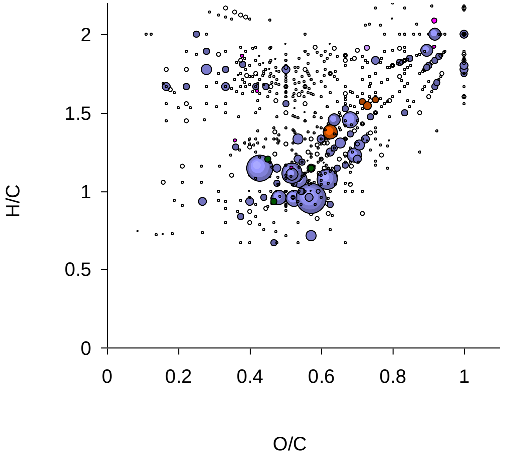

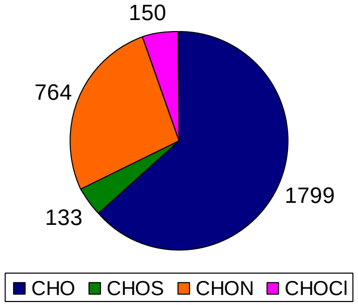

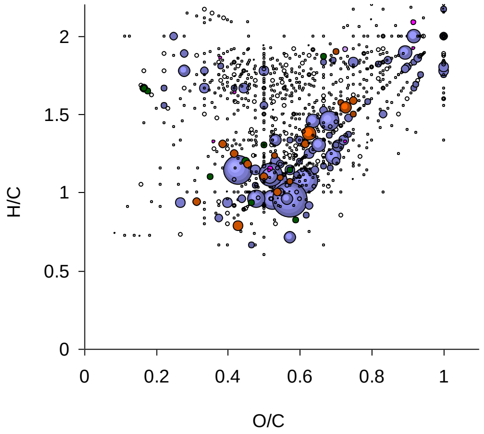

Grinded dry flowering tops extracted by microwave

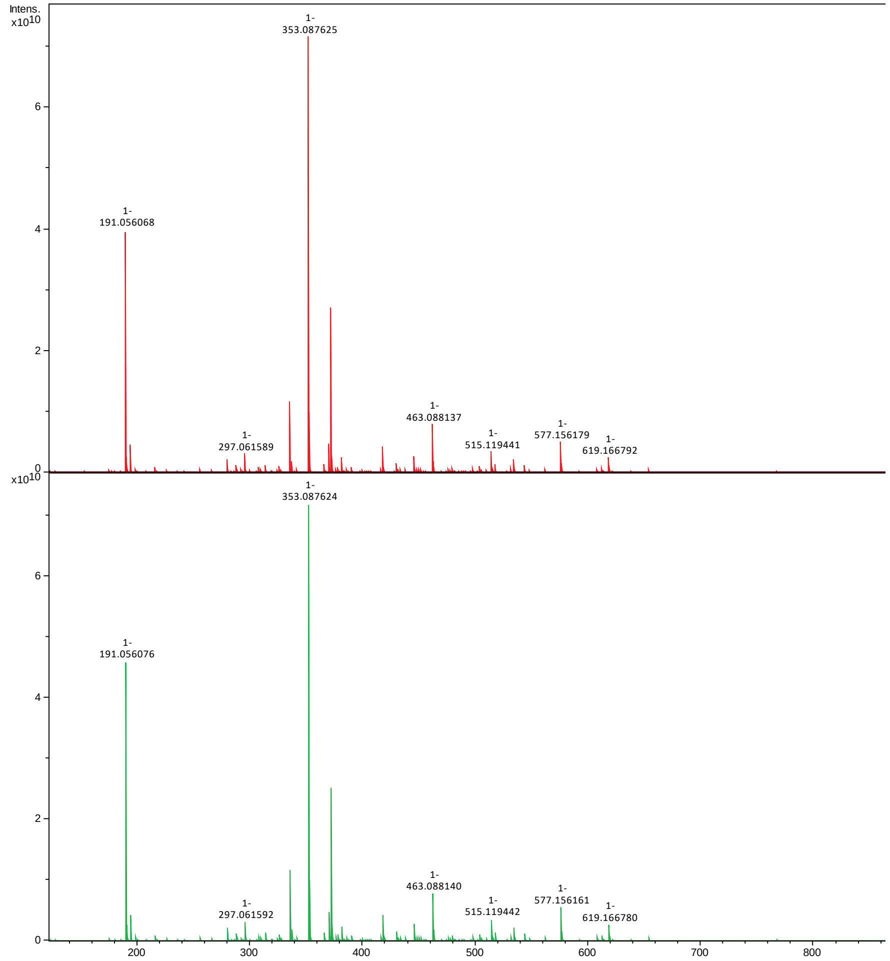

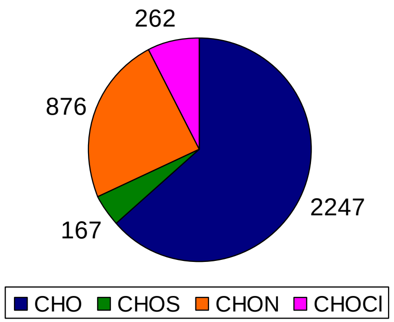

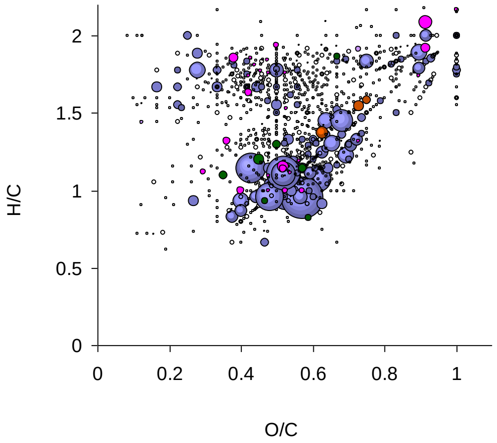

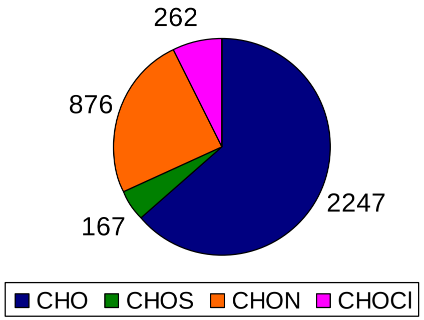

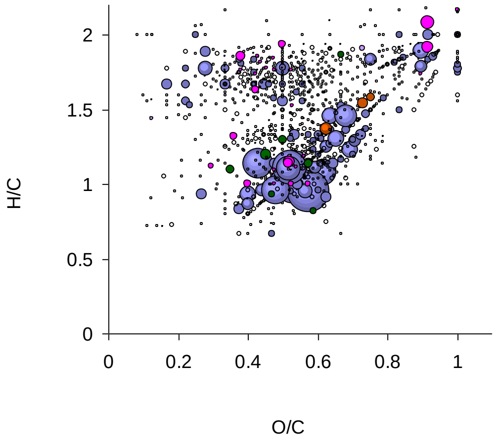

Raw dry flowering tops extracted by microwave

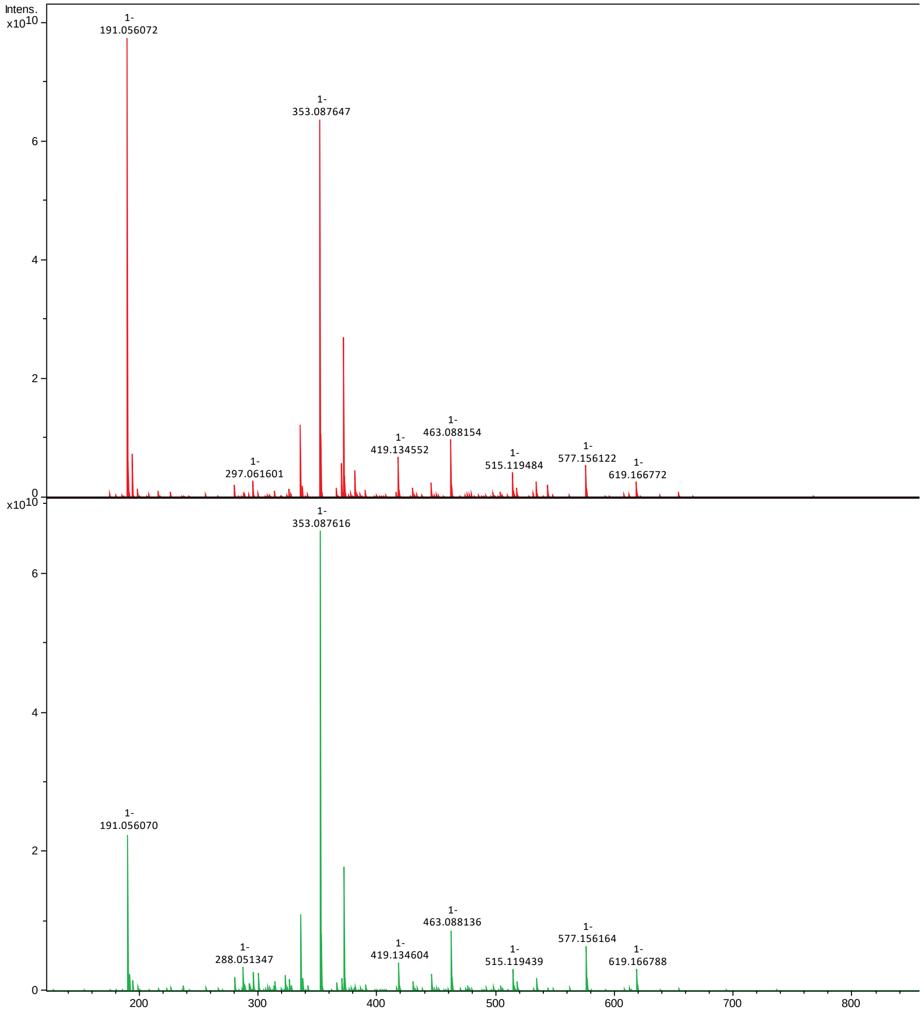

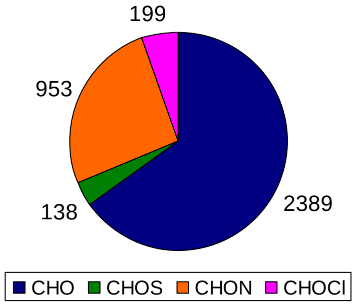

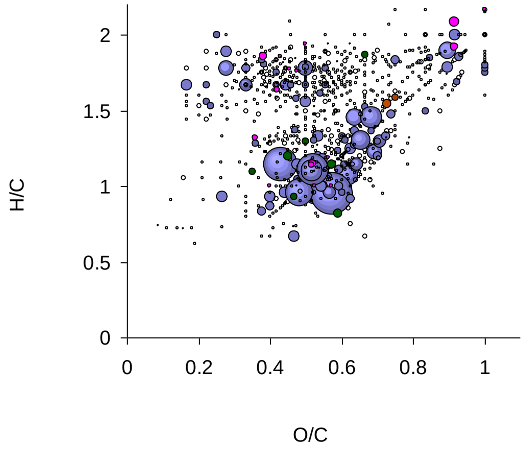

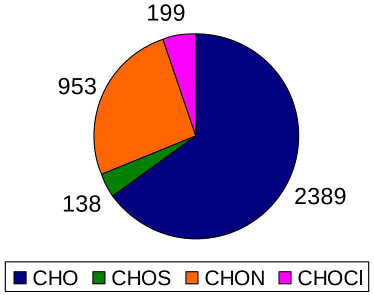

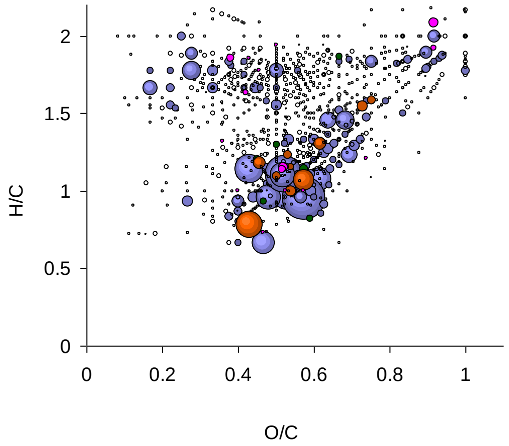

Grinded dry flowering tops extracted by percolation

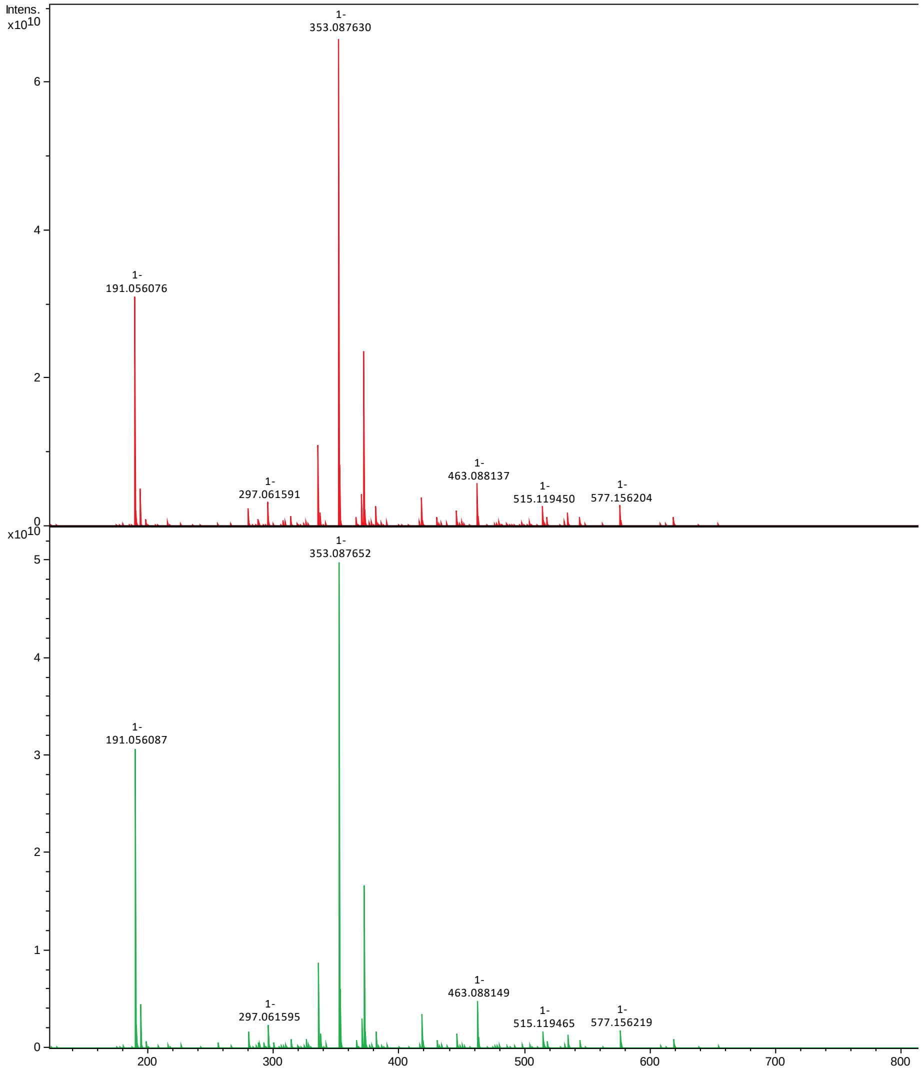

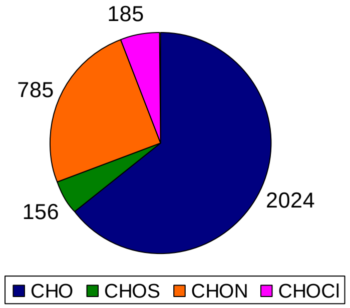

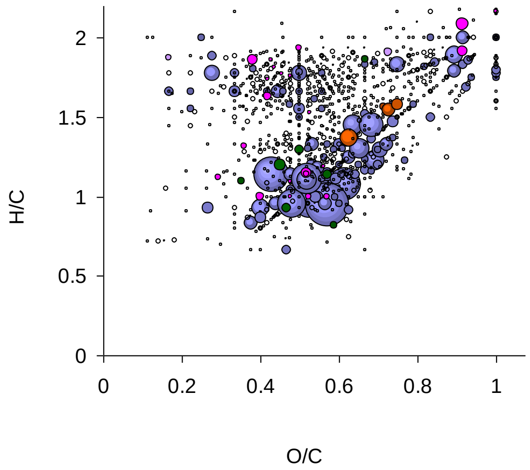

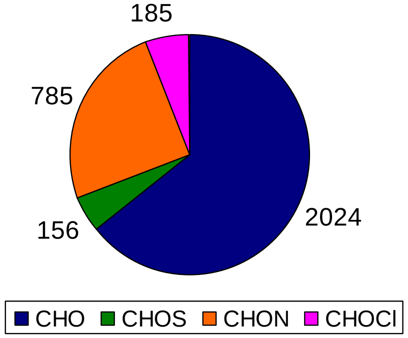

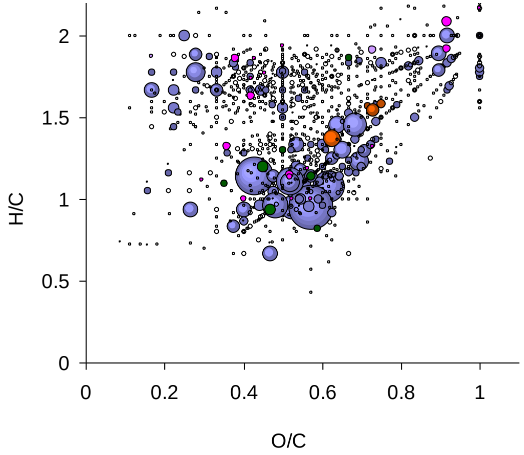

Raw dry flowering tops extracted by percolation

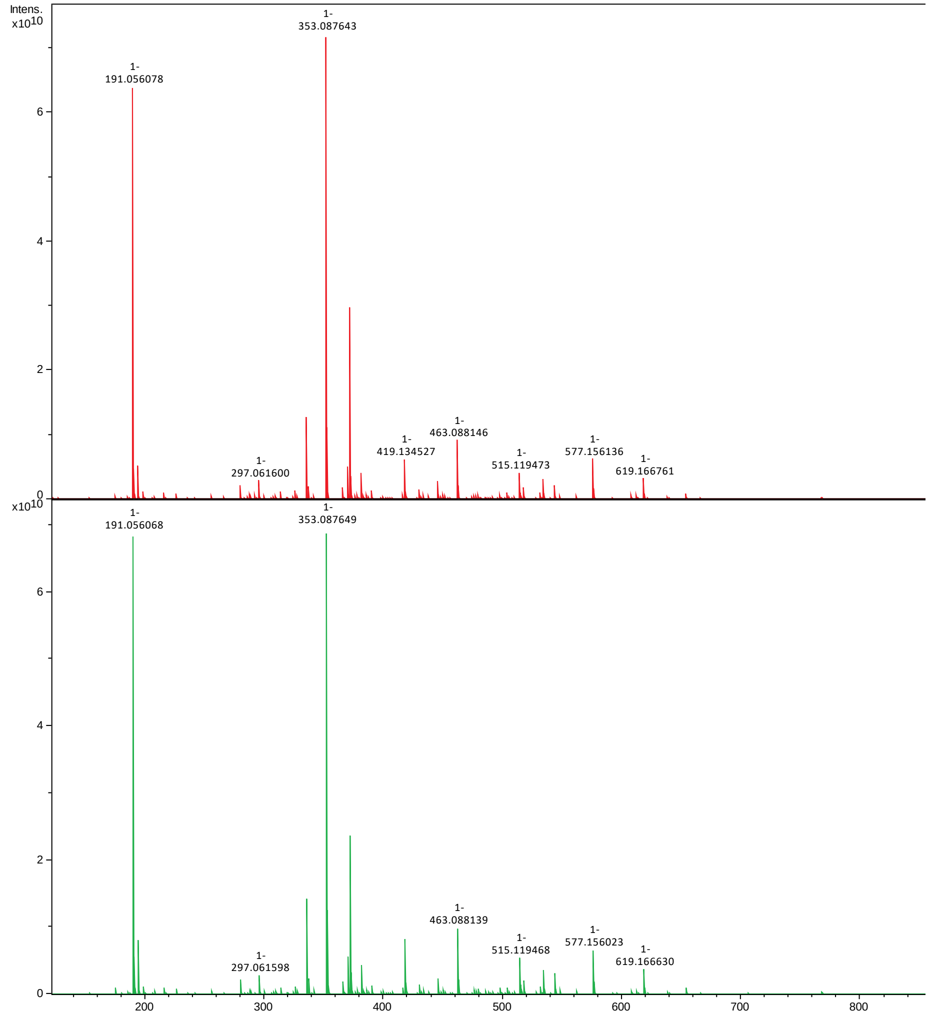

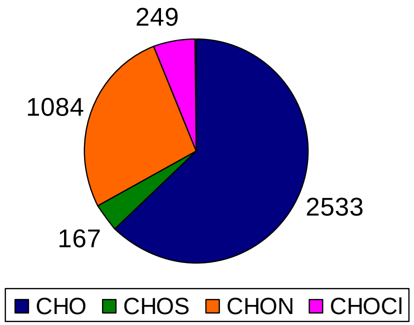

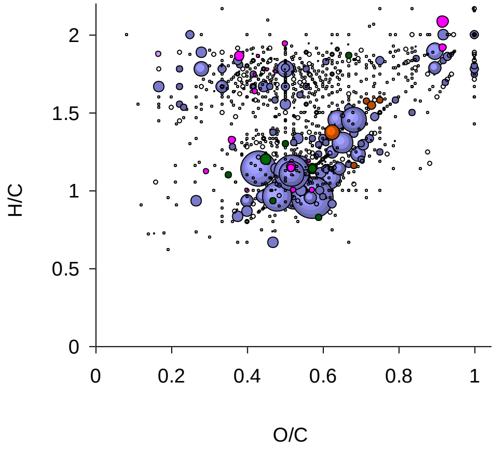

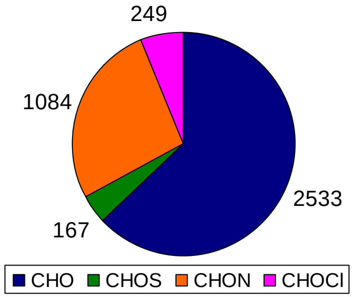

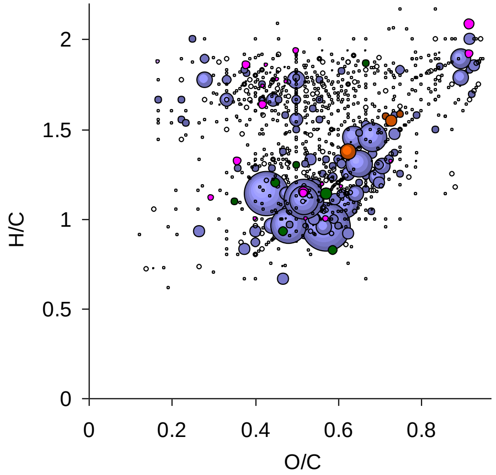

Grinded dry flowering tops extracted by ultrasonication

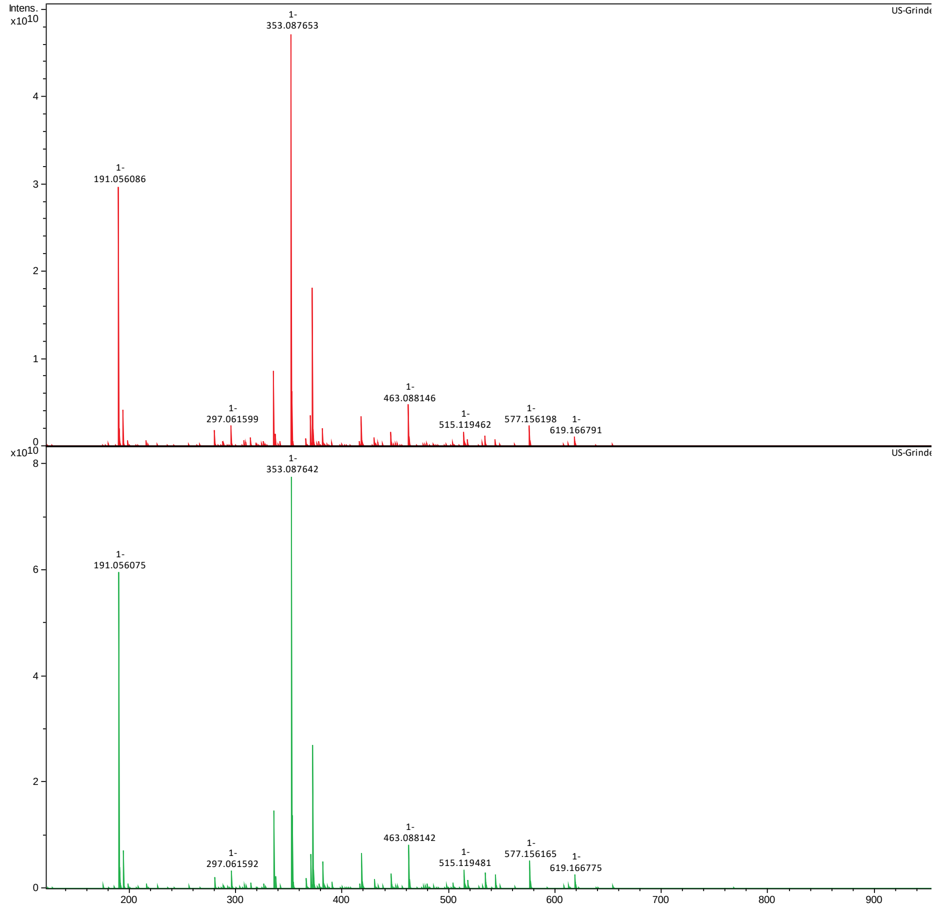

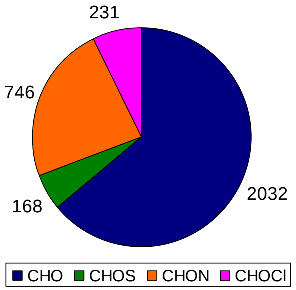

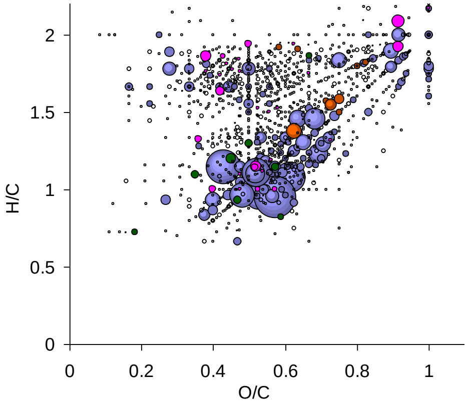

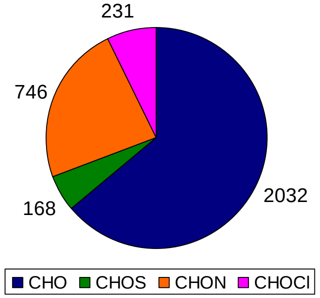

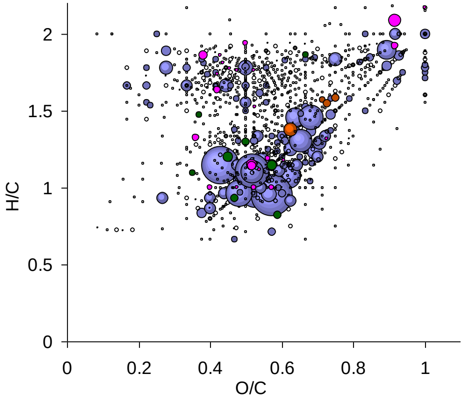

Raw dry flowering tops extracted by ultrasonication

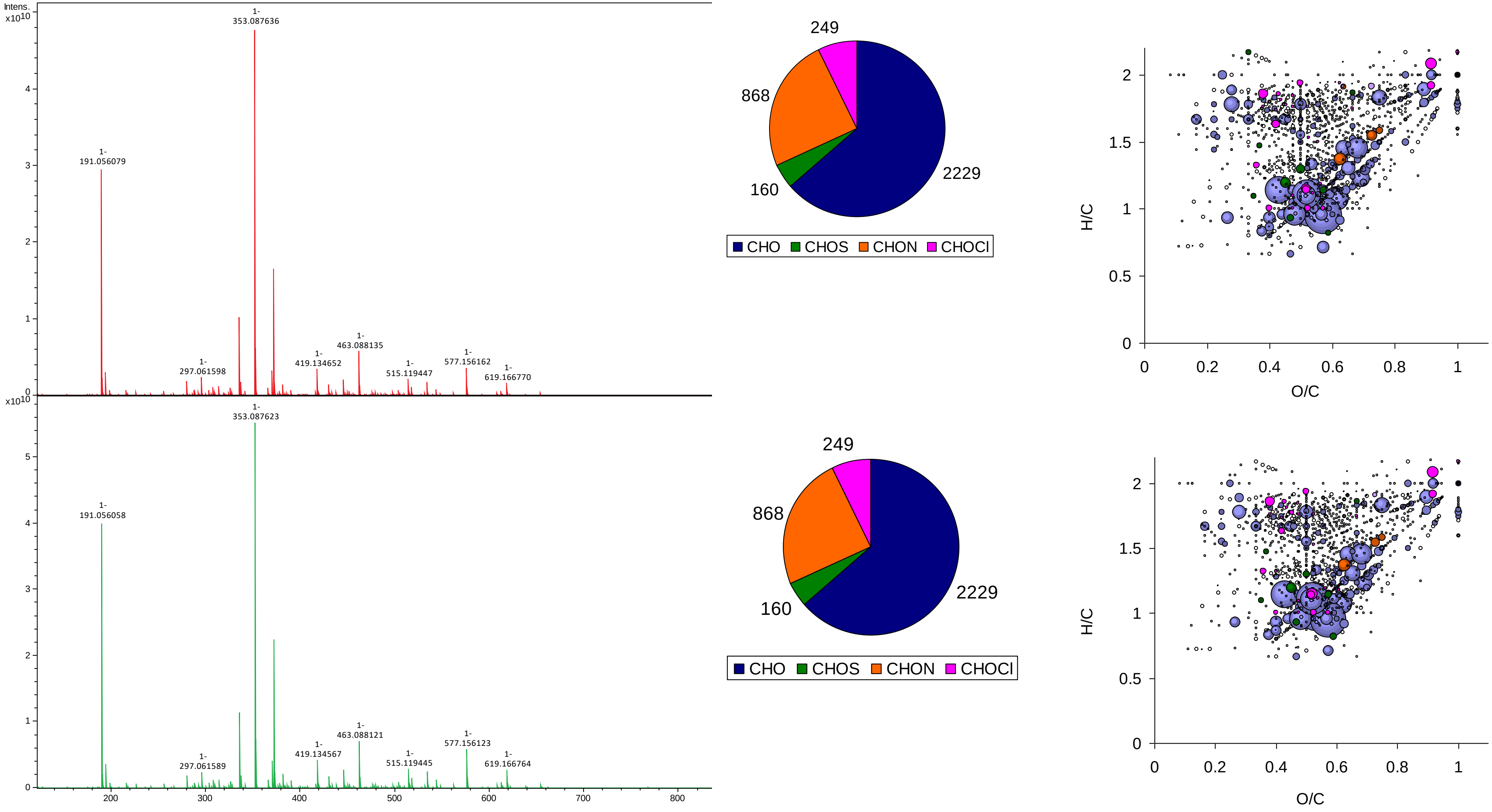

Hydroalcolic plant extract

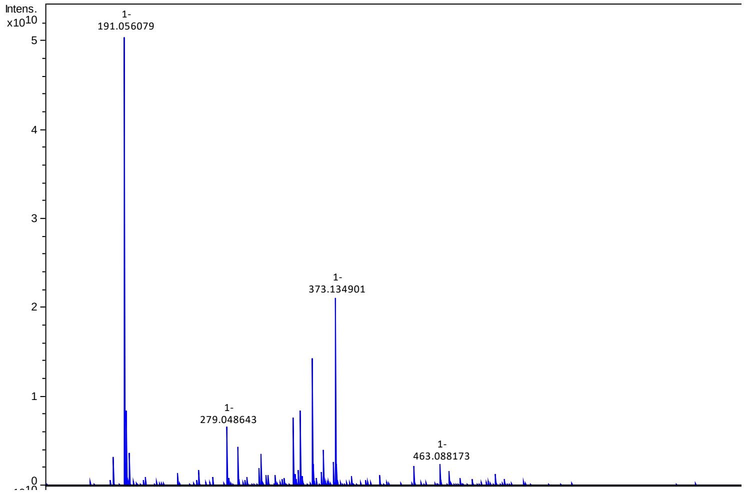

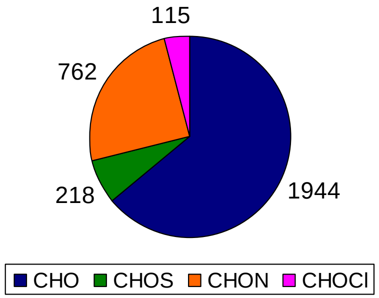

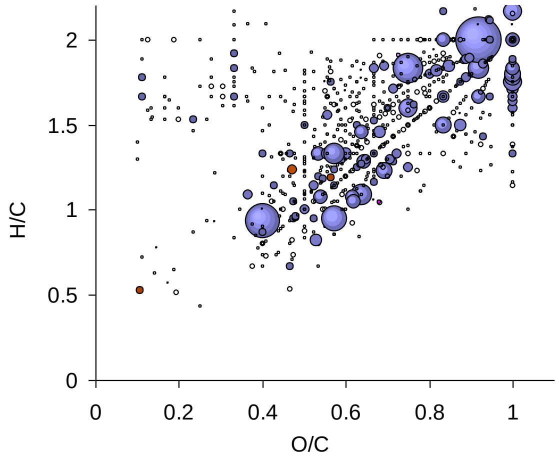

Standard plant extract

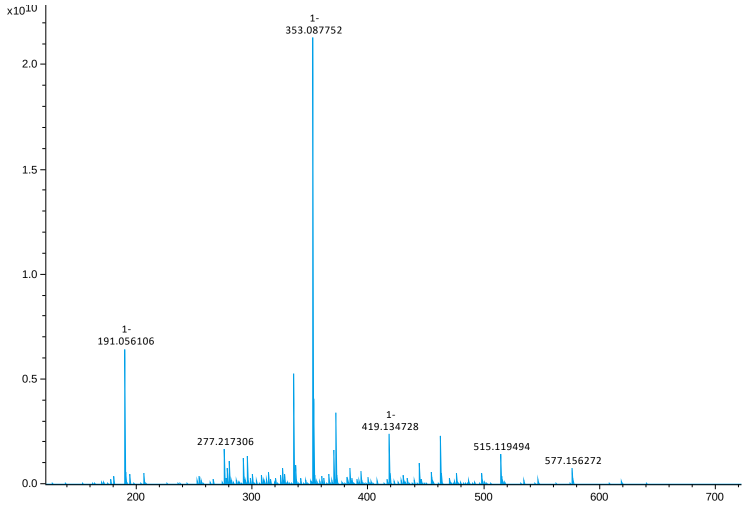

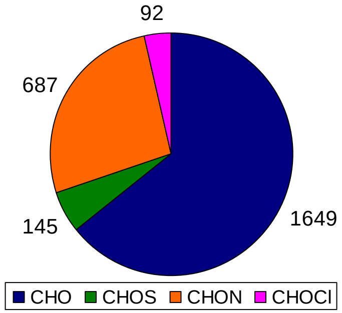

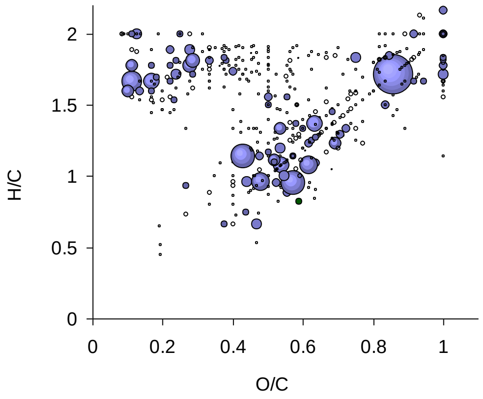

Figure S9. Hierarchical Cluster Analysis (HCA) and heatmap achieved from samples analyzed by (-) ESI FT-ICR MS. INF = Infusion. MAC = Maceration (at 60°C). PER = Percolation. US = Ultrasonic (at 60°C). MW = Microwave. Raw = raw dry flowering tops. Gr = Grinded (1 mm) flowering tops. Lot number: 20335 (flowering tops) and 20334 (flowers). 1 and 2 numbers correspond to two independent extractions of the same sample (2 repetitions).

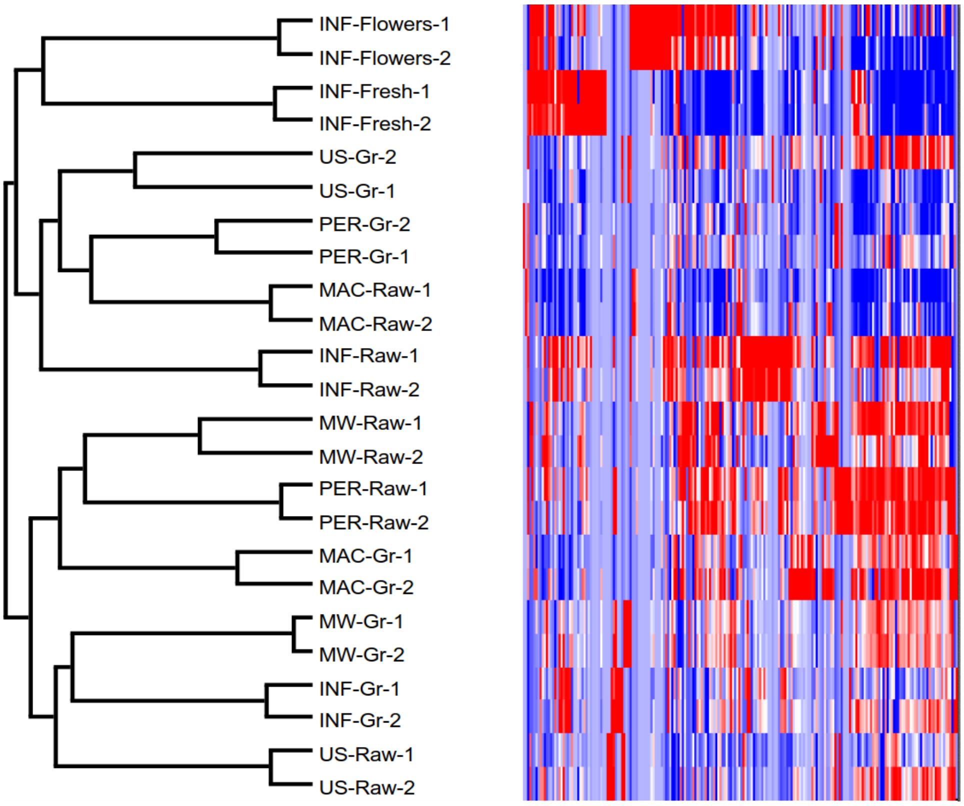

Figure S10. Pictures of cup (A), mug (B) and bowl (C) with dimensions and weights.

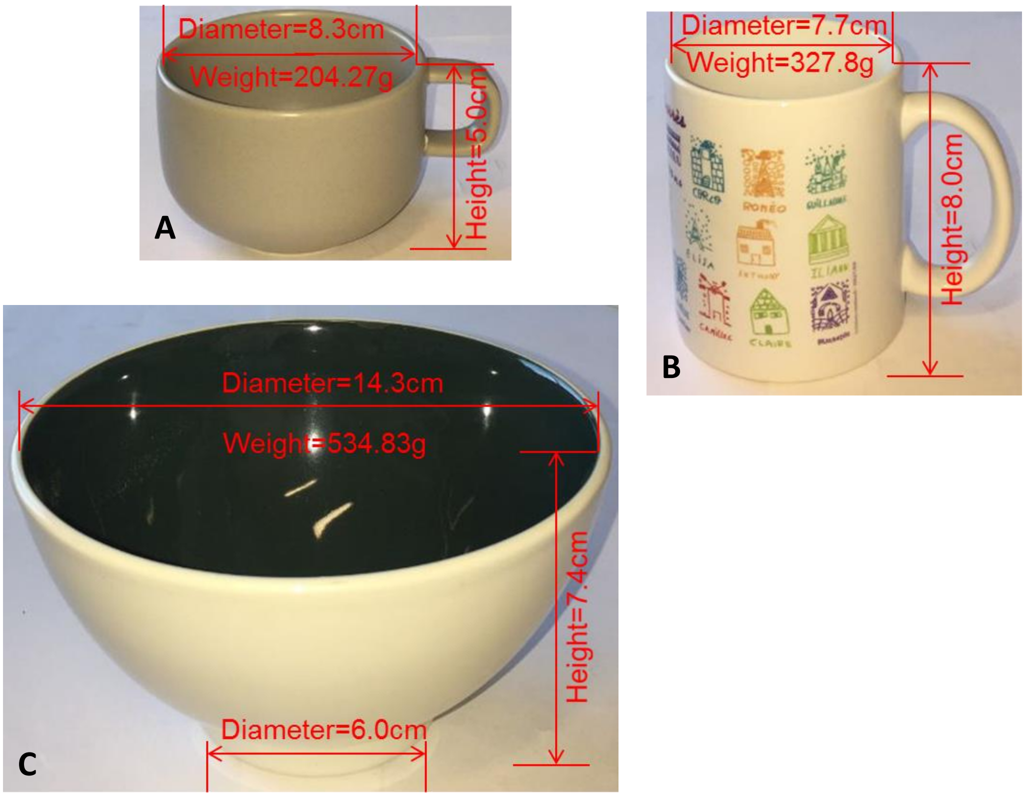

Figure S11. Decrease profile of temperature (without stirring) *vs* the nature of the container (cup, mug, bowl, Bodum®, three-neck flask). Volume used: 125 mL (cup), 250 mL (mug, Bodum® and three-neck flask), 405 mL (bowl). Lines are guides for better reading. Error bars are ± one SD on n = 3 repetitions of independent extractions.

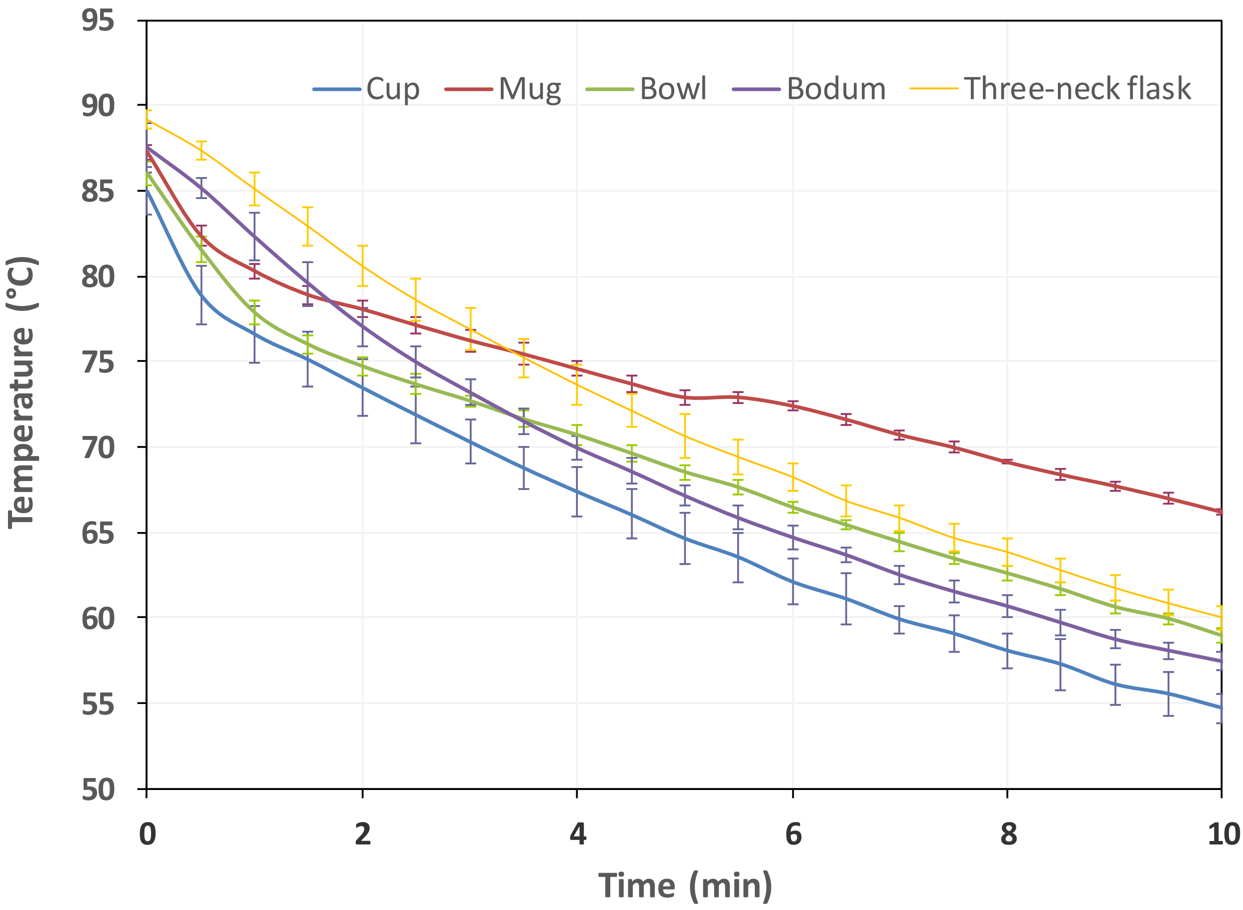

Figure S12. Pictures of raw and grinded hawthorn materials of various granulometries.

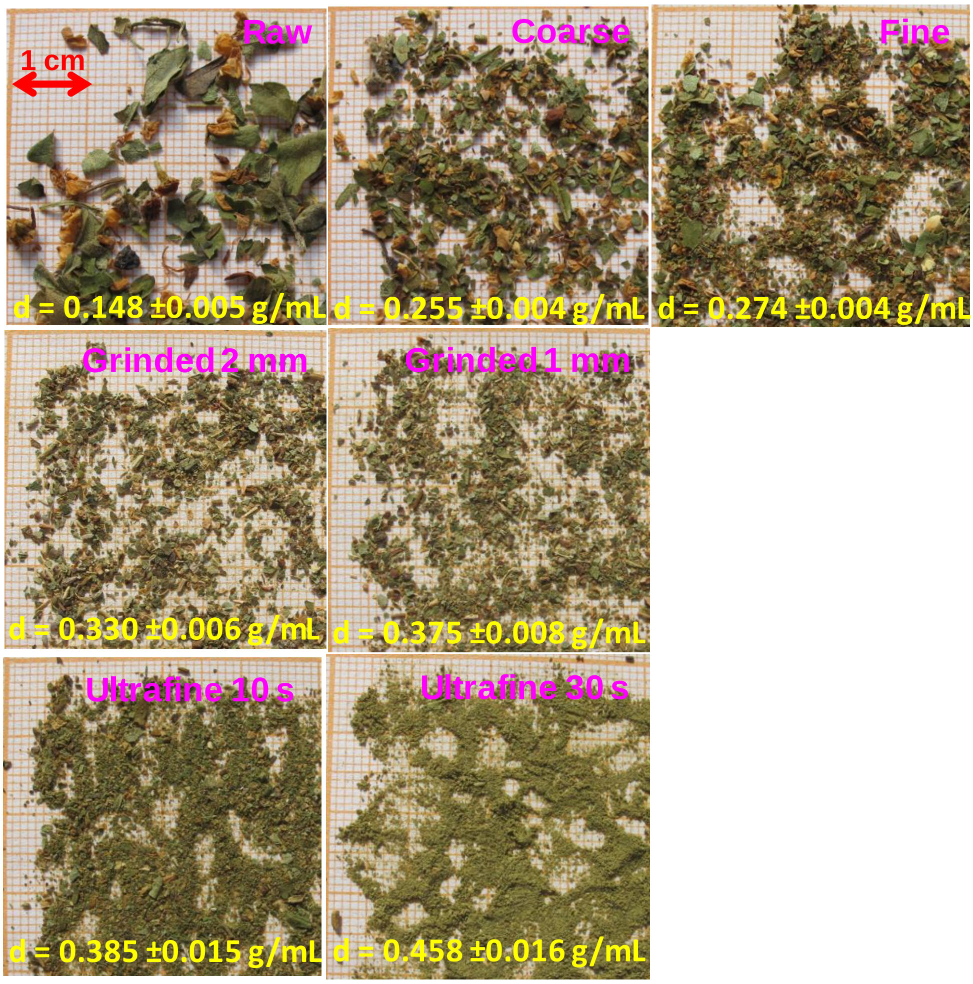

Figure S13. Size distributions of grinded hawthorn materials. (A) Fine; (B) Coarse; (C) Ultrafine 10''; (D) Ultrafine 30''; (E) Grinded 1mm; (F) Grinded 2 mm. Lot N°CB58120. Experimental condition: see section 3.2.

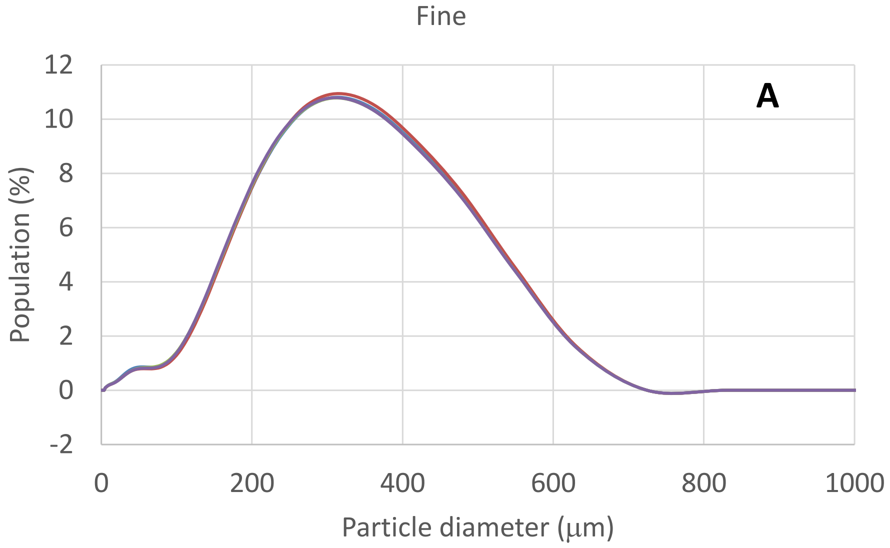

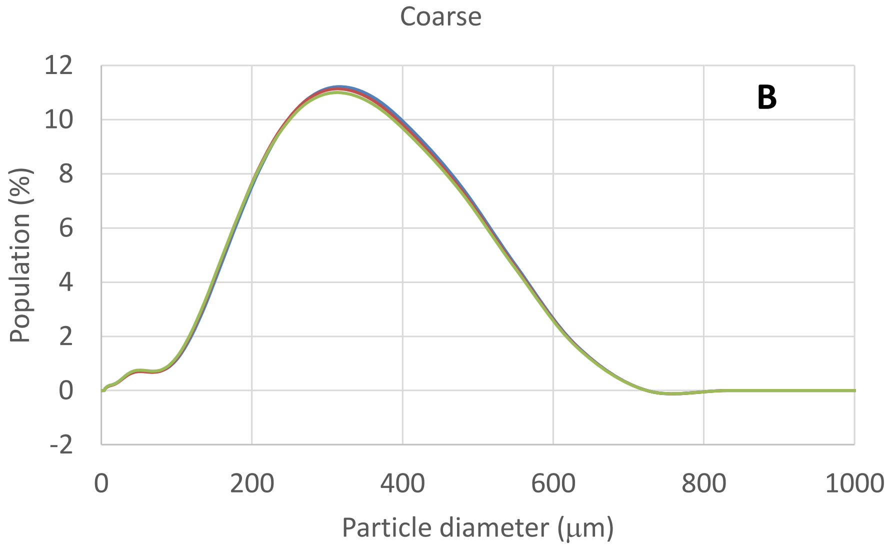

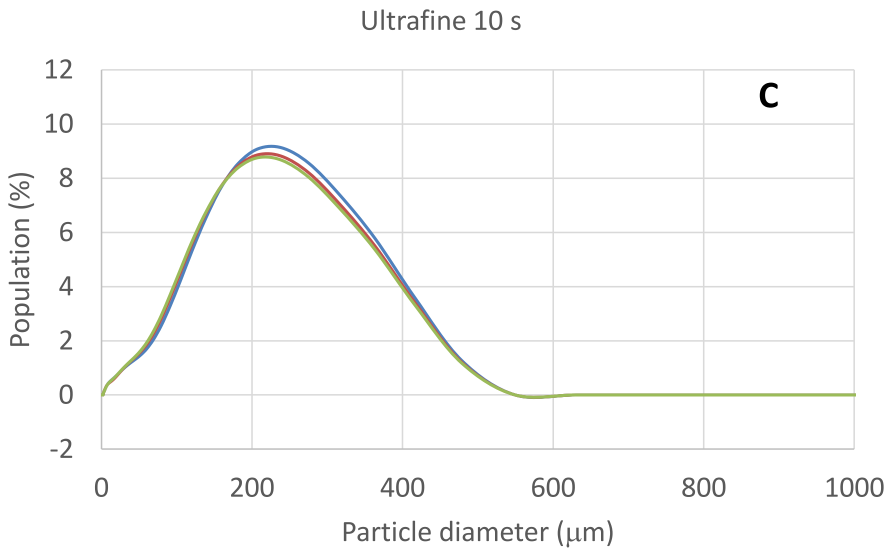
Ultrafine 10 s
C
Population (%)
Particle diameter (µm)
12
10
8
6
4
2
0
-2
0
200
400
600
800
1000

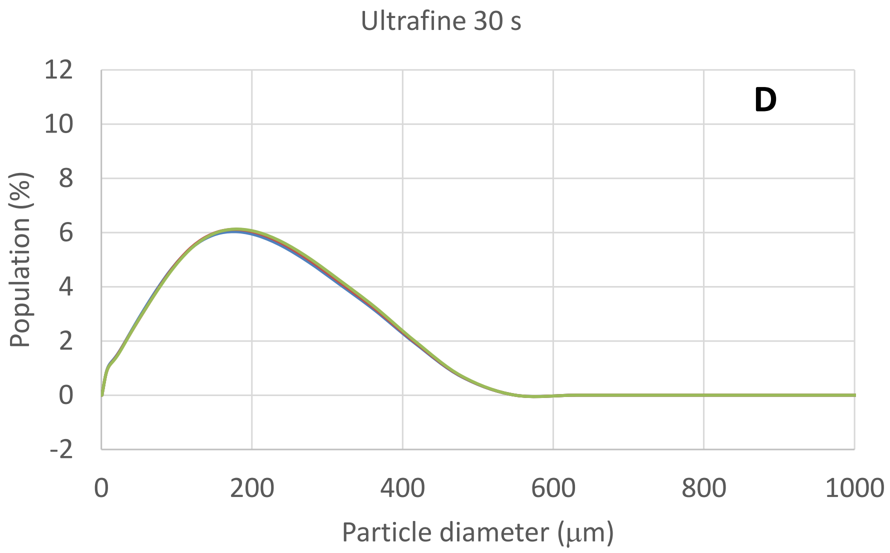
Ultrafine 30 s
D
Population (%)
Particle diameter (µm)
12
10
8
6
4
2
0
-2
0
200
400
600
800
1000

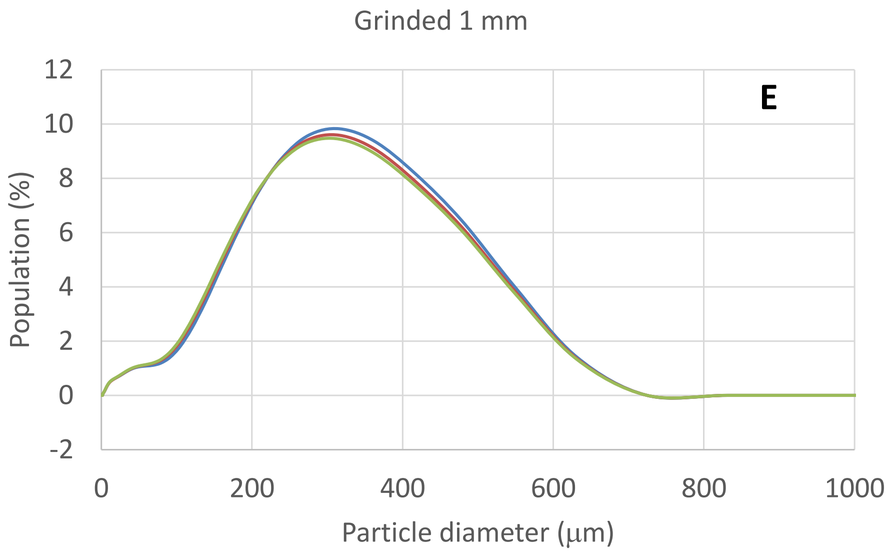
Grinded 1 mm
E
Population (%)
Particle diameter (µm)
12
10
8
6
4
2
0
-2
0
200
400
600
800
1000

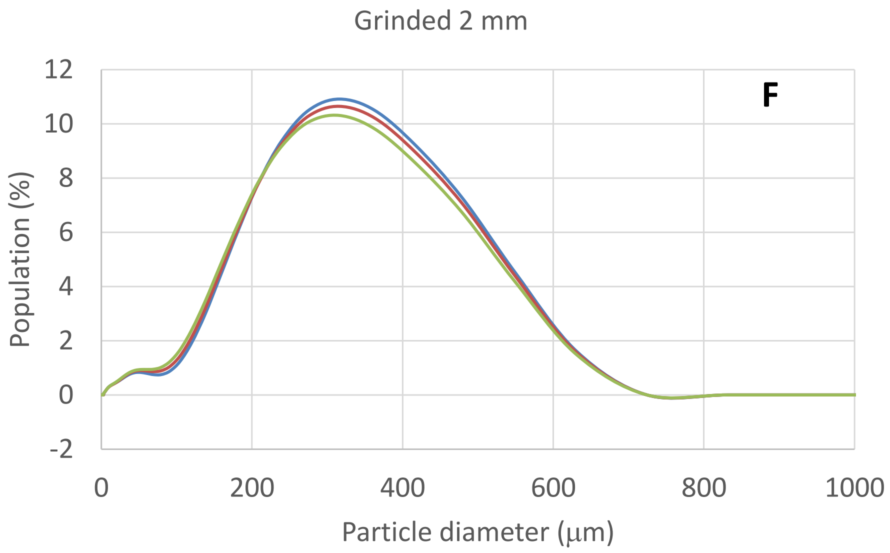
Grinded 2 mm
F
Population (%)
12
10
8
6
4
2
0
-2
0
200
400
600
800
1000
Particle diameter (μm)

Figure S14. Influence of the lot number of grinded (fine granulometry) hawthorn dry flowering tops (R78927, 1221478, H18001534, CB58120) and one lot of raw dry flowers (20334) on the UHPLC profiles of the corresponding hawthorn extracts. Infusion extraction using the optimized Bodum® set-up (see section 3.8) using 2.5 g plant infused in 250 mL water. Experimental conditions of UHPLC as in Figure S5.

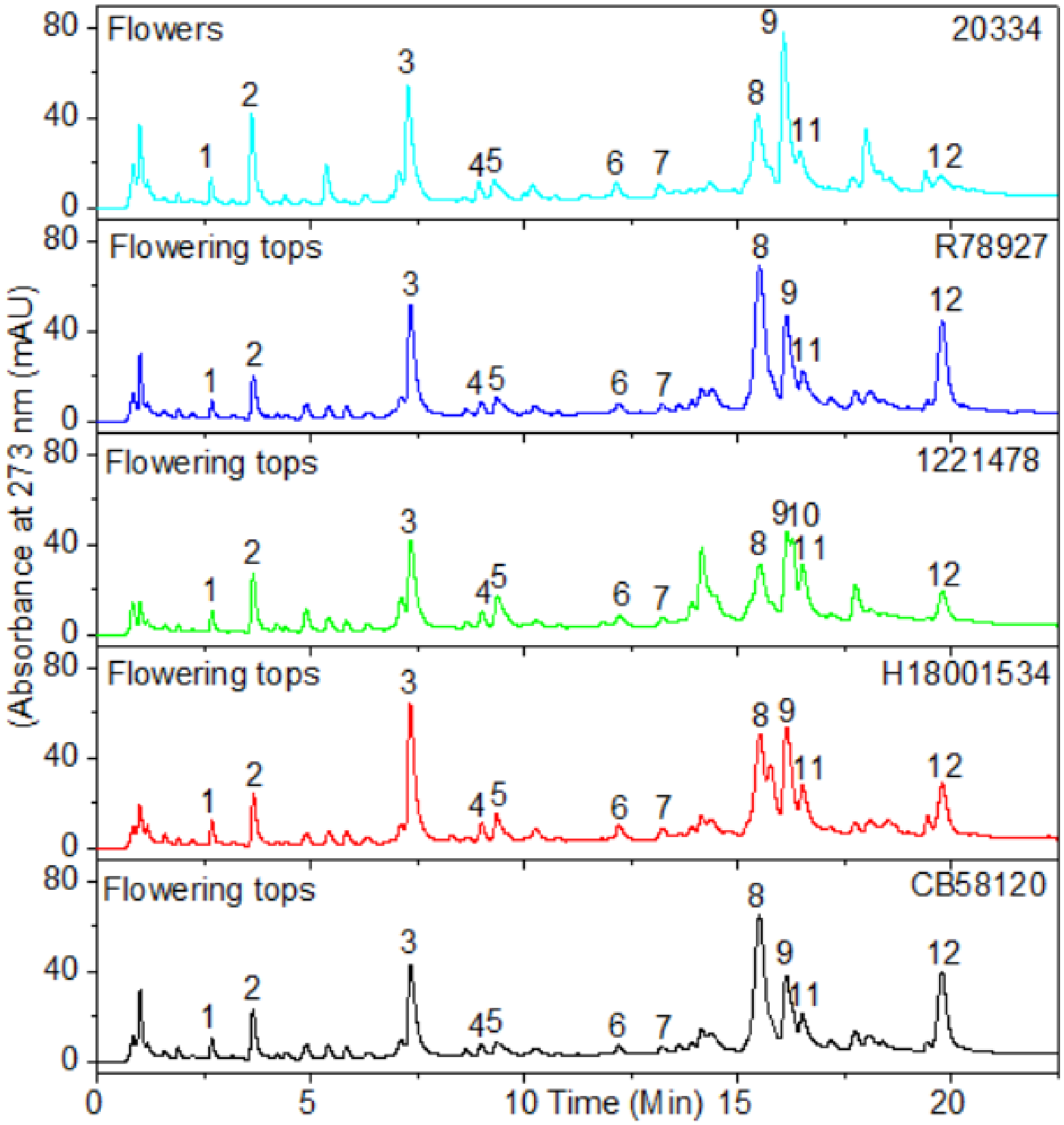

Figure S15. Relative peak area distributions for the main compounds detected by UHPLC-UV in the various grinded (fine granulometry) hawthorn extracts as a function of the lot number of dry flowering tops (R78927, 1221478, H18001534, CB58120) and one lot of raw dry flowers (20334). Same experimental conditions as in Figure S14.

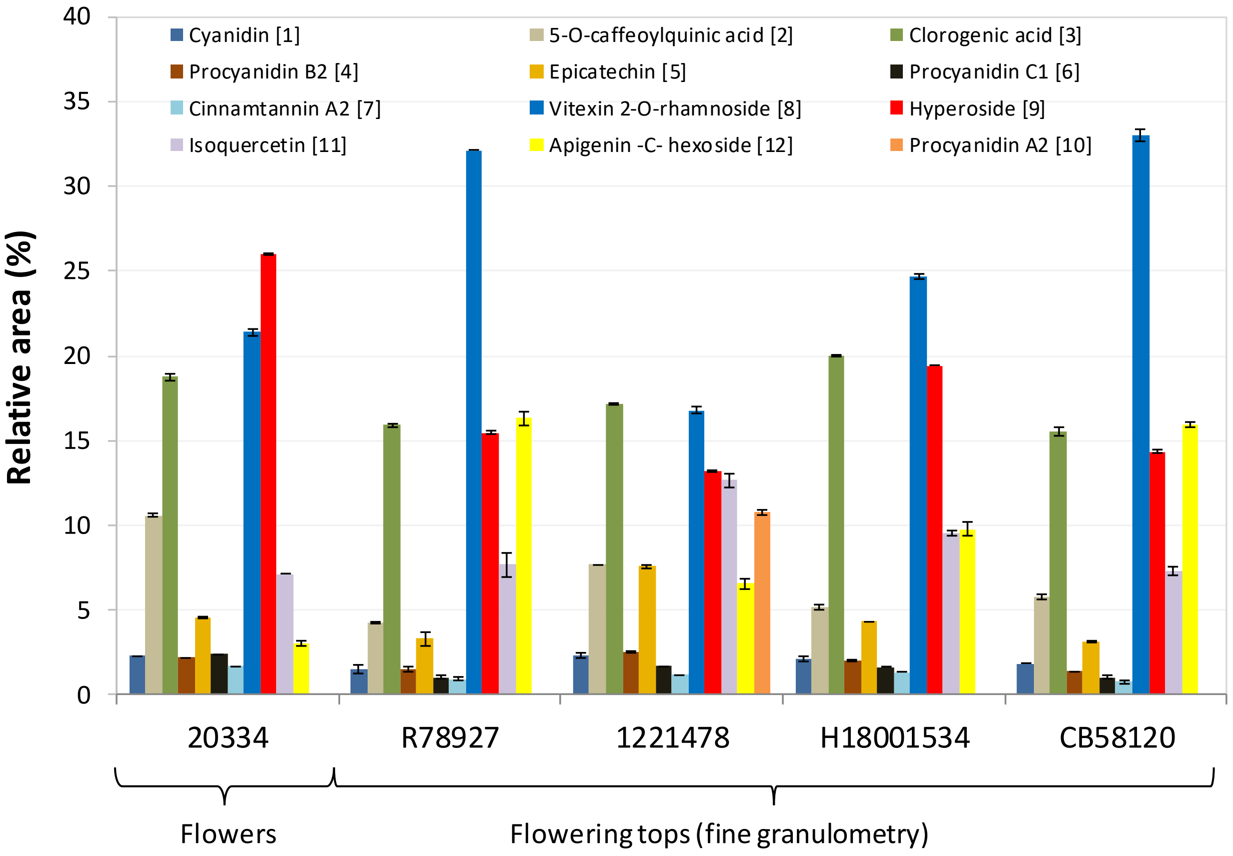

Table S1. List of the samples analyzed by UHPLC-ESI-MS and (-)ESI FT-ICR-MS. All the samples correspond to 10 min extraction time (see section 3.14 for experimental details) and were duplicated (two independent extractions).

Infusion	Granulometry/nature
Lot n°20335	Flowering tops, raw
Lot n°20335	Flowering tops, grinded 1 mm
Fresh	Flowering tops, fresh
Lot n°20334	Flowers
Maceration at 60°C	Granulometry/nature
Lot n°20335	Flowering tops, raw
Lot n°20335	Flowering tops, grinded 1 mm
US at 60°C	Granulometry/nature
Lot n°20335	Flowering tops, raw
Lot n°20335	Flowering tops, grinded 1 mm
Percolation at 60 °C	Granulometry/nature
Lot n°20335	Flowering tops, raw
Lot n°20335	Flowering tops, grinded 1 mm
MW at 300W	Granulometry/nature
Lot n°20335	Flowering tops, raw
Lot n°20335	Flowering tops, grinded 1 mm

Table S2. Fitting parameters for the absorbance A(*t*) trace *vs* extraction time *t* for the infusion mode. Extraction kinetic curves are presented Figure 1 and Figure S3. *: Temperature at 30 min extraction time.

Extraction mode	Plant	T (°C)	Stirring speed (rpm)	Stirring type	τ_1 (min)	τ_2 (min)	$A_{30\ min}$	A_{∞}	A_1
Infusion	Raw dry	41.7	250	Magnetic	1.55	21	1.407	1.627	0.640
		41.4	500		1.95	21	1.512	1.713	0.821
		40.0	750		2.3	25	1.466	1.685	0.995
		39.6	1000		1.9	30.5	1.579	1.847	1.183
	Grinded (1 mm)	41.7	250	Magnetic	0.55	26	2.274	2.400	2.050
		41.4	500		0.45	26	2.413	2.506	2.237
		40.0	750		0.4	24	2.426	2.528	2.194
		39.6	1000		0.4	26	2.517	2.658	2.272
	Fresh	41.4	500	Magnetic	2.35	32.5	0.721	1.105	0.122
Maceration	Raw dry	20	250	Mecanic	2.1	34	0.663	0.910	0.292
		40			1.95	31.5	0.803	1.100	0.320
		60			1.9	29	1.165	1.596	0.378
		80			2.0	30	1.755	2.404	0.642
	Fresh	60	500	Magnetic	1.2	36.5	0.58	0.798	0.244
	Grinded (1 mm)	20	500	Magnetic	2.1	30	1.759	2.409	0.685
		40			2.1	26	2.068	2.275	1.573
		60			0.65	30	2.301	2.531	1.904
		80			0.6	33.5	2.579	2.837	2.121
US	Raw dry	20	250	Mecanic	2.1	32	1.381	1.892	0.637
		40			2.2	38	1.63	2.233	0.967
		60			2,0	38	2.14	2.932	1.140
	Grinded (1 mm)	60	250	Mecanic	0.65	10.5	3.055	3.100	2.286

Table S3. Compounds identified in hawthorn putatively assigned to raw formulae achieved by (-) ESI FT-ICR MS.

Theoretical mass $[M-H]^-$	Putative compounds M
131.046217	Asparagine
132.030233	Aspartate
133.014249	Malic acid
137.024419	Protocatechuic aldehyde/Hydroxybenzoic acid
146.045883	Glutamate
153.019334	Protocatechuic acid
163.040069	Coumaric acid
164.071703	Phenylalanine
169.014249	Gallic acid
179.034984	Caffeic acid
179.056114	Glucose/fructose/Inositol
180.066618	Tyrosine
181.071764	Sorbitol
188.035318	alpha-cyano-4-hydroxycinnamic acid (HCCA)
191.019729	Citric acid
191.056114	Quinic acid
193.050634	Ferulic acid
203.082602	Tryptophan
223.061199	Sinapinic acid
285.040464	Kaempferol/cyanidin ($-2H^-$)
289.071764	Catechin/epicatechin
300.998994	Ellagic acid
301.035379	Quercetin
315.051029	Sexangularetin
331.067074	Galloylglucose
341.108939	Sucrose
353.087809	Chlorogenic acid / 5-O-Caffeoylquinic acid
385.092894	Diferulic acid
413.087809	Pinnatifida A/C
417.082724	Kaempferol-O-arabinoside (crataegide)
431.098374	Vitexin / Isovitexin / Apigenin-C-hexoside
433.077639	Quercetin pentoside
447.093289	Orientin / Luteolin-7-O-glucuronide/ Ideain / Methoxykaempferol-pentoside / Luteolin-C-hexoside
455.098374	Pinnatifida B/D
455.353069	Oleanolic or ursonic acid
461.072554	Luteolin-7-O-glucuronide
461.108939	Methyl luteolin-C-hexoside
463.088204	Hyperoside/Isoquercitin/Spiraeoside
473.072554	Chicoric acid
473.108939	Acetyl vitexin
477.103854	Sexangularetin-3-O-glucoside
483.078034	Digalloylglucose
489.103854	Acetylorientin
505.098769	Quercetin acetyl hexoside
563.104249	Sexangularetin-3-O-(malonyl) glucoside
563.140634	Schaftoside
575.119504	Procyanidin A2
577.135154	Procyanidin B2
577.156284	Iso/Vitexin 2-O-rhamnoside
593.151199	Vincenin / Keampferol-3-O-neopheridoside / Iso/Orientin-O- rhamnoside
609.146114	Rutin / Quercetin-3-O-rhamnosylgalactoside
619.166849	Vitexin acetyl rhamnoside
623.161764	Sexangularetin-3-O-neohesperidoside / metoyxykaepferol methylpentosylhexoside
755.204024	Quercetin di rhamnosyl hexoside/ Rhamnosyl rutin
771.198939	Vitexin-di-O-glucoside
865.198544	Procyanidin C1

Table S4. Putative compounds obtained from features specifically extracted depending on the plant states (fresh vs dry and grinded vs raw) or parts (flowers vs flowering tops). See Table S1 for the lot numbers.

	Fresh vs. Dry flowering tops		Dry Flower vs. dry Flowering tops		Grinded vs. Raw Flowering tops	
Order*	Fresh	Dry	Flowers	Flowering tops	Grinded	Raw
1	Sucrose	Chlorogenic acid / 5-O-Caffeoylquinic acid	Sexangularetin-3-O-glucoside	Orientin / Luteolin-7-O-glucuronide/ Ideain / Methoxykaempferol-pentoside / Luteolin-C-hexoside	Chlorogenic acid / 5-O-Caffeoylquinic acid	Ferulic acid
2	Sexangularetin-3-O-(malonyl) glucoside	Orientin / Luteolin-7-O-glucuronide/ Ideain / Methoxykaempferol-pentoside / Luteolin-C-hexoside	Kaempferol-O-arabinoside (crataegide)	Vitexin acetyl rhamnoside	Catechin/Epicatechin	
3	Acetylorientin	Vitexin acetyl rhamnoside	Schaftoside	Galloylglucose	Procyanidin B2	
4	Quercetin pentoside	Vitexin/Isovitexin/Apigenin-C-hexoside	Quercetin pentoside	Luteolin-7-O-glucuronide	Coumaric acid	
5	Oleanolic/ursonic acid	Schaftoside	Vincenin / Keampferol-3-O-neopheridoside / Iso/Orientin-O-rhamnoside	Ellagic acid	Procyanidin A2	
6	Pinnatifida A/C	Galloylglucose	Coumaric acid		Diferulic acid	
7	Procyanidin A2	Malic acid	Malic acid		Pinnatifida B/D	
8			Tryptophan		Protocatechuic aldehyde/hydroxybenzoic acid	
9			Glutamate		Glutamate	
